# Central venous access device terminologies, complications, and reason for removal in oncology: a scoping review

**DOI:** 10.1186/s12885-024-12099-8

**Published:** 2024-04-19

**Authors:** Kerrie Curtis, Karla Gough, Meinir Krishnasamy, Elena Tarasenko, Geoff Hill, Samantha Keogh

**Affiliations:** 1 Department of Nursing, University of Melbourne, Melbourne, Australia; 2 Peter MacCallum Cancer Centre, Melbourne, Australia; 3 Austin Health, Melbourne, Australia; 4 Department of Health Services Research, Peter MacCallum Cancer Centre, Melbourne, Australia; 5 Sir Peter MacCallum Department of Oncology, University of Melbourne, Melbourne, Australia; 6 Victorian Comprehensive Cancer Centre Alliance, Melbourne, Australia; 7 Royal Melbourne Hospital, Melbourne, Australia; 8 Centre for Healthcare Transformation, Queensland University of Technology, Brisbane, Australia

**Keywords:** Central venous catheters, Catheters indwelling, Central venous access device, Device removal, Complication, Premature removal, Terminology

## Abstract

**Background:**

Lack of agreed terminology and definitions in healthcare compromises communication, patient safety, optimal management of adverse events, and research progress. The purpose of this scoping review was to understand the terminologies used to describe central venous access devices (CVADs), associated complications and reasons for premature removal in people undergoing cancer treatment. It also sought to identify the definitional sources for complications and premature removal reasons. The objective was to map language and descriptions used and to explore opportunities for standardisation.

**Methods:**

A systematic search of MedLine, PubMed, Cochrane, CINAHL Complete and Embase databases was performed. Eligibility criteria included, but were not limited to, adult patients with cancer, and studies published between 2017 and 2022. Articles were screened and data extracted in Covidence. Data charting included study characteristics and detailed information on CVADs including terminologies and definitional sources for complications and premature removal reasons. Descriptive statistics, tables and bar graphs were used to summarise charted data.

**Results:**

From a total of 2363 potentially eligible studies, 292 were included in the review. Most were observational studies (*n* = 174/60%). A total of 213 unique descriptors were used to refer to CVADs, with all reasons for premature CVAD removal defined in 84 (44%) of the 193 studies only, and complications defined in 56 (57%) of the 292 studies. Where available, definitions were author-derived and/or from national resources and/or other published studies.

**Conclusion:**

Substantial variation in CVAD terminology and a lack of standard definitions for associated complications and premature removal reasons was identified. This scoping review demonstrates the need to standardise CVAD nomenclature to enhance communication between healthcare professionals as patients undergoing cancer treatment transition between acute and long-term care, to enhance patient safety and rigor of research protocols, and improve the capacity for data sharing.

**Supplementary Information:**

The online version contains supplementary material available at 10.1186/s12885-024-12099-8.

## Background

Central venous access devices (CVADs) are critical for effective and efficient management of patients with malignancies because they facilitate urgent, acute or prolonged access to the bloodstream for the administration of prescribed and supportive therapies and repeated blood sampling [1]. However, they also present considerable risk of complications and many are removed prematurely before the end of prescribed therapy. Premature removal rates of up to 50% are reported in this patient cohort [1–3]. Complications can be related to the coagulopathic and inflammatory processes of the disease process [4], adverse effects of prescribed therapies including prolonged and profound immunosuppression [3], and adverse effects of supportive therapies such as blood products [1]. CVAD complications and premature removal may lead to delays in treatment, reduced treatment efficacy and subsequent survival due to interruptions in schedules [5], and increased morbidity from CVAD complications (e.g., infection, mortality and healthcare expenditure) [1].

Lack of standardised nomenclature in healthcare has been shown to negatively impact patient safety, patient experience and health system efficiency [6]. The lack of a common language impairs communication and interoperability between individuals and organisations [6]. The potential for complex systems such as electronic health records (EHR) to accurately capture clinical management of patients’ care and health outcomes [7] and to inform and support research is reliant on agreed nomenclature. This enables data sharing, robust data analysis, and meets the requirements of a learning health system [8]. An example of a common global language used in healthcare is the systematised nomenclature of medicine clinical terms (SNOMED CT). SNOMED CT is a comprehensive and precise medical terminology system that is coded and linked, facilitating homogenous data entry, encoding of existing data, mapping of free text, analysis of clinical data, and interoperability between systems and organisations [9].

To date, there is no consensus on CVAD terminology and no standardised definitions for CVAD associated complications and reasons for premature removal. This is imperative to advance the quality and safety of clinical assessment and management, and to drive robust, impactful research for patients undergoing cancer treatment. A scoping review fits well with reviews that map and synthesise available evidence about a given topic and identify gaps and similarities in the published literature [10]. The aim of this review was to understand the terminologies used to describe CVADs, associated complications and reasons for premature removal in people undergoing cancer treatment. It also sought to identify the definitional sources for complications and premature removal reasons. The objective was to map language and descriptions used and to explore opportunities for standardisation.

## Methods

### Protocol

An a priori protocol for this scoping review aligning with the five stages of Arksey and O’Malley’s scoping review framework, including identification of the research question and relevant studies, selection of studies, documentation of the data, and collating and summarising the results, was developed. Reporting was guided by the PRISMA Extension for Scoping Reviews, PRISMA-ScR [11].

### Eligibility criteria

Adult patients with cancer over the age of 18 years and with any type of CVAD in situ, for example short-term centrally inserted central catheters (CICCs), or longer term CVADs, for example peripherally inserted central catheters (PICCs) or totally implantable venous access devices (TIVADs) were eligible for inclusion. In keeping with the broad aims of a scoping review, study designs included experimental, quasi-experimental, observational, systematic reviews, meta-analyses, quality improvement and surveys. Studies were limited to English and publications after the 2016 edition of the Infusion Therapy Standards of Practice [12].

### Information sources

The search was executed in the MedLine, PubMed, Cochrane, CINAHL Complete and Embase databases for a comprehensive approach to the topic.

### Search

#### Population, concept, and context

The search strategy was developed in collaboration with a medical librarian to address the question: how are reasons for premature removal and CVAD-related complications defined in the published literature? A second question was established in response to the diversity of CVAD terminologies noted during development of the search strategy: what CVAD terminology is evident in the published literature? The broader approach of a scoping review aligns with a less restrictive search strategy based on the population, concept and context (PCC) format compared to the precise research questions, and inclusion and exclusion criteria required for a systematic review [13]. The population for this review was broad, including all patients with haematological and solid tumours as this cohort requires insertion of a CVAD for the administration of prescribed therapies for treatment of their disease.

The concept in this scoping review included the various CVAD-related complications and reasons for premature removal. This was not restricted to the more commonly reported issues of infection and thrombosis and included subject headings and key terms for clinically relevant problems such as occlusion, catheter migration, skin impairment, CVAD damage or rupture, and accidental dislodgement. Categorical descriptors (e.g., equipment failure, device removal, accidental injuries, and death) were also included.

The context was patients with any type of CVAD in situ as the different CVAD types serve different functions according to the goals of treatment, and type and length of prescribed therapies. CVADs included CICCs, PICCs, tunnelled cuffed-centrally inserted central catheters, totally implantable venous access ports, and apheresis and haemodialysis catheters. Subject headings (e.g., central venous catheters or catheterization, central venous), descriptors (e.g., cuff, tunnelled, implanted), trade names commonly used in the literature (e.g., Hickman™ or Infusaport™) were included.

The search was established for the MEDLINE database (Table 1), then adapted for PUBMed – National Institutes of Health (NIH), EMBASE, CINAHL and the Cochrane Library.

Subject headings and key words were combined using Boolean operators AND/OR. The search limiters applied were publication dates before 2017, non-English language, and studies in animals (including mice, mouse, rat(s), porcine, pig(s), sheep, murine, canine or rabbit) or in vitro. Excluded study designs were qualitative studies, study protocols and study reports with limited information including conference abstracts, letters to the editor, educational, posters and case studies.

### Selection of sources of evidence

The search was executed in May 2022. Studies were collated and screened for duplicates in EndNote X9 by one reviewer (KC). Eligible studies were imported into Covidence, a web-based platform that streamlines the process of systematic and other literature reviews [14], during which a further 125 duplicate records were excluded (total of 5230 duplicate studies). Paired independent review of 100% of studies at title and abstract was undertaken (KC, ET), as well as at full text level (KC, ET), reasons for exclusions were noted, and the eligible studies moved forward for data extraction.

**Table 1:** MEDLINE search

Population	Concept	Context
(not specified)	1. (malposit* or unplan* remov* or early remov* or infilt* or thrombot* or skin irrit* or skin impair* or migrat* or obstruct* or block* or occlud* or occlus* or remov* or premature remov*).mp	13. Central Venous Catheters/ae or Catheters, Indwelling/ae
	2. (safety or injur* or complication* or failure* or rupture* or damage* or dislodge* or unplanned or early).ti,kw.	14. Catheterization, Central Venous/ae
	3. (Catheter-Related Infections/ or Catheter Obstruction/) not urinary.mp.	15. ((catheter* or port* or access device* or central line) adj3 venous).mp.
	4. *Accidental injuries/ or *death/	16. ((peripheral* inserted central catheter* or percutaneous inserted central catheter* or infusaport or TIVAD or IVP or CVAD or PICC or CICC or hickman or central venous access device*) not urinary).mp.
	5. venous thrombosis/ or thrombophlebitis/ or upper extremity deep vein thrombosis/	17. ((cuff or tunnell* or implanted or implantable) adj3 (venous or access)).mp
	6. Phlebitis/	18. 13 or 14 or 15 or 16 or 17
	7. exp “Extravasation of diagnostic and therapeutic materials”/	
	8. exp Dermatitis, Contact/	
	9. Equipment Failure Analysis/	
	10. *Equipment Failure/	
	11. *Foreign-Body Migration/	
	12. device removal/	
	13. 1 or 2 or 3 or 4 or 5 or 6 or 7 or 8 or 9 or 10 or 11 or 12	

/ = subject heading, *exp *Explode; *before subject = focused subject heading (major topic); *after word = word is truncated to identify word variations e.g. remov*= removal, removes or removed; adj3 = search for (first group of word/s) within 3 words of (second group of word/s) .mp = title, abstract, original title, author keywords, name of substance word, subject heading word, protocol supplementary concept word, rare disease supplementary concept word, organism Supplementary Concept Word, Population Supplementary Concept Word, unique identifier, synonyms, floating sub-heading word; and .ti,kw. = words in title or author keywords; *ae* Adverse events

### Data charting process

Data were extracted in Covidence using an a priori template established for this review by one author (KC). Data included key study (i.e., year, title, authors, country where the study took place, study design, aims and objectives, and participant details including number and diagnoses) and device (i.e., CVAD terminologies and abbreviations, terminologies used to describe CVAD complications and definitional sources, and terminologies used to describe CVAD removal reasons and definitional sources) details. Form fields were primarily free text to accurately capture the nuances in terminologies and definitional sources for premature removals and complications.

The data charting process was undertaken independently by two authors for 20% of the studies (KC, ET). Any conflicts were discussed and resolved between the two reviewers. Level of agreement was high so individual data extraction was completed for the remainder of the studies (KC).

### Synthesis of results

Study data were stratified according to whether only one or multiple reasons for premature removal, or only one or multiple complications were reported. Data from studies reporting complications that did not indicate whether the complication resulted in premature removal were reported separately.

Definitional sources for complications and removal reasons were categorised as follows: national resources or guidelines (e.g., Centers for Disease Control and Prevention-National Healthcare Safety Network (CDC-NHSN), Infectious Diseases Society of America (IDSA) guidelines), other published studies, author-derived, or a combination of the first three categories. Descriptive statistics, primarily counts and percentages, tables and bar graphs were used to summarise charted data.

## Results

### Selection of sources of evidence

**Fig. 1:**
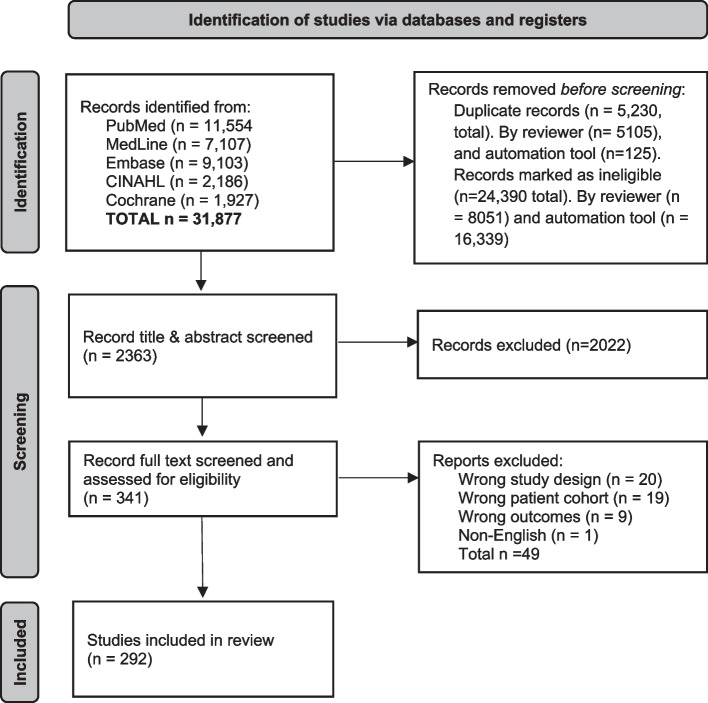
PRISMA flow diagram

The search identified 31,877 records. After removing duplicates (*n* = 5230) and irrelevant studies (*n* = 24,390) in Endnote X9, 2363 study titles and abstracts, and then 341 full texts were screened for eligibility in Covidence. A total of 292 eligible studies were identified (Fig. 1).

Central venous access device nomenclature, and taxonomy of complications and reasons for premature removal in patients with cancer: a scoping review.

### Characteristics of sources of evidence

Characteristics of the included studies are detailed in Supplement Information, Additional files 3 due to the volume of studies summarised. Of the 292 studies in this review, 193 (66%) reported on premature removal related to complications ( [2, 3, 15–205]. The remainder (*n* = 99/34%) reported on complications only [206–304] Characteristics are summarised using counts and percentages.

### Synthesis of results

Samples included patients with solid tumours only (*n* = 93), haematological malignancies and solid tumours (*n* = 92), and haematological malignancies only (*n* = 56). The remainder were described as cancer patients (*n* = 51). Studies were conducted in China, (*n* = 61), the United States of America (USA) (*n* = 41), Italy (*n* = 25), Japan and Korea (both *n* = 15), and Australia, Germany and Turkey (all *n* = 13). Twelve were multinational. According to the Joanna Briggs Institute’s levels of evidence [13], most studies were level 4 observational, descriptive studies (*n* = 174). The remainder were level 3 observational, analytical designs (*n* = 61), level 2 quasi-experimental designs (*n* = 31), level 1 experimental designs (*n* = 24) and level 5 expert opinion, bench research (*n* = 2).

### CVAD terminologies

A total of 213 unique descriptors were extracted from the included studies: 14 unique terms for CVADs, 104 for totally implantable venous access ports, 25 for peripherally inserted central catheters, 41 for tunnelled cuffed centrally inserted central catheters, 27 for centrally inserted central catheters, and two for femorally inserted central catheters. This did not include spelling variations, hyphenation, or use of capitals, or the use of multiple different terms for the device in the same study. The greatest variation was related to the descriptive nature of the names. For example, for totally implantable venous access ports the descriptors included combinations of totally or fully, subcutaneously or tunnelled, implanted or implantable; chest, arm, subclavian, internal jugular, brachial, groin or centrally inserted; devices, catheters, ports or systems; central venous, vascular or venous access; single or dual chamber; chemotherapy or infusion; traditional or power-injectable; PICC, peripherally inserted or peripheral central ports; variations on port, portacath, portacath and the various trade names.

### Premature CVAD removal related to complications

Of the 193 studies that reported on premature removals, 128 (66%) identified multiple types of complications including catheter occlusion, malposition, dislodgement, fracture, local bleeding, infection, or skin necrosis. The remainder (*n* = 65, 34%) identified one complication only, most commonly infection (*n* = 18) or thrombosis (*n* = 14).

**Fig. 2:**
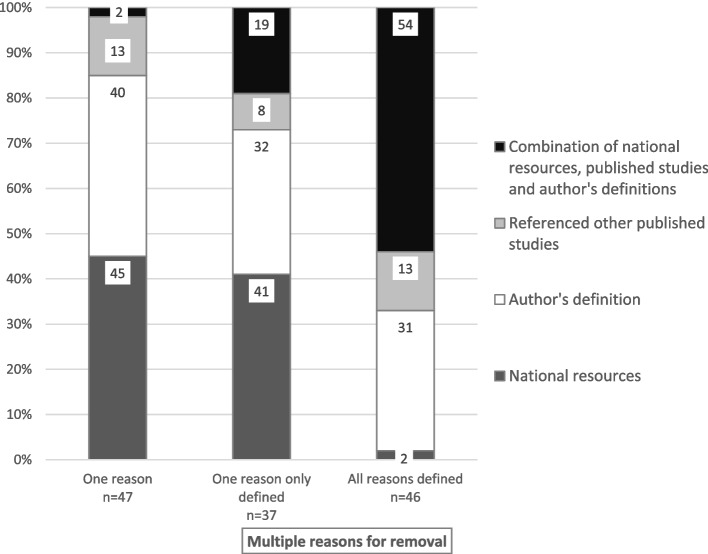
Definitional sources for premature CVA removal where provided

In studies reporting on multiple reasons for premature removal, definitional sources were not provided in 45 (35%) studies, for one reason only in 37 (29%) studies, and for all reasons in 46 (36%) studies. In studies that reported one premature removal reason only, the definition was provided in 47 (72%) studies, and not provided in 18 (28%) studies. The definitional sources in these studies included local national resources or guidelines in 21 (45%) studies, author-derived definitions in 19 (40%), definitions from other published studies in six (13%) and a combination of these sources in one (2%) study. The definitional sources in studies with multiple reasons for removal included a combination of national guidelines or resources, definitions from other published studies or author-derived definitions (Fig. 2).

### CVAD complications

Of the 99 studies that reported CVAD-related complications, 49 (49%) reported one complication and 50 (51%) reported on multiple complications. Complication definitions were provided in 36 (73%) studies reporting one complication, and no definitions provided in 13 (27%). For studies that reported on multiple complications, all complications were defined in 20 (40%) studies, only one and not all complications in 14 (28%) studies, and no complication definitions were provided in 16 (32%) studies.

**Fig. 3:**
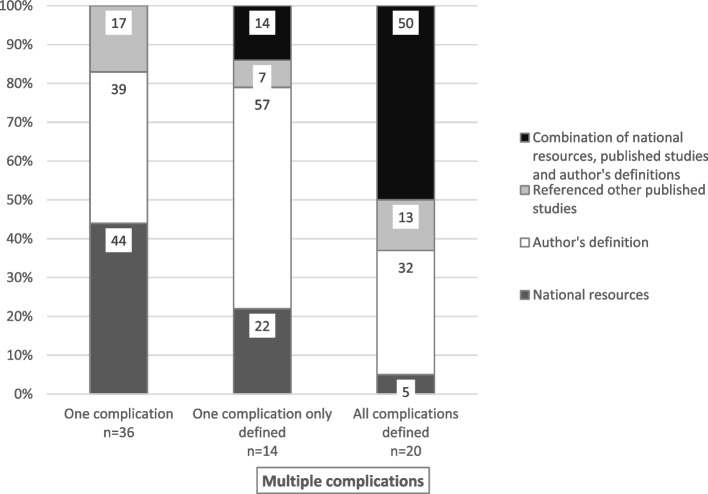
Definitional sources for CVAD complications where provided

Definitional sources in studies that reported one type of complication were from national resources or guidelines in 16 (44%) studies (e.g., CDC-NHSN or IDSA), author-derived in 14 (39%), and from other published studies in six (17%) studies (Fig. 3). Comparatively, of the studies that reported on multiple complications, fewer referenced national resources (*n* = 2, 10%); more were author-derived (*n* = 10, 50%) or used a combination of sources (*n* = 8, 40%) when all complications were defined. Definitional sources were from national resources in three [21] studies, author-derived in eight (57%) studies, other published studies in one (7%) and a combination of sources in two (14%) studies that defined only one of the multiple complications.

## Discussion

This review identified considerable variation in CVAD terminology related to reason for removal and the actual device itself. This included over 200 unique names for the different types of CVADs, with the greatest variation evident for *totally implantable venous access devices* or *ports* with over 100 unique names. In addition to inconsistency with definitions and device terminology between studies, inconsistencies were also observed *within* the same study, underscoring the complexity and confusion in this clinical issue.

Terminologies were used interchangeably such as *central venous catheters* (CVC) and *central venous access devices*. CVC was also used to describe the multi-lumen catheter most commonly used in critical care units. Despite the term *central venous catheter* being used more frequently as the term to describe all types of devices, it does not accurately describe or reflect the wide variety of implanted, cuffed or tunnelled catheters and devices, or contemporary innovations in insertion techniques; for example, tunnelling PICCs. The term *central venous access device* is more inclusive, intuitive, and reflective of the diversity in contemporary clinical practice [305].

Similar findings have previously been reported in other research. In a Delphi consensus study about a minimum dataset for vascular access, no standardised CVADs terms were identified [306]. The authors advocated for development of a vascular access minimum dataset to overcome lack of clarity in the literature that hampers robust data collection, analysis and interoperability within and across countries, ultimately adversely affecting patient outcomes [6, 306]. In response to their findings, Schults et al. (2020) subsequently developed a common set of descriptors (nomenclature) for commonly used vascular access devices [306]. However, these descriptors did not include CVADs commonly used in cancer care (e.g., tunnelled cuffed centrally inserted central catheters, apheresis catheters), and contemporary insertion techniques (e.g. tunnelled peripherally inserted central catheters). A more comprehensive set of descriptors need to be developed to represent CVADs used in cancer care.

Considerable variation in CVAD nomenclature evident in this review is problematic. A lack of standardised nomenclature impairs communication and interoperability between healthcare professionals and organisations locally and globally, and fractures data sharing, linkage, analysis and the evidence base from clinical practice [6, 306]. The World Health Organization states that standardised nomenclature is essential for recording and surveillance of all types of medical devices including CVADs [307], and in the systematic review of 20 papers by Gildow and Lazar (2022), standardised nomenclature was shown to be associated with reduced clinical errors and patient injury, improved communication and opportunity for standardisation of clinical care [308].

Most studies reported multiple reasons for premature device removal as opposed to a single reason for removal. Research investigating multiple reasons for removal reflects the increasing complexity of care and treatment for people with cancer, the majority of whom require CVAD support. The multiplicity of treatment and supporting therapies that commonly characterise care for a person with cancer, compounded by patient, clinician, therapy, and workplace related factors, come together to compound risk of premature CVAD removal. The interplay between one or more of these factors increases the risk of premature removal increasing morbidity and mortality, and cost of care [4, 309–311].

The only consistently defined premature removal reason was infection. Nearly all studies cited national sources for catheter-related blood stream infection (CRBSI) or the surveillance definition for central line-associated blood stream infection (CLABSI), with the majority citing CDC [312] or IDSA [313] from the USA. There was no consistency in definitions for any other reason for premature removal. This is an important finding with overt implications for quality and safety of care. Heterogeneity of terminology and definitions impair standardised clinical management by causing confusion and permitting an inconsistent approach for the different members of the healthcare team and clinical specialties, and consequently negatively impacts quality and safety of patients [314]. Standardised nomenclature, clinical procedures and standardisation of care have been shown to reduce errors and patient injury by improving communication and dissemination of evidence to inform clinical practices [308].

The infinite potential for utilising routinely collected patient management data and outcomes captured in EHR systems for clinical research into improving patient care and outcomes [315] cannot be realised when such variation exists. Consistency in EHR data is key to the efficient and effective collation and linkage of data required for the development of a reliable big data set [308, 315]. Clinical data, expertise and knowledge integrated with current evidence are the cornerstones of a learning health system which aims to provide informed, safer, higher quality clinical care [8]. Also, consistent data and definitions are required for meta-analyses in quantitative research [218].

Standardised nomenclature in healthcare is complex requiring a multifaceted response. Strategies require collaboration, consensus, communication, and implementation by multidisciplinary professionals including clinicians, health economists, and health service researchers, strategists, and implementation science professionals. This includes commitment by journals, national peak bodies and associations to use the standardised nomenclature as consistency at a system level is required to provide the guidance for the end users. Furthermore, regular review of nomenclature is required so it accurately reflects contemporary evidence in the literature, clinical practice, emerging technology and products.

As EHRs become increasingly prevalent across health services, they offer opportunity for standardisation of clinical nomenclature. For example, different standardised global clinical languages such as SNOMED CT or International Classification of Diseases 10^th^ Revision are translatable and already have equivalent codes for use in EHRs. Leveraging the opportunity of EHRs will require close collaboration between EHR development teams and all end users of the EHR systems.

### Limitations

There are a number of limitations of this scoping review. Limiting the patient cohort to patients with cancer may restrict the applicability to other patient cohorts. However, this was considered to have minimal impact as CVADs are used across multiple patient cohorts. The date range was five years after the 2016 edition of the Infusion Therapy Standards of Practice [12], so all descriptors and definitions may not be captured; however, it reflects contemporary practice, policy and research. The volume of studies did not allow for analysis beyond the absolute numbers of the different types of CVADs and categories of resources for definitions of CVAD complications and reasons for removal. Establishing consistent definitions for each type of premature removal or complication was not possible. The exclusion of non-English studies is important to acknowledge as a limitation when considering the results and findings of this review.

## Conclusions

Standardised CVAD nomenclature and definitions for premature CVAD removal and complications do not exist. This impacts effective and accurate communication and has been shown to hamper safe, effective cancer care. It also prevents interoperability between individuals and organisations globally to inform research to reduce the incidence and impact of CVAD complications and premature removal on cancer and patients’ experience of care, health outcomes and health system costs. Collaboration, consensus, and standardisation is required to deliver quality CVAD care.

### Supplementary information

**Additional file 1.** Scoping review protocol.

**Additional file 2.** Search strategy.

**Additional file 3.** Included studies.

**Additional file 4.** Summary of CVAD terminology.

## Data Availability

All data generated or analysed during this study are included in this published article [and its supplementary information files].
