# Supplementary material for: Central venous access device terminologies, complications, and reason for removal in oncology: a scoping review
Source: BMC Cancer. 2024 Apr 19;24:498. doi: 10.1186/s12885-024-12099-8 (PMC11027380; doi:10.1186/s12885-024-12099-8)
Supplement: Supplementary file 1 — Additional file 1. Scoping review protocol. [file 12885_2024_12099_MOESM1_ESM.docx]

**Additional file 1: Scoping review protocol**

**Title**

Central venous access device terminologies, complications, and reason for removal in oncology: A scoping review.

**Objectives**

The objective of this scoping review is to describe central venous access device (CVAD) terminology, complications, and reasons for removal in published literature for patients with haematological malignancies.

**Questions**

This review aims to answer four questions:

1. Is CVAD terminology consistent?
2. How are CVAD names abbreviated?
3. Are CVAD complication and premature CVAD removal definitions homogenous?
4. What are definitional sources for complications and premature removal?

**Inclusion criteria**

*Patient cohort*:

- patients with haematology and solid malignancies

*CVADs*: all types of CVADs oncology patients may require range of CVAD types over trajectory of prescribed therapy including:

- Centrally inserted central catheter (CICC)
- Tunnelled cuffed-centrally inserted central catheter (tc-CICC)
- Totally Implantable Venous Access Device – chest or arm (TIVAD)
- Peripherally Inserted Central Catheter (PICC)
- Apheresis CICCs (A-CICC) and tc-A-CICCs
- Haemodialysis CICCs (H-CICC) and tc-H-CICC

*Patient age****:***

- adult ≥ 18 years
- adult in mixed patient population studies (paediatrics and adults) if data are separate

*Study design*

- systematic reviews, meta-analyses, experimental (randomised/non-randomised), observational, clinical / quality improvement, literature reviews, survey/questionnaire.

*Date:*

- 2017 to 2022

Language:

- English

***Exclusions***

*CVADs*:

- arterial
- intraosseous
- umbilical catheters
- insertion related – intracavity electrocardiogram, ultrasound, seldinger, landmark, puncture, fluoroscopy, bedside

*Patient age:*

- adolescent
- paediatric
- neonates (less than 28 days old)

*Study designs:*

- study protocols
- qualitative studies
- conference abstracts
- letters to editor
- education articles
- posters
- case studies

**Rationale for exclusions:**

- arterial catheters are used primarily in intensive care settings
- intraosseous route is typically used in emergent situations and not with patients with haematological malignancies
- umbilical catheters are used in neonates (excluded population)
- insertion is considered 1% of the life of a device and the maintenance phase and removal is considered 99%, so the focus of this review
- adult was to simplify the patient cohort and reduce the excessive search results

**Context**

Patients with cancer require CVADs for administration of prescribed and supportive therapies. CVADs are an invasive device with inherent risks for complications and premature removal in this patient cohort. CVADs inserted are influenced by patient, treatment, clinical and workplace factors. Hence considerable variation exists between organisations which is reflected in the published literature.

**Search Strategy**

- Two groups of search terms: CVAD terminologies, complications/premature removal reasons
- Contemporary literature, post Infusion Therapy Standards, 2016
- Inclusion of five databases: Medline, PubMed, Embase, Cinahl Complete, Cochrane Library
- Collaborate with medical librarian to refine search strategy

**Data extraction & display of results**

- Data extraction tool in Covidence
- Fields include study (authors, year, country, study design, number of study participants, aims/objectives, patient cohort) and CVAD characteristics (type, names used by authors, abbreviations used, complication name, complication abbreviation, complication definition, premature removal name, abbreviation and definition)
- Free text options to capture nuances
- Export into Excel for analysis
- Use of tables, graphs as appropriate
