# Supplementary material for: Central venous access device terminologies, complications, and reason for removal in oncology: a scoping review
Source: BMC Cancer. 2024 Apr 19;24:498. doi: 10.1186/s12885-024-12099-8 (PMC11027380; doi:10.1186/s12885-024-12099-8)
Supplement: Supplementary file 2 — Additional file 2. Search strategy. [file 12885_2024_12099_MOESM2_ESM.docx]

Search strategy

**MedLine**

| 1 | Central Venous Catheters/ae or Catheters, Indwelling/ae | 8266 |
| --- | --- | --- |
| 2 | Catheterization, Central Venous/ae | 8974 |
| 3 | ((catheter* or port* or access device* or central line) adj3 venous).mp. | 36371 |
| 4 | ((peripheral* inserted central catheter* or percutaneous inserted central catheter* or infusaport or TIVAD or IVP or CVAD or PICC or CICC or hickman or central venous access device*) not urinary).mp. | 5477 |
| 5 | ((cuff or tunnel* or implanted or implantable) adj3 (venous or access)).mp. | 2384 |
| 6 | 1 or 2 or 3 or 4 or 5 | 44928 |
| 7 | device removal/ | 13849 |
| 8 | *Foreign-Body Migration/ | 7069 |
| 9 | *Equipment Failure/ | 3962 |
| 10 | Equipment Failure Analysis/ | 36963 |
| 11 | exp Dermatitis, Contact/ | 34448 |
| 12 | (malposit* or unplan* remov* or early remov* or infilt* or thrombot* or skin irrit* or skin impair* or migrat* or dislodg* or obstruct* or block* or occlud* or occlus* or remov* or premature remov*).mp. | 2648762 |
| 13 | (safety or injur* or complication* or failure* or rupture* or damage* or dislodge* or unplanned or early).ti,kw. | 1259659 |
| 14 | (Catheter-Related Infections/ or Catheter Obstruction/) not urinary.mp. | 4400 |
| 15 | *Accidental injuries/ or *death/ | 12333 |
| 16 | venous thrombosis/ or thrombophlebitis/ or upper extremity deep vein thrombosis/ | 47830 |
| 17 | Phlebitis/ | 3718 |
| 18 | exp "Extravasation of diagnostic and therapeutic materials"/ | 3702 |
| 19 | 7 or 8 or 9 or 10 or 11 or 12 or 13 or 14 or 15 or 16 or 17 or 18 | 3889852 |
| 20 | 6 and 19 | 17930 |
| 21 | limit 20 to (english language and yr="2011 -Current") | 7366 |
| 22 | 21 not ((animals/ not humans/) or (animal or mice or mouse or rat or rats or porcine or pig or pigs or sheep or murine or canine or rabbit* or in vitro).ti.) | 7101 |

**Embase**

| 1 | central venous catheter/ or nontunneled central venous catheter/ or peripherally inserted central venous catheter/ or subclavian vein catheter/ or tunneled central venous catheter/ or indwelling catheter/ | 36569 |  |
| --- | --- | --- | --- |
| 2 | central venous catheterization/ or subclavian vein catheterization/ | 9423 |  |
| 3 | ((catheter* or port* or access device* or central line) adj3 venous).tw,kw. | 40648 |  |
| 4 | ((peripheral* inserted central catheter* or percutaneous inserted central catheter* or infusaport or TIVAD or IVP or CVAD or PICC or CICC or hickman or central venous access device*) not urinary).tw,kw. | 9539 |  |
| 5 | ((cuff or tunnel* or implanted or implantable) adj3 (venous or access)).tw,kw. | 3410 |  |
| 6 | 1 or 2 or 3 or 4 or 5 | 71326 |  |
| 7 | device failure/ or device failure analysis/ | 9338 |  |
| 8 | (malposit* or unplan* remov* or early remov* or infilt* or thrombot* or skin irrit* or skin impair* or migrat* or dislodg* or obstruct* or block* or occlud* or occlus* or remov* or premature remov*).tw,kw. | 3236972 |  |
| 9 | (safety or injur* or complication* or failure* or rupture* or damage* or dislodge* or unplanned or early).ti,kw. | 1844675 |  |
| 10 | (catheter infection/ or catheter complication/ or device infection/ or catheter occlusion/ or occlusion/) not urinary.mp. | 58832 |  |
| 11 | vein thrombosis/ or deep vein thrombosis/ or jugular vein thrombosis/ or upper extremity deep vein thrombosis/ or thrombophlebitis/ or phlebitis/ | 116325 |  |
| 12 | (device removal/ or catheter removal/) not urinary.mp. | 24536 |  |
| 13 | foreign body/ | 34236 |  |
| 14 | contact dermatitis/ or contact dermatitis, irritant/ | 22563 |  |
| 15 | contrast medium extravasation/ | 3940 |  |
| 16 | accidental injury/ or death/ | 277737 |  |
| 17 | 7 or 8 or 9 or 10 or 11 or 12 or 13 or 14 or 15 or 16 | 5220358 |  |
| 18 | 6 and 17 | 31650 |  |
| 19 | limit 18 to (english language and yr="2011 -Current") | 17295 |  |
| 20 | 19 not ((animals/ not humans/) or (animal or mice or mouse or rat or rats or porcine or pig or pigs or sheep or murine or canine or rabbit* or in vitro).ti.) | 16936 |  |
| 21 | (conference abstract or conference paper or "conference review" or editorial or letter or press).pt. | 6905375 |  |
| 22 | 20 not 21 | 9103 |  |

**CINAHL COMPLETE**

| S18 | S16 NOT S17 (2,186) | (2,186) |
| --- | --- | --- |
| S17 | (MH "animals" NOT MH "humans") OR ((animal or mice or mouse or rat or rats or porcine or pig or pigs or sheep or murine or canine or rabbit* or in vitro)) | (341,830) |
| S16 | S15 | (2,236) |
| S15 | S13 AND S14 | (4,005) |
| S14 | S5 OR S6 OR S7 OR S8 OR S9 OR S10 OR S11 OR S12 | (531,910) |
| S13 | S1 OR S2 OR S3 OR S4 | (10,720) |
| S12 | TI safety or injur* or complication* or failure* or rupture* or damage* or dislodge* or unplanned or early | (365,599) |
| S11 | (MH "Phlebitis+") OR (MH "Thrombophlebitis+") OR (MH "Death+") OR (MH "Extravasation of Diagnostic and Therapeutic Materials") OR (MH "Accidental Injuries") | (59,531) |
| S10 | ( ( (MH "Catheter-Related Infections+") OR (MH "Catheter-Related Bloodstream Infections") OR (MH "Catheter Occlusion") ) ) NOT (TI urinary OR AB urinary) | (6,412) |
| S9 | TI (malposit* or unplan* remov* or early remov* or infilt* or thrombot* or skin irrit* or skin impair* or migrat* or dislodg* or obstruct* or block* or occlud* or occlus* or remov* or premature remov* OR AB malposit* or unplan* remov* or early remov* or infilt* or thrombot* or skin irrit* or skin impair* or migrat* or dislodg* or obstruct* or block* or occlud* or occlus* or remov* or premature remov*) | (88,752) |
| S8 | (MH "Dermatitis, Contact+") | (3,936) |
| S7 | (MH "Equipment Failure+") | (21,472) |
| S6 | (MH "Foreign-Body Migration") | (2,173) |
| S5 | (MH "Device Removal+") | (5,793) |
| S4 | TI ((cuff or tunnel* or implanted or implantable) N3 (venous or access) ) OR AB ( (cuff or tunnel* or implanted or implantable) N3 (venous or access)) | (698) |
| S3 | TI (((peripheral* inserted central catheter* or percutaneous inserted central catheter* or infusaport or TIVAD or IVP or CVAD or PICC or CICC or hickman or central venous access device*) not urinary) ) OR AB ( ((peripheral* inserted central catheter* or percutaneous inserted central catheter* or infusaport or TIVAD or IVP or CVAD or PICC or CICC or hickman or central venous access device*) not urinary)) | (2,498) |
| S2 | TI ((catheter* or port* or access device* or central line) N3 venous) OR AB ((catheter* or port* or access device* or central line) N3 venous) | (7,863) |
| S1 | (MH "Central Venous Catheters"/AE) OR (MH "Peripherally Inserted Central Catheters/AE") OR (MH "Catheterization, Peripheral Central Venous"/AE) OR (MH "Catheterization, Central Venous/AE") OR (MH "Catheter Care, Peripherally Inserted Central/AE") | (2,402) |

**COCHRANE**

| **Search Name:** | **central venous catheter complications Kerrie Curtis** | **Column1** |
| --- | --- | --- |
| Date Run: | 13/04/2021 |  |
| Comment: |  |  |
|  |  |  |
| ID | Search | Hits |
| #1 | MeSH descriptor: [Central Venous Catheters] explode all trees and with qualifier(s): [adverse effects - AE] | 60 |
| #2 | MeSH descriptor: [Catheterization, Central Venous] explode all trees and with qualifier(s): [adverse effects - AE] | 463 |
| #3 | ((peripheral* inserted central catheter* OR percutaneous inserted central catheter* OR infusaport OR TIVAD OR IVP OR CVAD OR PICC OR CICC OR hickman OR central venous access device*) NOT urinary):ti,ab,kw (Word variations have been searched) | 1172 |
| #4 | ((cuff OR tunnel* OR implanted OR implantable) NEAR/3 (venous OR access)):ti,ab,kw | 295 |
| #5 | ((catheter* OR port* OR access device* OR central line) NEAR/3 venous):ti,ab,kw | 6111 |
| #6 | #1 OR #2 OR #3 OR #4 OR #5 | 6600 |
| #7 | MeSH descriptor: [Device Removal] explode all trees | 426 |
| #8 | MeSH descriptor: [Foreign-Body Migration] this term only | 114 |
| #9 | MeSH descriptor: [Equipment Failure] this term only | 673 |
| #10 | MeSH descriptor: [Catheter-Related Infections] explode all trees | 313 |
| #11 | MeSH descriptor: [Catheter Obstruction] this term only | 25 |
| #12 | (urinary):ti,ab,kw | 45755 |
| #13 | (#10 OR #11) NOT #12 | 279 |
| #14 | MeSH descriptor: [Accidental Injuries] this term only | 2 |
| #15 | MeSH descriptor: [Death] this term only | 201 |
| #16 | MeSH descriptor: [Venous Thrombosis] this term only | 1267 |
| #17 | MeSH descriptor: [Thrombophlebitis] this term only | 1060 |
| #18 | MeSH descriptor: [Upper Extremity Deep Vein Thrombosis] this term only | 23 |
| #19 | MeSH descriptor: [Phlebitis] this term only | 180 |
| #20 | MeSH descriptor: [Extravasation of Diagnostic and Therapeutic Materials] explode all trees | 96 |
| #21 | (malposit* OR unplan* remov* OR early remov* OR infilt* OR thrombot* OR skin irrit* OR skin impair* OR migrat* OR dislodg* OR obstruct* OR block* OR occlud* OR occlus* OR remov* OR premature remov*):ti,ab,kw | 184429 |
| #22 | (safety OR injur* OR complication* OR failure* OR rupture* OR damage* OR dislodge* OR unplanned OR early):ti | 164490 |
| #23 | (safety OR injur* OR complication* OR failure* OR rupture* OR damage* OR dislodge* OR unplanned OR early):kw | 268950 |
| #24 | #7 OR #8 or #9 or #13 OR #14 or #15 or #16 or #17 or #18 or #19 or #20 or #21 or #22 or #23 | 507565 |
| #25 | #6 AND #24 with Publication Year from 2011 to 2021, in Trials | 1927 |

PubMED

| #20 | Search: **(#18) NOT (#19)** | [11,554](https://pubmed.ncbi.nlm.nih.gov/?term=%28%2318%29+NOT+%28%2319%29&sort=&size=200) |
| --- | --- | --- |
| #19 | Search: **(animals/ not humans/) or (animal or mice or mouse or rat or rats or porcine or pig or pigs or sheep or murine or canine or rabbit* or in vitro)** | [8,067,767](https://pubmed.ncbi.nlm.nih.gov/?term=%28animals%2F+not+humans%2F%29+or+%28animal+or+mice+or+mouse+or+rat+or+rats+or+porcine+or+pig+or+pigs+or+sheep+or+murine+or+canine+or+rabbit%2A+or+in+vitro%29&sort=&size=200) |
| #18 | Search: **(#17) AND (english[la])** | [12,347](https://pubmed.ncbi.nlm.nih.gov/?term=%28%2317%29+AND+%28english%5Bla%5D%29&sort=&size=200) |
|  | Search: **(#16) AND** 2017/01/01:3000/12/31[Date - Entry] | 5,031 |
| #17 | Search: **(#16) AND** 2011/01/01:2016/12/31[Date - Entry] | [6,523](https://pubmed.ncbi.nlm.nih.gov/?term=%28%2316%29+AND+%282011%2F01%2F01%3A2021%2F01%2F01%5Bdp%5D%29&sort=&size=200) |
| #16 | Search: **(#8) AND (#15)** | [30,549](https://pubmed.ncbi.nlm.nih.gov/?term=%28%238%29+AND+%28%2315%29&sort=&size=200) |
| #15 | Search: **(#9) OR (#10) OR (#11) OR (#12) OR (#13) OR (#14)** | [4,013,568](https://pubmed.ncbi.nlm.nih.gov/?term=%28%239%29+OR+%28%2310%29+OR+%28%2311%29+OR+%28%2312%29+OR+%28%2313%29+OR+%28%2314%29&sort=&size=200) |
| #14 | Search: **"extravasation of diagnostic and therapeutic materials"[MeSH Terms]** | [3,702](https://pubmed.ncbi.nlm.nih.gov/?term=%22extravasation+of+diagnostic+and+therapeutic+materials%22%5BMeSH+Terms%5D&sort=&size=200) |
| #13 | Search: **("catheter obstruction"[MeSH Terms]) NOT (URINARY)** | [264](https://pubmed.ncbi.nlm.nih.gov/?term=%28%22catheter+obstruction%22%5BMeSH+Terms%5D%29+NOT+%28URINARY%29&sort=&size=200) |
| #12 | Search: **("catheter related infections"[MeSH Terms]) NOT (URINARY)** | [4,205](https://pubmed.ncbi.nlm.nih.gov/?term=%28%22catheter+related+infections%22%5BMeSH+Terms%5D%29+NOT+%28URINARY%29&sort=&size=200) |
| #11 | Search: **safety[Title] OR injur*[Title] OR complication*[Title] OR failure*[Title] OR rupture*[Title] OR damage*[Title] OR dislodge*[Title] OR unplanned[Title] OR early[Title]** | [1,232,900](https://pubmed.ncbi.nlm.nih.gov/?term=safety%5BTitle%5D+OR+injur%2A%5BTitle%5D+OR+complication%2A%5BTitle%5D+OR+failure%2A%5BTitle%5D+OR+rupture%2A%5BTitle%5D+OR+damage%2A%5BTitle%5D+OR+dislodge%2A%5BTitle%5D+OR+unplanned%5BTitle%5D+OR+early%5BTitle%5D&sort=&size=200) |
| #10 | Search: **malposit*[Text Word] OR unplan* remov*[Text Word] OR early remov*[Text Word] OR infilt*[Text Word] OR thrombot*[Text Word] OR skin irrit*[Text Word] OR skin impair*[Text Word] OR migrat*[Text Word] OR dislodg*[Text Word] OR obstruct*[Text Word] OR block*[Text Word] OR occlud*[Text Word] OR occlus*[Text Word] OR remov*[Text Word] OR premature remov*[Text Word]** | [2,645,279](https://pubmed.ncbi.nlm.nih.gov/?term=malposit%2A%5BText+Word%5D+OR+unplan%2A+remov%2A%5BText+Word%5D+OR+early+remov%2A%5BText+Word%5D+OR+infilt%2A%5BText+Word%5D+OR+thrombot%2A%5BText+Word%5D+OR+skin+irrit%2A%5BText+Word%5D+OR+skin+impair%2A%5BText+Word%5D+OR+migrat%2A%5BText+Word%5D+OR+dislodg%2A%5BText+Word%5D+OR+obstruct%2A%5BText+Word%5D+OR+block%2A%5BText+Word%5D+OR+occlud%2A%5BText+Word%5D+OR+occlus%2A%5BText+Word%5D+OR+remov%2A%5BText+Word%5D+OR+premature+remov%2A%5BText+Word%5D&sort=&size=200) |
| #9 | Search: **(((((((((("device removal"[MeSH Terms]) OR (foreign body migration[MeSH Terms])) OR (equipment failure[MeSH Terms])) OR ("equipment failure analysis"[MeSH Terms])) OR ("dermatitis, contact"[MeSH Terms])) OR (phlebitis[MeSH Terms])) OR (accidental injuries[MeSH Terms])) OR (death[MeSH Terms])) OR (venous thrombosis[MeSH Terms])) OR (thrombophlebitis[MeSH Terms])) OR (upper extremity deep vein thrombosis[MeSH Terms])** | [349,186](https://pubmed.ncbi.nlm.nih.gov/?term=%28%28%28%28%28%28%28%28%28%28%22device+removal%22%5BMeSH+Terms%5D%29+OR+%28foreign+body+migration%5BMeSH+Terms%5D%29%29+OR+%28equipment+failure%5BMeSH+Terms%5D%29%29+OR+%28%22equipment+failure+analysis%22%5BMeSH+Terms%5D%29%29+OR+%28%22dermatitis%2C+contact%22%5BMeSH+Terms%5D%29%29+OR+%28phlebitis%5BMeSH+Terms%5D%29%29+OR+%28accidental+injuries%5BMeSH+Terms%5D%29%29+OR+%28death%5BMeSH+Terms%5D%29%29+OR+%28venous+thrombosis%5BMeSH+Terms%5D%29%29+OR+%28thrombophlebitis%5BMeSH+Terms%5D%29%29+OR+%28upper+extremity+deep+vein+thrombosis%5BMeSH+Terms%5D%29&sort=&size=200) |
| #8 | Search: **(#1) OR (#3) OR (#5) OR (#6) OR (#7)** | [77,150](https://pubmed.ncbi.nlm.nih.gov/?term=%28%231%29+OR+%28%233%29+OR+%28%235%29+OR+%28%236%29+OR+%28%237%29&sort=&size=200) |
| #7 | Search: **(cuff[Text Word] OR cuffed[Text Word] OR tunnel*[Text Word] OR implanted[Text Word] OR implantable[Text Word]) AND (venous[Text Word] OR access[Text Word])** | [11,710](https://pubmed.ncbi.nlm.nih.gov/?term=%28cuff%5BText+Word%5D+OR+cuffed%5BText+Word%5D+OR+tunnel%2A%5BText+Word%5D+OR+implanted%5BText+Word%5D+OR+implantable%5BText+Word%5D%29+AND+%28venous%5BText+Word%5D+OR+access%5BText+Word%5D%29&sort=&size=200) |
| #6 | Search: **(peripheral* inserted central catheter* or percutaneous inserted central catheter* or "infusaport" or "infusaports" or "TIVAD" or "TIVADs" or "IVP"or "IVPs" or "CVAD" or "CVADs" or "PICC" or "PICCs" or "CICC"or "CICCs" or "CVC" or "CVCs" or "Hickman" or "Hickmans" or central venous access device*) NOT (urinary)** | [16,470](https://pubmed.ncbi.nlm.nih.gov/?term=%28peripheral%2A+inserted+central+catheter%2A+or+percutaneous+inserted+central+catheter%2A+or+%22infusaport%22+or+%22infusaports%22+or+%22TIVAD%22+or+%22TIVADs%22+or+%22IVP%22or+%22IVPs%22+or+%22CVAD%22+or+%22CVADs%22+or+%22PICC%22+or+%22PICCs%22+or+%22CICC%22or+%22CICCs%22+or+%22CVC%22+or+%22CVCs%22+or+%22Hickman%22+or+%22Hickmans%22+or+central+venous+access+device%2A%29+NOT+%28urinary%29&sort=&size=200) |
| #5 | Search: **(catheter*[Text Word] OR port*[Text Word] OR access device*[Text Word] OR central line[Text Word])AND (venous[Text Word])** | [61,098](https://pubmed.ncbi.nlm.nih.gov/?term=%28catheter%2A%5BText+Word%5D+OR+port%2A%5BText+Word%5D+OR+access+device%2A%5BText+Word%5D+OR+central+line%5BText+Word%5D%29AND+%28venous%5BText+Word%5D%29&sort=&size=200) |
| #3 | Search: **catheterization, central venous/adverse effects[MeSH Terms]** | [8,974](https://pubmed.ncbi.nlm.nih.gov/?term=catheterization%2C+central+venous%2Fadverse+effects%5BMeSH+Terms%5D&sort=&size=200) |
| #2 | Search: **catheters, indwelling/adverse effects[MeSH Terms]** | [7,322](https://pubmed.ncbi.nlm.nih.gov/?term=catheters%2C+indwelling%2Fadverse+effects%5BMeSH+Terms%5D&sort=&size=200) |
| #1 | Search: **central venous catheters/adverse effects[MeSH Terms]** | [1,156](https://pubmed.ncbi.nlm.nih.gov/?term=central+venous+catheters%2Fadverse+effects%5BMeSH+Terms%5D&sort=&size=200) |
