# Supplementary material for: Central venous access device terminologies, complications, and reason for removal in oncology: a scoping review
Source: BMC Cancer. 2024 Apr 19;24:498. doi: 10.1186/s12885-024-12099-8 (PMC11027380; doi:10.1186/s12885-024-12099-8)
Supplement: Supplementary file 3 — Additional file 3. Included studies. [file 12885_2024_12099_MOESM3_ESM.docx]

**Supplementary Information: Additional file 3**

*Study characteristics*

| **Study ID** | **Year** | **Country** | **Study design described by authors** | **JBI - Levels of evidence for effectiveness** | **Patient cohort** |
| --- | --- | --- | --- | --- | --- |
| (Aghamohammadi, Fakhari, Ataei, Bilehjani, & Jafari, 2017) | 2017 | Iran | observational study | Level 4– Observational – Descriptive Studies | solid |
| (Agrawal et al., 2019) | 2020 | India | retrospective study | Level 4– Observational – Descriptive Studies | solid, haem |
| (Ahmad et al., 2019) | 2019 | Denmark | quality control study | Level 4– Observational – Descriptive Studies | haem |
| (Akhtar & Lee, 2021) | 2021 | Canada | chart review | Level 4– Observational – Descriptive Studies | solid, haem |
| (Alfonso Alvarez-Rodriguez, Garcia-Suarez, Fernandez-Garcia, Mendez-Martinez, & Gomez-Salgado, 2018) | 2018 | Spain | observational study | Level 4– Observational – Descriptive Studies | solid |
| (Alkindi, Chai-Adisaksopha, Cheah, & Linkins, 2018) | 2018 | Canada | chart review | Level 4– Observational – Descriptive Studies | solid, haem |
| (Ammar et al., 2019) | 2019 | Jordan | quasi-experimental pilot study | Level 2 – Quasi-experimental Designs | haem |
| (Anbar, Avci, & Cetinkaya, 2017) | 2017 | Turkey | observational study | Level 4– Observational – Descriptive Studies | solid |
| (Annetta et al., 2021) | 2021 | Italy & USA | retrospective study | Level 4– Observational – Descriptive Studies | solid |
| (Aribas et al., 2017) | 2017 | Turkey | retrospective study | Level 4– Observational – Descriptive Studies | solid |
| (Bademler, Ucuncu, Yildirim, & Karanlik, 2019) | 2019 | Turkey | retrospective study, literature review | Level 4– Observational – Descriptive Studies | solid |
| (Bai et al., 2017) | 2017 | China | retrospective study | Level 4– Observational – Descriptive Studies | solid, haem |
| (Balsorano et al., 2019) | 2020 | Italy | systematic review and meta-analysis | Level 1 – Experimental Designs | solid, haem |
| (Ban et al., 2022) | 2021 | Japan | retrospective study | Level 4– Observational – Descriptive Studies | haem |
| (Baumann Kreuziger et al., 2021) | 2021 | USA | cohort study | Level 3 – Observational – Analytic Designs Level | haem |
| (Belloni et al., 2022) | 2022 | Italy | systematic review and meta-analysis of observational studies | Level 3 – Observational – Analytic Designs Level | solid, haem |
| (Bertoglio et al., 2020) | 2020 | Italy | cohort study | Level 3 – Observational – Analytic Designs Level | solid |
| (Bertoglio et al., 2022) | 2022 | Italy | cohort study | Level 3 – Observational – Analytic Designs Level | solid |
| (Bessis et al., 2020) | 2020 | France | cohort study | Level 3 – Observational – Analytic Designs Level | solid, haem |
| (Beypinar, Demir, Uysal, Araz, & Beypinar, 2020) | 2020 | Turkey | cross-sectional study | Level 4– Observational – Descriptive Studies | solid |
| (Boll et al., 2021) | 2021 | Germany | guideline | Level 3 – Observational – Analytic Designs Level | solid, haem |
| (Bouzidi et al., 2018) | 2018 | France | retrospective study | Level 4– Observational – Descriptive Studies | solid, haem |
| (Brescia et al., 2021) | 2021 | Italy | cohort study | Level 3 – Observational – Analytic Designs Level | cancer |
| (Brito et al., 2018) | 2018 | Brazil | observational study | Level 4– Observational – Descriptive Studies | cancer |
| (Broadhurst, Moureau, & Ullman, 2017) | 2017 | Canada, Australia | scoping review | Level 4– Observational – Descriptive Studies | solid, haem |
| (Burbridge et al., 2018) | 2018 | Canada | randomized clinical trial | Level 1 – Experimental Designs | solid |
| (Jasmin D. Busch et al., 2017) | 2017 | Germany | cohort study | Level 4– Observational – Descriptive Studies | solid, haem |
| (Busch, Vens, Herrmann, Adam, & Ittrich, 2017) | 2017 | Germany | retrospective study | Level 4– Observational – Descriptive Studies | solid, haem |
| (Calò et al., 2020) | 2020 | Spain | cohort study | Level 3 – Observational – Analytic Designs Level | solid, haem |
| (Campagna, Berchialla, et al., 2019) | 2019 | Italy | retrospective study | Level 4– Observational – Descriptive Studies | solid, haem |
| (Campagna, Gonella, et al., 2019) | 2019 | Italy | retrospective study | Level 4– Observational – Descriptive Studies | solid, haem |
| (Capozzi et al., 2021) | 2021 | Italy | meta-analysis | Level 2 – Quasi-experimental Designs | solid |
| (Caris et al., 2022) | 2022 | Netherlands | cohort study | Level 3 – Observational – Analytic Designs Level | haem |
| (Carvalho Castanho et al., 2020) | 2020 | Brazil | observational study | Level 4– Observational – Descriptive Studies | haem |
| (P. Chaftari et al., 2017) | 2017 | USA | quality improvement project | Level 4– Observational – Descriptive Studies | cancer |
| (A. M. Chaftari et al., 2018) | 2018 | USA | retrospective study | Level 4– Observational – Descriptive Studies | solid, haem |
| (Chan et al., 2017) | 2017 | Australia | randomised controlled trial | Level 3 – Observational – Analytic Designs Level | solid, haem |
| (Chang et al., 2017) | 2017 | Germany | observational study | Level 4– Observational – Descriptive Studies | solid |
| (M. H. Chen, Hwang, Chang, Chiang, & Teng, 2017) | 2017 | Taiwan | cohort study | Level 3 – Observational – Analytic Designs Level | haem |
| (Y. Chen et al., 2020) | 2020 | China | cohort study | Level 3 – Observational – Analytic Designs Level | solid, haem |
| (K. Chen et al., 2021) | 2021 | Russia | comparative study | Level 3 – Observational – Analytic Designs Level | solid |
| (P. Chen, Zhu, Wan, & Qin, 2021) | 2021 | China | systematic review and meta-analysis | Level 2 – Quasi-experimental Designs | cancer |
| (X.-S. Chen, Wu, Chen, Zhang, & Liu, 2021) | 2021 | China | systematic review and meta-analysis | Level 2 – Quasi-experimental Designs | cancer |
| (Choksi, Finnegan, & Etezadi, 2020) | 2020 | USA | chart review | Level 4– Observational – Descriptive Studies | solid, haem |
| (Chong, Lai, Apisarnthanarak, & Chaiyakunapruk, 2017) | 2017 | Malaysia, Thailand, Australia, USA | Cochrane systematic review and network meta-analysis | Level 2 – Quasi-experimental Designs | solid, haem |
| (Vineet Chopra et al., 2018) | 2018 | USA | retrospective cohort study | Level 3 – Observational – Analytic Designs Level | cancer |
| (V. Chopra et al., 2022) | 2022 | USA | quasi experimental study | Level 2 – Quasi-experimental Designs | solid |
| (Chou et al., 2019) | 2019 | Taiwan | retrospective study | Level 4– Observational – Descriptive Studies | solid, haem |
| (Clari et al., 2021) | 2021 | Italy | systematic review and meta-analysis | Level 2 – Quasi-experimental Designs | solid |
| (Clatot et al., 2020) | 2020 | France | randomised study | Level 1 – Experimental Designs | solid |
| (Cornillon et al., 2017) | 2017 | France | survey | Level 4– Observational – Descriptive Studies | haem |
| (Corti et al., 2021) | 2021 | Italy | retrospective study | Level 4– Observational – Descriptive Studies | solid, haem |
| (Cotogni, Mussa, Degiorgis, De Francesco, & Pittiruti, 2021) | 2021 | Italy | cohort study | Level 3 – Observational – Analytic Designs Level | solid |
| (Cruz-Aguilar et al., 2021) | 2021 | Germany | retrospective study | Level 2 – Quasi-experimental Designs | haem |
| (Da Costa et al., 2019) | 2019 | Brazil | systematic review and meta-analysis | Level 1 – Experimental Designs | solid, haem |
| (Dai et al., 2020) | 2020 | China | randomized controlled trial | Level 2 – Quasi-experimental Designs | solid |
| (Dang, Li, & Tian, 2019) | 2019 | China | systematic review and network meta-analysis | Level 2 – Quasi-experimental Designs | cancer |
| (Davies et al., 2018) | 2018 | Canada | cohort study | Level 3 – Observational – Analytic Designs Level | solid |
| (de Campos Pereira Silveira et al., 2020) | 2020 | Canada & Brazil | systematic review and meta-analysis | Level 1 – Experimental Designs | haem |
| (Decousus et al., 2018) | 2018 | France | cohort study | Level 3 – Observational – Analytic Designs Level | solid |
| (de la Cruz-Hernandez et al., 2020) | 2020 | Mexico | observational study | Level 4– Observational – Descriptive Studies | solid, haem |
| (de Mooij et al., 2020) | 2020 | The Netherlands | observational study | Level 4– Observational – Descriptive Studies | haem |
| (Diaz et al., 2017) | 2017 | USA | phase II study | Level 4– Observational – Descriptive Studies | solid, haem |
| (Dinçer et al., 2019) | 2019 | Turkey | retrospective study | Level 4– Observational – Descriptive Studies | solid |
| (D'Souza et al., 2021) | 2021 | Oman | retrospective study | Level 4– Observational – Descriptive Studies | solid |
| (Egnatios & Gloria, 2021) | 2021 | USA | "this study" | Level 4– Observational – Descriptive Studies | solid |
| (El-Balat et al., 2018) | 2018 | Italy | "this study" | Level 4– Observational – Descriptive Studies | solid |
| (El Boghdadly et al., 2022) | 2022 | USA | poll, retrospective study | Level 4– Observational – Descriptive Studies | haem |
| (Eldeeb, Al-Asadi, & Almusarhed, 2019) | 2019 | UK | cohort study | Level 3 – Observational – Analytic Designs Level | solid |
| (Fang, Jiang, Yang, Song, & Liu, 2017) | 2017 | China | cohort study | Level 3 – Observational – Analytic Designs Level | solid |
| (Fares et al., 2019) | 2019 | USA | retrospective study | Level 4– Observational – Descriptive Studies | solid, haem |
| (Feng et al., 2021) | 2021 | China | cohort study | Level 3 – Observational – Analytic Designs Level | solid |
| (Fornaro et al., 2019) | 2019 | Italy | observational study and literature review | Level 4– Observational – Descriptive Studies | solid |
| (Fu, Lu, Wang, & Ye, 2019) | 2019 | China | not described | Level 4– Observational – Descriptive Studies | solid |
| (Furuhashi et al., 2021) | 2021 | Japan | cohort study | Level 3 – Observational – Analytic Designs Level | solid, haem |
| (GÜR, DonbaloĞLu, & GÜRkan, 2018) | 2018 | Turkey | retrospective chart review | Level 4– Observational – Descriptive Studies | solid, haem |
| (Gilardi et al., 2021) | 2021 | Italy | prospective study | Level 3 – Observational – Analytic Designs Level | solid, haem |
| (Gonzalez et al., 2021) | 2021 | Spain | cohort study | Level 3 – Observational – Analytic Designs Level | solid, haem |
| (Grau, Clarivet, Lotthe, Bommart, & Parer, 2017) | 2017 | France | cohort study | Level 3 – Observational – Analytic Designs Level | solid, haem |
| (Gray et al., 2019) | 2019 | USA | not described | Level 4– Observational – Descriptive Studies | haem |
| (Guan, Yan, Zhang, Li, & Zhou, 2022) | 2022 | China | retrospective study | Level 4– Observational – Descriptive Studies | solid, haem |
| (Gudiol et al., 2020) | 2020 | Spain | randomized controlled trial | Level 2 – Quasi-experimental Designs | haem |
| (Haggstrom, Parmar, & Brungs, 2020) | 2020 | Australia | cohort study | Level 3 – Observational – Analytic Designs Level | solid, haem |
| (Hallam, Jackson, Rajgopal, & Russell, 2018) | 2018 | UK | quality improvement program | Level 4– Observational – Descriptive Studies | solid, haem |
| (Harrold, Martin, & Bhuva, 2019) | 2019 | UK | chart audit | Level 4– Observational – Descriptive Studies | solid |
| (Yoshinori Hashimoto, Fukuta, Maruyama, Omura, & Tanaka, 2017) | 2017 | Japan | "this study" | Level 4– Observational – Descriptive Studies | haem |
| (Y. Hashimoto, Hosoda, Omura, & Tanaka, 2021) | 2021 | Japan | retrospective study | Level 4– Observational – Descriptive Studies | haem |
| (He, Ye, & Zheng, 2021) | 2021 | China | systematic review and meta-analysis of randomized controlled trials and retrospective cohort studies | Level 1 – Experimental Designs | cancer |
| (Heidenreich et al., 2020) | 2020 | Germany | retrospective study | Level 4– Observational – Descriptive Studies | haem |
| (Heidenreich et al., 2022) | 2022 | Germany | retrospective study | Level 4– Observational – Descriptive Studies | haem |
| (Hill et al., 2019) | 2019 | UK | retrospective study | Level 4– Observational – Descriptive Studies | cancer |
| (Hong et al., 2019) | 2019 | Korea | retrospective study | Level 4– Observational – Descriptive Studies | solid |
| (Hoppe et al., 2021) | 2021 | Poland | retrospective study | Level 4– Observational – Descriptive Studies | haem |
| (Htun, Ma, & Lee, 2018) | 2018 | Canada | retrospective study | Level 4– Observational – Descriptive Studies | haem |
| (V. Huang, 2018) | 2018 | USA | implementation project | Level 4– Observational – Descriptive Studies | haem |
| (W. Huang & Xu, 2020) | 2020 | China | retrospective study | Level 4– Observational – Descriptive Studies | haem |
| (C. Huang et al., 2021) | 2021 | China | randomized controlled study | Level 2 – Quasi-experimental Designs | solid, haem |
| (Huihan, Yu, Qin, & Yanping, 2018) | 2018 | China | observational study | Level 4– Observational – Descriptive Studies | solid, haem |
| (Hyo-Cheol, Saebeom, & Hoyong, 2017) | 2017 | Korea | observational study | Level 4– Observational – Descriptive Studies | solid |
| (Iftikhar et al., 2018) | 2018 | Pakistan | randomized study | Level 3 – Observational – Analytic Designs Level | haem |
| (Imaoka, Kuranishi, & Ogawa, 2018) | 2018 | Japan | retrospective Study | Level 4– Observational – Descriptive Studies | solid |
| (Ince, Ozkan, Ors, Yildirim, & Doganci, 2020) | 2020 | Turkey | observational study | Level 4– Observational – Descriptive Studies | solid, haem |
| (Inoue et al., 2020) | 2020 | Japan | retrospective study | Level 4– Observational – Descriptive Studies | solid |
| (Isom et al., 2019) | 2019 | USA | retrospective study | Level 4– Observational – Descriptive Studies | solid |
| (Jabaley et al., 2022) | 2022 | USA | clinical project | Level 4– Observational – Descriptive Studies | solid |
| (Meng Jiang, Cui, Li, Pan, & Dietrich, 2020) | 2020 | China | systematic review and meta-analysis | Level 2 – Quasi-experimental Designs | solid |
| (M. Jiang, Li, Pan, & Yu, 2020) | 2020 | China | systematic review and meta-analysis | Level 2 – Quasi-experimental Designs | solid, haem |
| (Johns, Wahlrab, & Elefritz, 2021) | 2021 | USA | cohort study | Level 3 – Observational – Analytic Designs Level | solid, haem |
| (D. Jones et al., 2017) | 2017 | UK | cohort study | Level 3 – Observational – Analytic Designs Level | solid |
| (M. Jones et al., 2021) | 2021 | Australia | cohort study | Level 3 – Observational – Analytic Designs Level | solid, haem |
| (Kagan, Salgado, Banks, Marculescu, & Cantey, 2019) | 2019 | USA | cohort study | Level 3 – Observational – Analytic Designs Level | haem |
| (Kakkos et al., 2017) | 2017 | France | observational study | Level 4– Observational – Descriptive Studies | solid |
| (J. Kang et al., 2017) | 2017 | China | cohort study | Level 3 – Observational – Analytic Designs Level | solid, haem |
| (J. R. Kang et al., 2017) | 2017 | China | cohort study | Level 3 – Observational – Analytic Designs Level | solid |
| (P. F. Kao, Weng, Tyan, Yang, & Tsao, 2017) | 2017 | Taiwan | retrospective study | Level 4– Observational – Descriptive Studies | solid, haem |
| (C.-Y. Kao et al., 2020) | 2020 | Taiwan | retrospective study | Level 4– Observational – Descriptive Studies | solid, haem |
| (Kara et al., 2021) | 2021 | Turkey | retrospective study | Level 4– Observational – Descriptive Studies | solid |
| (Karapanou et al., 2020) | 2020 | Greece | quasi experimental pre-post study | Level 2 – Quasi-experimental Designs | haem |
| (Kato et al., 2018) | 2018 | Japan | retrospective study | Level 4– Observational – Descriptive Studies | haem |
| (Kiesow, Mahnken, & Keulers, 2018) | 2018 | Germany | retrospective study | Level 4– Observational – Descriptive Studies | solid, haem |
| (Kikuchi et al., 2020) | 2020 | Japan | observational study | Level 4– Observational – Descriptive Studies | cancer |
| (J. H. Kim et al., 2018) | 2018 | Korea | retrospective study | Level 4– Observational – Descriptive Studies | solid |
| (I. J. Kim et al., 2019) | 2019 | Korea | retrospective study | Level 4– Observational – Descriptive Studies | solid, haem |
| (T. H. Kim et al., 2021) | 2021 | South Korea | retrospective study | Level 4– Observational – Descriptive Studies | haem |
| (Kinoshita et al., 2022) | 2022 | Japan | retrospective study | Level 4– Observational – Descriptive Studies | solid, haem |
| (Kitamura et al., 2020) | 2020 | Japan | retrospective study | Level 4– Observational – Descriptive Studies | haem |
| (Kleidon et al., 2021) | 2021 | Australia, USA | observational study | Level 4– Observational – Descriptive Studies | haem |
| (Koo et al., 2018) | 2018 | Australia | cohort study | Level 3 – Observational – Analytic Designs Level | solid, haem |
| (Kramer et al., 2017) | 2017 | USA | systematic review and meta-analysis | Level 1 – Experimental Designs | cancer |
| (Krumpelmann et al., 2021) | 2021 | Germany | chart review | Level 4– Observational – Descriptive Studies | solid, haem |
| (Kukla et al., 2020) | 2020 | USA | systematic literature review and meta-analysis | Level 2 – Quasi-experimental Designs | haem |
| (Kumwenda, Dougherty, Jackson, & Hill, 2021) | 2021 | UK | chart audit | Level 4– Observational – Descriptive Studies | solid, haem |
| (Lam et al., 2018) | 2018 | Canada | interrupted time series study | Level 3 – Observational – Analytic Designs Level | solid |
| (Y. M. Lee et al., 2018) | 2018 | Korea | retrospective study | Level 4– Observational – Descriptive Studies | solid, haem |
| (I. J. Lee et al., 2019) | 2019 | Korea | cohort study | Level 3 – Observational – Analytic Designs Level | solid, haem |
| (Y.-M. Lee et al., 2021) | 2021 | Republic of Korea | cohort study | Level 3 – Observational – Analytic Designs Level | cancer |
| (Li, Zhang, Ma, & Zheng, 2019) | 2019 | China | systematic review and meta-analysis | Level 1 – Experimental Designs | cancer |
| (Lichtenstein et al., 2021) | 2021 | Germany | retrospective study | Level 4– Observational – Descriptive Studies | solid, haem |
| (W. Y. Lin et al., 2017) | 2017 | Taiwan | observational study | Level 4– Observational – Descriptive Studies | solid, haem |
| (Y. Lin et al., 2020) | 2020 | China | observational study | Level 4– Observational – Descriptive Studies | solid, haem |
| (Lingegowda, Gehani, Sen, Mukhopadhyay, & Ghosh, 2020) | 2020 | India | retrospective study | Level 4– Observational – Descriptive Studies | solid, haem |
| (Liscynesky, Johnston, Haydocy, & Stevenson, 2017) | 2017 | USA | cohort study | Level 3 – Observational – Analytic Designs Level | cancer |
| (K. Liu et al., 2018) | 2018 | China | randomized controlled trial | Level 2 – Quasi-experimental Designs | solid |
| (S. Liu et al., 2019) | 2019 | China | retrospective study | Level 4– Observational – Descriptive Studies | cancer |
| (Z. Liu et al., 2019) | 2019 | PR China | "this study" | Level 4– Observational – Descriptive Studies | solid |
| (Y. Liu et al., 2020) | 2020 | China | systematic literature review and meta-analysis | Level 2 – Quasi-experimental Designs | solid |
| (B. Liu, Wu, Lin, Li, & Kuang, 2021) | 2021 | China | systematic literature review and meta-analysis | Level 1 – Experimental Designs | solid |
| (X. Liu et al., 2021) | 2021 | China | randomized controlled trial | Level 2 – Quasi-experimental Designs | solid |
| (G. D. Liu, Ma, Liu, Tang, & Tan, 2022) | 2022 | China | meta-analysis | Level 1 – Experimental Designs | cancer |
| (Longo et al., 2017) | 2017 | France | randomized trial | Level 1 – Experimental Designs | solid |
| (Lopez-Briz et al., 2018) | 2018 | Spain & UK | Cochrane intervention review | Level 2 – Quasi-experimental Designs | cancer |
| (Lo Priore, Fliedner, Heverhagen, Novak, & Marschall, 2017) | 2017 | Switzerland | surveillance study | Level 4– Observational – Descriptive Studies | solid, haem |
| (Low et al., 2020) | 2020 | Singapore | pilot study. | Level 4– Observational – Descriptive Studies | solid, haem |
| (Luong, Kroll, & Vu, 2017) | 2017 | USA | retrospective study | Level 4– Observational – Descriptive Studies | haem |
| (Y. Lv et al., 2018) | 2018 | China | systematic review and meta-analysis | Level 2 – Quasi-experimental Designs | haem |
| (L. Lv et al., 2020) | 2020 | China | observational study | Level 4– Observational – Descriptive Studies | solid |
| (Madabhavi et al., 2017) | 2017 | India | observational study | Level 4– Observational – Descriptive Studies | solid, haem |
| (Madabhavi, Patel, Anand, Kataria, et al., 2018) | 2018 | india | observational study | Level 4– Observational – Descriptive Studies | solid, haem |
| (Madabhavi, Patel, Anand, Sarkar, et al., 2018) | 2018 | India | observational study | Level 4– Observational – Descriptive Studies | solid, haem |
| (Malek & Raad, 2020) | 2020 | USA | expert review | Level 5 – Expert Opinion and Bench Research | solid |
| (Mansour et al., 2018) | 2018 | Jordan | cohort study | Level 3 – Observational – Analytic Designs Level | solid, haem |
| (Mariggio et al., 2020) | 2020 | Italy | observational study | Level 4– Observational – Descriptive Studies | haem |
| (Martinez & Capela, 2021) | 2021 | Portugal, US | comparative study | Level 1 – Experimental Designs | haem |
| (McDiarmid et al., 2017) | 2017 | Canada | cohort study | Level 4– Observational – Descriptive Studies | solid, haem |
| (McDonald et al., 2018) | 2018 | USA | retrospective study | Level 4– Observational – Descriptive Studies | haem |
| (McKeown et al., 2022) | 2022 | USA | observational study | Level 4– Observational – Descriptive Studies | haem |
| (McParlan et al., 2020) | 2020 | Northern Ireland | before and after analysis | Level 1 – Experimental Designs | solid, haem |
| (Michell, Nezami, Morris, & Hong, 2021) | 2021 | USA | retrospective study | Level 4– Observational – Descriptive Studies | haem |
| (Mielke, Wittig, & Teichgraber, 2020) | 2020 | Germany | retrospective study | Level 4– Observational – Descriptive Studies | solid |
| (Milani et al., 2017) | 2017 | Italy | cohort study | Level 3 – Observational – Analytic Designs Level | solid |
| (Mittal, Sundriyal, Naik, & Sehrawat, 2021) | 2021 | India | "this study" | Level 4– Observational – Descriptive Studies | solid, haem |
| (Mollee et al., 2020) | 2020 | Australia | randomized controlled study | Level 2 – Quasi-experimental Designs | solid, haem |
| (Moralar et al., 2021) | 2021 | Pakistan | retrospective study | Level 4– Observational – Descriptive Studies | Cancer |
| (Moseley et al., 2021) | 2021 | USA | retrospective study | Level 4– Observational – Descriptive Studies | solid |
| (Moss et al., 2021) | 2021 | UK | randomized controlled study | Level 1 – Experimental Designs | solid |
| (Nakamura et al., 2017) | 2017 | Japan | "our study" | Level 4– Observational – Descriptive Studies | solid |
| (Nezami et al., 2019) | 2019 | USA | retrospective study | Level 4– Observational – Descriptive Studies | solid, haem |
| (Nucci, Braga, Nouer, & Anaissie, 2018) | 2018 | Brazil | cohort study | Level 3 – Observational – Analytic Designs Level | solid, haem |
| (Oh et al., 2021) | 2021 | Korea | cohort study | Level 3 – Observational – Analytic Designs Level | solid |
| (Ohtake et al., 2018) | 2018 | Japan | observational study | Level 4– Observational – Descriptive Studies | haem |
| (Okazaki et al., 2019) | 2019 | Japan | cohort study | Level 3 – Observational – Analytic Designs Level | solid |
| (Pénichoux et al., 2022) | 2022 | France | retrospective study | Level 4– Observational – Descriptive Studies | haem |
| (Paquet, Boucher, Valenti, & Lindsay, 2017) | 2017 | Canada, Belfast - UK | randomized controlled trial | Level 1 – Experimental Designs | cancer |
| (E. J. Park et al., 2020; S. Park, Moon, Pai, & Kim, 2020) | 2020 | South Korea | retrospective study | Level 4– Observational – Descriptive Studies | cancer |
| (E. J. Park et al., 2021) | 2021 | Korea | randomized phase II trial | Level 1 – Experimental Designs | solid |
| (Patel et al., 2017) | 2017 | USA | observational study | Level 4– Observational – Descriptive Studies | cancer |
| (Peng, Wei, Li, Yuan, & Lin, 2022) | 2022 | China | cohort study | Level 3 – Observational – Analytic Designs Level | solid |
| (Perek et al., 2022) | 2022 | Israel | retrospective study | Level 4– Observational – Descriptive Studies | haem |
| (Picardi et al., 2019) | 2019 | Italy | randomized trial | Level 1 – Experimental Designs | haem |
| (Pike, Tan, & Burbridge, 2021) | 2021 | Canada | observational study | Level 4– Observational – Descriptive Studies | solid |
| (Pinelli, Balsorano, Mura, & Pittiruti, 2021) | 2021 | Italy | expert consensus | Level 5 – Expert Opinion and Bench Research | solid, haem |
| (Pinelli, Balsorano, et al., 2021; Pinelli, Pittiruti, et al., 2021) | 2021 | Italy, UK, Belgium, The Netherlands, France | international expert consensus statement | Level 4– Observational – Descriptive Studies | cancer |
| (Piredda et al., 2021) | 2021 | Italy | observational study | Level 4– Observational – Descriptive Studies | solid |
| (Platanaki, Zareifopoulos, Lagadinou, Tsiotsios, & Velissaris, 2021) | 2021 | Greece | retrospective study | Level 4– Observational – Descriptive Studies | solid |
| (Ploton et al., 2021) | 2021 | France | retrospective study | Level 4– Observational – Descriptive Studies | solid, haem |
| (Pu et al., 2020) | 2020 | China | meta-analysis | Level 2 – Quasi-experimental Designs | solid, haem |
| (Qi, Cheng, Yuan, & Zhang, 2020) | 2020 | China | retrospective study | Level 4– Observational – Descriptive Studies | solid |
| (Raad et al., 2018) | 2018 | USA | improvement project | Level 4– Observational – Descriptive Studies | solid, haem |
| (Rabelo-Silva et al., 2022) | 2022 | Brazil | cohort study | Level 3 – Observational – Analytic Designs Level | cancer |
| (Ranch-Lundin, Schedin, & Bjorkhem-Bergman, 2021) | 2021 | Sweden | retrospective study | Level 4– Observational – Descriptive Studies | cancer |
| (Rasero et al., 2018) | 2018 | Italy | observational study | Level 4– Observational – Descriptive Studies | solid, haem |
| (C. M. Rickard et al., 2017) | 2017 | Australia | randomised controlled trial | Level 2 – Quasi-experimental Designs | cancer |
| (Claire M. Rickard et al., 2021) | 2021 | Australia | pilot randomized controlled trial | Level 2 – Quasi-experimental Designs | cancer |
| (Rixecker et al., 2021) | 2021 | Germany | retrospective study | Level 4– Observational – Descriptive Studies | haem |
| (Rockholt, Thorarinsdottir, Lazarevic, Rundgren, & Kander, 2022) | 2022 | Sweden | observational study | Level 4– Observational – Descriptive Studies | haem |
| (Rowe, Arnold, & Spencer, 2020) | 2020 | USA | quality review | Level 4– Observational – Descriptive Studies | cancer |
| (Ruiz-Giardin et al., 2019) | 2019 | Spain | populational study | Level 4– Observational – Descriptive Studies | solid, haem |
| (Russo et al., 2019) | 2019 | Brazil | "our study" | Level 4– Observational – Descriptive Studies | haem |
| (Sacks et al., 2022) | 2022 | USA | chart review | Level 4– Observational – Descriptive Studies | solid |
| (Samuelson et al., 2018) | 2018 | UK | cohort study | Level 3 – Observational – Analytic Designs Level | haem |
| (Santacatalina-Roig, Espinar-de Las Heras, Ballesteros-Lizondo, Ibanez-Puchades, & Pescador-Marco, 2020) | 2020 | Spain | descriptive study | Level 4– Observational – Descriptive Studies | haem |
| (Sapkota, Sannur, & Naik, 2020) | 2020 | India | observational study | Level 4– Observational – Descriptive Studies | solid, haem |
| (Schears, Ferko, Syed, Arpino, & Alsbrooks, 2021) | 2021 | USA | meta-analysis | Level 1 – Experimental Designs | solid, haem |
| (Scrivens, Sabri, Bredeson, & McDiarmid, 2020) | 2020 | Canada | retrospective study | Level 4– Observational – Descriptive Studies | haem |
| (Seckold, Walker, Dwyer, & Signal, 2019) | 2019 | Australia | chart audit | Level 4– Observational – Descriptive Studies | solid |
| (Sengul, Ocakci, Guven, & Kaya, 2019) | 2019 | Turkey | randomized-experimental study | Level 1 – Experimental Designs | cancer |
| (Seo et al., 2017) | 2017 | Korea | retrospective study | Level 4– Observational – Descriptive Studies | cancer |
| (Sharp et al., 2021) | 2021 | Australia | cohort study | Level 3 – Observational – Analytic Designs Level | cancer |
| (Shibata et al., 2021) | 2021 | Japan | cohort study | Level 3 – Observational – Analytic Designs Level | solid, haem |
| (Shih, Teng, Chen, Chang, & Chen, 2022) | 2022 | Taiwan | observational study | Level 4– Observational – Descriptive Studies | haem |
| (Silva, Reichembach, Pontes, Souza, & Kusma, 2021) | 2021 | Brazil | randomized clinical trial | Level 2 – Quasi-experimental Designs | haem |
| (Simonetti et al., 2020) | 2020 | Italy | observational study | Level 4– Observational – Descriptive Studies | solid |
| (Simonetti et al., 2022) | 2022 | Italy | systematic review | Level 3 – Observational – Analytic Designs Level | solid, haem |
| (Skelton Iv et al., 2019) | 2019 | USA | retrospective study | Level 4– Observational – Descriptive Studies | cancer |
| (Skummer et al., 2020) | 2020 | USA | retrospective study | Level 4– Observational – Descriptive Studies | solid |
| (Slaughter, Keogh, Kynoch, & Brodribb, 2020) | 2020 | Australia | systematic review and meta-analysis | Level 1 – Experimental Designs | cancer |
| (Snarski et al., 2021) | 2021 | Poland, Spain, Sweden, Italy | observational study | Level 4– Observational – Descriptive Studies | haem |
| (Solinas et al., 2017) | 2017 | Italy | observational study. | Level 4– Observational – Descriptive Studies | solid |
| (X. Song et al., 2020) | 2020 | China | retrospective study | Level 4– Observational – Descriptive Studies | solid, haem |
| (Y. Song et al., 2020) | 2020 | China | "present study" | Level 4– Observational – Descriptive Studies | cancer |
| (Spires et al., 2018) | 2018 | USA | cohort study | Level 3 – Observational – Analytic Designs Level | cancer |
| (Suleman & McDiarmid, 2017) | 2017 | Canada | cohort study | Level 3 – Observational – Analytic Designs Level | solid, haem |
| (Suleman, Jarvis, Hadziomerovic, Carrier, & McDiarmid, 2019) | 2019 | Canada | cohort study | Level 3 – Observational – Analytic Designs Level | solid |
| (Suttle, Buffington, Madden, & Dawson, 2019) | 2019 | UK | quality improvement project, | Level 4– Observational – Descriptive Studies | haem |
| (Sze Yong et al., 2022) | 2022 | Malaysia | prospective study | Level 4– Observational – Descriptive Studies | cancer |
| (Tabatabaie, Kasumova, Eskander, et al., 2017) | 2017 | USA | narrative review | Level 4– Observational – Descriptive Studies | cancer |
| (Tabatabaie, Kasumova, Kent, et al., 2017) | 2017 | USA | "this study" | Level 4– Observational – Descriptive Studies | solid, haem |
| (Takashima, Ray-Barruel, Ullman, Keogh, & Rickard, 2017) | 2017 | Australia | scoping review | Level 4– Observational – Descriptive Studies | cancer |
| (Tan et al., 2019) | 2019 | China | retrospective study | Level 4– Observational – Descriptive Studies | solid |
| (T. Tang, Li, Wang, Li, & Geng, 2019) | 2019 | China | retrospective study | Level 4– Observational – Descriptive Studies | solid |
| (L. Tang et al., 2020) | 2020 | USA | retrospective study | Level 4– Observational – Descriptive Studies | solid, haem |
| (Taxbro et al., 2019) | 2019 | Sweden | randomised trial | Level 2 – Quasi-experimental Designs | solid |
| (Tian, Yin, Zhu, Zhang, & Zhang, 2021) | 2021 | China | retrospective study | Level 4– Observational – Descriptive Studies | cancer |
| (Tippit et al., 2018) | 2018 | USA | retrospective study | Level 4– Observational – Descriptive Studies | solid |
| (Trezza et al., 2021) | 2021 | Italy | cohort study | Level 3 – Observational – Analytic Designs Level | solid, haem |
| (Tsuruta et al., 2020) | 2020 | Japan | retrospective study | Level 4– Observational – Descriptive Studies | solid |
| (Tumay & Guner, 2021) | 2021 | Turkey | retrospective study | Level 4– Observational – Descriptive Studies | solid |
| (Amanda J. Ullman et al., 2019) | 2019 | Australia | secondary data analysis of randomised controlled trials and observational studies | Level 3 – Observational – Analytic Designs Level | cancer |
| (A. J. Ullman et al., 2022) | 2022 | USA, Australia | cohort study | Level 3 – Observational – Analytic Designs Level | haem |
| (Velioglu, Yuksel, & Sinmaz, 2019) | 2019 | Turkey | retrospective study | Level 3 – Observational – Analytic Designs Level | solid |
| (Verboom et al., 2017) | 2017 | The Netherlands | retrospective study | Level 4– Observational – Descriptive Studies | solid |
| (Vermeulin et al., 2018) | 2018 | France | "this study" | Level 4– Observational – Descriptive Studies | solid, haem |
| (Voog et al., 2018) | 2018 | Germany | observational study | Level 4– Observational – Descriptive Studies | solid, haem |
| (Voor in ’t holt et al., 2017) | 2017 | The Netherlands | systematic review and meta-analysis | Level 1 – Experimental Designs | solid, haem |
| (Wan et al., 2022) | 2022 | China | cohort study | Level 1 – Experimental Designs | cancer |
| (X.-J. Wang, 2017) | 2017 | PR China | "this study" | Level 4– Observational – Descriptive Studies | cancer |
| (Y. C. Wang, Lin, Chou, Lin, & Huang, 2017) | 2017 | Taiwan | cohort study | Level 3 – Observational – Analytic Designs Level | solid, haem |
| (G. Wang et al., 2020) | 2020 | China | observational study | Level 4– Observational – Descriptive Studies | solid |
| (G.-D. Wang et al., 2020) | 2020 | China | retro-prospective study design, | Level 4– Observational – Descriptive Studies | solid, haem |
| (G. Wang et al., 2021) | 2021 | China | retrospective study | Level 4– Observational – Descriptive Studies | solid, haem |
| (Webber & Maningo-Salinas, 2020) | 2020 | Australia & USA | descriptive study | Level 3 – Observational – Analytic Designs Level | solid, haem |
| (Webster et al., 2017) | 2017 | Australia | randomized controlled trial | Level 1 – Experimental Designs | cancer |
| (Winkler et al., 2021) | 2021 | USA | observational study | Level 4– Observational – Descriptive Studies | cancer |
| (S. Wu, Li, Zhang, Li, & Wang, 2018) | 2018 | China | meta-analysis | Level 2 – Quasi-experimental Designs | solid, haem |
| (X. Wu, Zhang, Chen, & Chen, 2021) | 2021 | China | systematic review and meta-analysis | Level 1 – Experimental Designs | cancer |
| (Xiong, Zhou, & Li, 2021) | 2021 | China | randomized controlled trial | Level 2 – Quasi-experimental Designs | solid |
| (Xie, Xu, Xu, & Huang, 2017) | 2017 | China | retrospective study | Level 4– Observational – Descriptive Studies | solid |
| (Xiong, Luo, & Chen, 2019) | 2019 | China | cross-sectional study | Level 4– Observational – Descriptive Studies | cancer |
| (Xiong et al., 2021) | 2021 | China | systematic review and meta-analysis | Level 2 – Quasi-experimental Designs | solid, haem |
| (Yan, Zhang, Luo, & Li, 2021) | 2021 | China | observational study | Level 4– Observational – Descriptive Studies | cancer |
| (S.-S. Yang & Ahn, 2018) | 2018 | Korea | retrospective study | Level 4– Observational – Descriptive Studies | solid |
| (W. J. Yang, Song, Seo, & Park, 2021) | 2021 | Korea | retrospective study | Level 4– Observational – Descriptive Studies | cancer |
| (Yanik, Karamustafaoglu, Karatas, & Yoruk, 2018) | 2018 | Turkey | "our study" | Level 4– Observational – Descriptive Studies | solid |
| (Yildiz et al., 2019) | 2019 | Turkey | retrospective study | Level 4– Observational – Descriptive Studies | haem |
| (L. Yin & Li, 2020) | 2020 | China | retrospective study | Level 4– Observational – Descriptive Studies | solid |
| (Y. X. Yin et al., 2020) | 2020 | China | randomized study | Level 2 – Quasi-experimental Designs | solid |
| (Ying, Liping, Yanhong, Zhulin, & Liang, 2020) | 2020 | China | cross-sectional study | Level 3 – Observational – Analytic Designs Level | solid |
| (L. Yu et al., 2017) | 2017 | China | retrospective study | Level 4– Observational – Descriptive Studies | solid |
| (X. Y. Yu, Xu, Li, & Jiang, 2018) | 2018 | China | retrospective study | Level 4– Observational – Descriptive Studies | solid |
| (Yun & Yang, 2021) | 2021 | South Korea | cohort study | Level 3 – Observational – Analytic Designs Level | cancer |
| (Zabicki, Limphaibool, Veilemand Holstad, & Perkowska, 2019) | 2019 | Poland | retrospective study | Level 4– Observational – Descriptive Studies | solid |
| (Zanwar et al., 2019) | 2019 | India | retrospective study | Level 4– Observational – Descriptive Studies | haem |
| (Zerla et al., 2017) | 2017 | Italy | observational study | Level 4– Observational – Descriptive Studies | cancer |
| (M. Zhang, Kang, & Li, 2017) | 2017 | China | comparative study | Level 3 – Observational – Analytic Designs Level | solid |
| (S. Zhang et al., 2018) | 2018 | USA | retrospective study | Level 4– Observational – Descriptive Studies | haem |
| (Y. Zhang, Zhang, Chen, & Zhao, 2021) | 2021 | China | self-controlled study | Level 4– Observational – Descriptive Studies | solid |
| (Y. Zhang et al., 2021) | 2021 | China | observational study | Level 4– Observational – Descriptive Studies | solid |
| (H. Zhao et al., 2018) | 2018 | China | observational study | Level 4– Observational – Descriptive Studies | solid, haem |
| (Y. Zhao, Bian, & Yang, 2022) | 2022 | China | observational study | Level 4– Observational – Descriptive Studies | cancer |
| (Zhong, Wang, & Huang, 2021) | 2021 | China | "this study" | Level 4– Observational – Descriptive Studies | cancer |
| (Zhou, Yang, Wang, & Qin, 2017) | 2017 | China | "this study" | Level 4– Observational – Descriptive Studies | solid |
| (Ziegler et al., 2019) | 2017 | USA | cohort study | Level 3 – Observational – Analytic Designs Level | haem |

**References:**

Aghamohammadi, D., Fakhari, S., Ataei, Y., Bilehjani, E., & Jafari, M. (2017). Totally implantable venous access port infection in northwest of Iran. *Crescent Journal of Medical and Biological Sciences, 4*(3), 126-130.

Agrawal, S. K., Gautam, H., Choudhary, A. H., Das, B. K., Kumar, L., & Kapil, A. (2019). Central line-associated bloodstream infections in cancer patients: An experience from a tertiary care cancer centre. *Indian journal of medical microbiology, 37*(3), 376-380. doi:<https://dx.doi.org/10.4103/ijmm.IJMM_19_352>

Ahmad, A., Hjerming, M., Kjeldsen, L., Bjerrum, O. W., Moser, C., Classen, V., & Dahl, A. (2019). Hydrochloric acid prolongs the lifetime of central venous catheters in haematologic patients with bacteraemia. *Danish medical journal, 66*(5), A5544.

Akhtar, N., & Lee, L. (2021). Utilization and Complications of Central Venous Access Devices in Oncology Patients. *Current oncology (Toronto, Ont.), 28*(1), 367-377. doi:<https://dx.doi.org/10.3390/curroncol28010039>

Alfonso Alvarez-Rodriguez, J., Garcia-Suarez, M., Fernandez-Garcia, D., Mendez-Martinez, C., & Gomez-Salgado, J. (2018). Analysis of peripheral central venous access ports at the forearm: An observational study. *European journal of cancer care, 27*(6), e12929. doi:<https://dx.doi.org/10.1111/ecc.12929>

Alkindi, S. Y., Chai-Adisaksopha, C., Cheah, M., & Linkins, L.-A. (2018). Management of cancer-associated upper extremity deep vein thrombosis with and without venous catheters at a tertiary care center. *Thrombosis research, 166*, 92-95. doi:<https://dx.doi.org/10.1016/j.thromres.2018.03.020>

Ammar, G., Almashaikh, E., Ibdah, A., Shajrawi, W., Awawdeh, S., Al Mousa, A., . . . Alkharabsheh, M. (2019). Impact of Early Dressing Removal on Tunneled Central Venous Catheters: A Piloting Study. *Asian Pac J Cancer Prev, 20*(9), 2693-2697. doi:10.31557/apjcp.2019.20.9.2693

Anbar, R., Avci, D., & Cetinkaya, A. (2017). Port catheter complications and thrombosis issues: Assessment of 114 patients with port catheter implantation by single surgeon. *Biomedical Research and Therapy, 4*(12), 1898-1910. doi:<http://dx.doi.org/10.15419/bmrat.v4i12.401>

Annetta, M. G., Ostroff, M., Marche, B., Emoli, A., Musarò, A., Celentano, D., . . . Pittiruti, M. (2021). Chest-to-arm tunneling: A novel technique for medium/long term venous access devices. *J Vasc Access*, 11297298211026825. doi:10.1177/11297298211026825

Aribas, B. K., Tiken, R., Aribas, O., Uylar, T., Akdulum, I., Turker, I., . . . Caglar, E. (2017). Factors on Patency Periods of Subcutaneous Central Venous Port: Long-Term Results of 1,408 Patients. *Iranian Journal of Radiology, 14*(2), 1-Jul. doi:10.5812/iranjradiol.36816

Bademler, S., Ucuncu, M., Yildirim, I., & Karanlik, H. (2019). Risk factors for complications in cancer patients with totally implantable access ports: A retrospective study and review of the literature. *The Journal of international medical research, 47*(2), 702-709. doi:<https://dx.doi.org/10.1177/0300060518808167>

Bai, X., Gu, X., Cheng, L., Yuan, Q., Jing, J., Jin, Y., . . . Jiang, Y. (2017). Clinical diagnosis and treatment of peripherally inserted central catheter related upper extremity deep venous thrombosis. *Biomedical research (india), 28*(22), 9707-9711.

Balsorano, P., Romagnoli, S., Pinelli, F., Virgili, G., Villa, G., De Gaudio, A. R., & Pittiruti, M. (2019). Peripherally inserted central catheter-related thrombosis rate in modern vascular access era-when insertion technique matters: A systematic review and meta-analysis. *Journal of vascular access*. doi:<http://dx.doi.org/10.1177/1129729819852203>

Ban, T., Fujiwara, S. I., Murahashi, R., Nakajima, H., Ikeda, T., Matsuoka, S., . . . Kanda, Y. (2022). Risk Factors for Complications Associated with Peripherally Inserted Central Catheters During Induction Chemotherapy for Acute Myeloid Leukemia. *Intern Med, 61*(7), 989-995. doi:10.2169/internalmedicine.8184-21

Baumann Kreuziger, L., Gaddh, M., Onadeko, O., George, G., Wang, T. F., Oo, T. H., . . . Billett, H. H. (2021). Treatment of catheter-related thrombosis in patients with hematologic malignancies: A Venous thromboEmbolism Network U.S. retrospective cohort study. *Thromb Res, 202*, 155-161. doi:10.1016/j.thromres.2021.03.021

Belloni, S., Caruso, R., Cattani, D., Mandelli, G., Donizetti, D., Mazzoleni, B., & Tedeschi, M. (2022). Occurrence rate and risk factors for long-term central line-associated bloodstream infections in patients with cancer: A systematic review. *Worldviews Evid Based Nurs, 19*(2), 100-111. doi:10.1111/wvn.12574

Bertoglio, S., Annetta, M. G., Brescia, F., Emoli, A., Fabiani, F., Fino, M., . . . Pittiruti, M. (2022). A multicenter retrospective study on 4480 implanted PICC-ports: A GAVeCeLT project. *J Vasc Access*, 11297298211067683. doi:10.1177/11297298211067683

Bertoglio, S., Cafiero, F., Meszaros, P., Varaldo, E., Blondeaux, E., Molinelli, C., & Minuto, M. (2020). PICC-PORT totally implantable vascular access device in breast cancer patients undergoing chemotherapy. *The journal of vascular access, 21*(4), 460-466. doi:<https://dx.doi.org/10.1177/1129729819884482>

Bessis, S., Cassir, N., Meddeb, L., Remacle, A. B., Soussan, J., Vidal, V., . . . Brouqui, P. (2020). Early mortality attributable to PICC-lines in 4 public hospitals of Marseille from 2010 to 2016 (Revised V3). *Medicine (Baltimore), 99*(1), e18494. doi:10.1097/md.0000000000018494

Beypinar, I., Demir, H., Uysal, M., Araz, M., & Beypinar, D. (2020). The comparison of central venous port catheters in gastrointestinal cancer treatment. *Journal of Oncological Science, 6*(1), Oct-14. doi:<http://dx.doi.org/10.37047/jos.2019-73122>

Boll, B., Schalk, E., Buchheidt, D., Hasenkamp, J., Kiehl, M., Kiderlen, T. R., . . . Hentrich, M. (2021). Central venous catheter-related infections in hematology and oncology: 2020 updated guidelines on diagnosis, management, and prevention by the Infectious Diseases Working Party (AGIHO) of the German Society of Hematology and Medical Oncology (DGHO). *Annals of hematology, 100*(1), 239-259. doi:<https://dx.doi.org/10.1007/s00277-020-04286-x>

Bouzidi, H., Emirian, A., Marty, A., Chachaty, E., Laplanche, A., Gachot, B., & Blot, F. (2018). Differential time to positivity of central and peripheral blood cultures is inaccurate for the diagnosis of Staphylococcus aureus long-term catheter-related sepsis. *The Journal of hospital infection, 99*(2), 192-199. doi:<https://dx.doi.org/10.1016/j.jhin.2018.01.010>

Brescia, F., Pittiruti, M., Roveredo, L., Zanier, C., Morabito, A., Santarossa, E., . . . Fabiani, F. (2021). Subcutaneously anchored securement for peripherally inserted central catheters: Immediate, early, and late complications. *J Vasc Access*, 11297298211025430. doi:10.1177/11297298211025430

Brito, A. R. d. O., Nishinari, K., Saad, P. F., Saad, K. R., Pereira, M. A. T., Emidio, S. C. D., . . . Soares, B. L. F. (2018). Comparison between Saline Solution Containing Heparin versus Saline Solution in the Lock of Totally Implantable Catheters. *Annals of vascular surgery, 47*, 85-89. doi:<https://dx.doi.org/10.1016/j.avsg.2017.09.015>

Broadhurst, D., Moureau, N., & Ullman, A. J. (2017). Management of Central Venous Access Device-Associated Skin Impairment: An Evidence-Based Algorithm. *J Wound Ostomy Continence Nurs, 44*(3), 211-220. doi:10.1097/won.0000000000000322

Burbridge, B., Plewes, C., Stoneham, G., Szkup, P., Otani, R., Babyn, P., & Bryce, R. (2018). Randomized Clinical Trial Evaluating Complications and Complication-Related Removal of Arm-Situated Power-Injectable and Non-Power-Injectable Totally Implanted Venous Access Devices among Cancer Patients. *Journal of vascular and interventional radiology : JVIR, 29*(5), 648-656.e643. doi:<https://dx.doi.org/10.1016/j.jvir.2017.11.028>

Busch, J. D., Herrmann, J., Adam, G., Ittrich, H., Vens, M., & Mahler, C. (2017). Complication Rates Observed in Silicone and Polyurethane Catheters of Totally Implanted Central Venous Access Devices Implanted in the Upper Arm. *Journal of vascular and interventional radiology, 28*(8), 1177-1183. doi:<http://dx.doi.org/10.1016/j.jvir.2017.04.024>

Busch, J. D., Vens, M., Herrmann, J., Adam, G., & Ittrich, H. (2017). Material Failure of Silicone Catheter Lines: A Retrospective Review of Partial and Complete Ruptures in 553 Patients. *AJR Am J Roentgenol, 208*(2), 464-469. doi:10.2214/ajr.16.16540

Calò, F., Retamar, P., Martínez Pérez-Crespo, P. M., Lanz-García, J., Sousa, A., Goikoetxea, J., . . . López-Cortés, L. E. (2020). Catheter-related bloodstream infections: predictive factors for Gram-negative bacteria aetiology and 30 day mortality in a multicentre prospective cohort. *J Antimicrob Chemother, 75*(10), 3056-3061. doi:10.1093/jac/dkaa262

Campagna, S., Berchialla, P., Gonella, S., Dimonte, V., Mussa, B., Morano, G., . . . Corona, G. (2019). Can Peripherally Inserted Central Catheters Be Safely Placed in Patients with Cancer Receiving Chemotherapy? A Retrospective Study of Almost 400,000 Catheter-Days. *Oncologist, 24*(9), e953-e959. doi:<http://dx.doi.org/10.1634/theoncologist.2018-0281>

Campagna, S., Gonella, S., Berchialla, P., Rigo, C., Morano, G., Zerla, P. A., . . . Mussa, B. (2019). A retrospective study of the safety of over 100,000 peripherally-inserted central catheters days for parenteral supportive treatments. *Research in nursing & health, 42*(3), 198-204. doi:<https://dx.doi.org/10.1002/nur.21939>

Capozzi, V. A., Monfardini, L., Sozzi, G., Armano, G., Butera, D., Scarpelli, E., . . . Berretta, R. (2021). Peripherally Inserted Central Venous Catheters (PICC) versus totally implantable venous access device (PORT) for chemotherapy administration: a meta-analysis on gynecological cancer patients. *Acta Biomed, 92*(5), e2021257. doi:10.23750/abm.v92i5.11844

Caris, M. G., de Jonge, N. A., Punt, H. J., Salet, D. M., de Jong, V. M. T., Lissenberg-Witte, B. I., . . . Janssen, J. (2022). Indwelling time of peripherally inserted central catheters and incidence of bloodstream infections in haematology patients: a cohort study. *Antimicrob Resist Infect Control, 11*(1), 37. doi:10.1186/s13756-022-01069-z

Carvalho Castanho, L. E., Nogueira dos Santos, B., Salles Margatho, A., Merizio Martins Braga, F. T., Diniz dos Reis, P. E., de Oliveira, M. C., & de Campos Pereira Silveira, R. C. (2020). Chlorhexidine gel dressing in hematopoietic stem cell transplantation. *Acta Paulista de Enfermagem, 33*(3), 1-Oct. doi:10.37689/actaape/2020AO0307

Chaftari, A. M., Hachem, R., Raad, S., Jiang, Y., Natividad, E., Chaftari, P., & Raad, I. (2018). Unnecessary Removal of Central Venous Catheters in Cancer Patients with Bloodstream Infections. *Infection control and hospital epidemiology, 39*(2), 222-225. doi:<https://dx.doi.org/10.1017/ice.2017.284>

Chaftari, P., Chaftari, A. M., Adachi, J., Hachem, R., Raad, S., Natividad, E., . . . Raad, I. (2017). Improvement in the diagnosis of catheter-related bloodstream infections in a tertiary cancer center. *Am J Infect Control, 45*(3), e34-e39. doi:10.1016/j.ajic.2016.12.009

Chan, R. J., Northfield, S., Larsen, E., Mihala, G., Ullman, A., Hancock, P., . . . et al. (2017). Central venous Access device SeCurement And Dressing Effectiveness for peripherally inserted central catheters in adult acute hospital patients (CASCADE): a pilot randomised controlled trial. *Trials, 18*(1), 458. doi:10.1186/s13063-017-2207-x

Chang, D. H., Mammadov, K., Hickethier, T., Borggrefe, J., Hellmich, M., Maintz, D., & Kabbasch, C. (2017). Fibrin sheaths in central venous port catheters: treatment with low-dose, single injection of urokinase on an outpatient basis. *Ther Clin Risk Manag, 13*, 111-115. doi:10.2147/tcrm.S125130

Chen, K., Beeraka, N. M., Gu, Y., Li, J., Sinelnikov, M., Han, N., & Lu, P. (2021). Totally Implantable Venous Access Port Systems: Implant Depth-based Complications in Breast Cancer Therapy - A Comparative Study. *Curr Pharm Des, 27*(46), 4671-4676. doi:10.2174/1381612827666210901170522

Chen, M. H., Hwang, W. L., Chang, K. H., Chiang, L. C. J., & Teng, C. L. J. (2017). Application of peripherally inserted central catheter in acute myeloid leukaemia patients undergoing induction chemotherapy. *European journal of cancer care, 26*(6). doi:<https://dx.doi.org/10.1111/ecc.12627>

Chen, P., Zhu, B., Wan, G., & Qin, L. (2021). The incidence of asymptomatic thrombosis related to peripherally inserted central catheter in adults: A systematic review and meta-analysis People's. *Nurs Open, 8*(5), 2249-2261. doi:10.1002/nop2.811

Chen, X.-S., Wu, X.-H., Chen, L.-C., Zhang, T.-T., & Liu, G.-L. (2021). Heparin versus 0.9% saline solution to maintain patency of totally implanted venous access ports in cancer patients: A systematic review and meta-analysis. *International journal of nursing practice*, e12913. doi:<http://dx.doi.org/10.1111/ijn.12913>

Chen, Y., Chen, H., Yang, J., Jin, W., Fu, D., Liu, M., . . . Chen, Y. (2020). Patterns and risk factors of peripherally inserted central venous catheter-related symptomatic thrombosis events in patients with malignant tumors receiving chemotherapy. *Journal of vascular surgery. Venous and lymphatic disorders, 8*(6), 919-929. doi:<https://dx.doi.org/10.1016/j.jvsv.2020.01.010>

Choksi, A., Finnegan, K., & Etezadi, V. (2020). Does systemic antibiotic prophylaxis prior to the placement of totally implantable venous access devices reduce early infection? A retrospective study of 1,485 cases at a large academic institution. *American journal of infection control, 48*(1), 95-99. doi:<https://dx.doi.org/10.1016/j.ajic.2019.06.028>

Chong, H. Y., Lai, N. M., Apisarnthanarak, A., & Chaiyakunapruk, N. (2017). Comparative Efficacy of Antimicrobial Central Venous Catheters in Reducing Catheter-Related Bloodstream Infections in Adults: Abridged Cochrane Systematic Review and Network Meta-Analysis. *Clinical infectious diseases : an official publication of the Infectious Diseases Society of America, 64*(suppl_2), S131-S140. doi:<https://dx.doi.org/10.1093/cid/cix019>

Chopra, V., Kaatz, S., Grant, P., Swaminathan, L., Boldenow, T., Conlon, A., . . . Flanders, S. A. (2018). Risk of Venous Thromboembolism Following Peripherally Inserted Central Catheter Exchange: An Analysis of 23,000 Hospitalized Patients. *The American journal of medicine, 131*(6), 651-660. doi:<https://dx.doi.org/10.1016/j.amjmed.2018.01.017>

Chopra, V., O'Malley, M., Horowitz, J., Zhang, Q., McLaughlin, E., Saint, S., . . . Flanders, S. (2022). Improving peripherally inserted central catheter appropriateness and reducing device-related complications: a quasiexperimental study in 52 Michigan hospitals. *BMJ Qual Saf, 31*(1), 23-30. doi:10.1136/bmjqs-2021-013015

Chou, P. L., Fu, J. Y., Cheng, C. H., Chu, Y., Wu, C. F., Ko, P. J., . . . Wu, C. Y. (2019). Current port maintenance strategies are insufficient: View based on actual presentations of implanted ports. *Medicine (Baltimore), 98*(44), e17757. doi:10.1097/md.0000000000017757

Clari, M., Spoto, M., Franceschi, G., Acuto, M., Tonella, S., Caristia, S., . . . Dal Molin, A. (2021). Short Versus Long Timing of Flushing of Totally Implantable Venous Access Devices When Not Used Routinely: A Systematic Review and Meta-analysis. *Cancer nursing, 44*(3), 205-213. doi:10.1097/NCC.0000000000000819

Clatot, F., Fontanilles, M., Lefebvre, L., Lequesne, J., Veyret, C., Alexandru, C., . . . et al. (2020). Randomised phase II trial evaluating the safety of peripherally inserted catheters versus implanted port catheters during adjuvant chemotherapy in patients with early breast cancer. *European journal of cancer (Oxford, England : 1990), 126*, 116‐124. doi:10.1016/j.ejca.2019.11.022

Cornillon, J., Martignoles, J. A., Tavernier-Tardy, E., Gire, M., Martinez, P., Tranchan, C., . . . Hacquard, B. (2017). Prospective evaluation of systematic use of peripherally inserted central catheters (PICC lines) for the home care after allogeneic hematopoietic stem cells transplantation. *Supportive Care in Cancer, 25*(9), 2843-2847. doi:<http://dx.doi.org/10.1007/s00520-017-3699-3>

Corti, F., Brambilla, M., Manglaviti, S., Di Vico, L., Pisanu, M. N., Facchinetti, C., . . . de Braud, F. (2021). Comparison of outcomes of central venous catheters in patients with solid and hematologic neoplasms: an Italian real-world analysis. *Tumori, 107*(1), 17-25. doi:<http://dx.doi.org/10.1177/0300891620931172>

Cotogni, P., Mussa, B., Degiorgis, C., De Francesco, A., & Pittiruti, M. (2021). Comparative Complication Rates of 854 Central Venous Access Devices for Home Parenteral Nutrition in Cancer Patients: A Prospective Study of Over 169,000 Catheter-Days. *JPEN Journal of Parenteral & Enteral Nutrition, 45*(4), 768-776. doi:10.1002/jpen.1939

Cruz-Aguilar, R., Carney, J., Mondaini, V., Vehreschild, M. J. G. T., Griskaitis, M., Salmanton-García, J., . . . Farowski, F. (2021). A quality improvement study on the reduction of central venous catheter-associated bloodstream infections by use of self-disinfecting venous access caps (STERILE). *American journal of infection control, 49*(5), 586-592. doi:10.1016/j.ajic.2020.09.002

D'Souza, P. C., Kumar, S., Kakaria, A., Al-Sukaiti, R., Al-Baimani, K., Hamid, R. S., . . . Al-Moundhri, M. S. (2021). Complications and Management of Totally Implantable Central Venous Access Ports in Cancer Patients at a University Hospital in Oman. *Sultan Qaboos Univ Med J, 21*(1), e103-e109. doi:10.18295/squmj.2021.21.01.014

Da Costa, A. C. C., Vieira, N. N. P., Vasques, C. I., Ferreira, E. B., Dos Reis, P. E. D., & Guerra, E. N. S. (2019). Interventions for occluded central venous catheters: A meta-analysis. *Pediatrics, 144*(6), e20183789. doi:<http://dx.doi.org/10.1542/peds.2018-3789>

Dai, C., Li, J., Guo, X., Li, Q.-M., Fan, Y.-Y., & Qin, H.-Y. (2020). Effect of tunneled and nontunneled peripherally inserted central catheter placement: A randomized controlled trial. *Journal of vascular access, 21*(4), 511-519. doi:<http://dx.doi.org/10.1177/1129729819888120>

Dang, F. P., Li, H. J., & Tian, J. H. (2019). Comparative efficacy of 13 antimicrobial dressings and different securement devices in reducing catheter-related bloodstream infections: A Bayesian network meta-analysis. *Medicine (Baltimore), 98*(14), e14940. doi:10.1097/md.0000000000014940

Davies, G. A., Lazo-Langner, A., Gandara, E., Rodger, M., Tagalakis, V., Louzada, M., . . . Kovacs, M. J. (2018). A prospective study of Rivaroxaban for central venous catheter associated upper extremity deep vein thrombosis in cancer patients (Catheter 2). *Thrombosis research, 162*, 88-92. doi:<https://dx.doi.org/10.1016/j.thromres.2017.04.003>

de Campos Pereira Silveira, R. C., Dos Reis, P. E. D., Ferreira, E. B., Braga, F., Galvão, C. M., & Clark, A. M. (2020). Dressings for the central venous catheter to prevent infection in patients undergoing hematopoietic stem cell transplantation: a systematic review and meta-analysis. *Support Care Cancer, 28*(2), 425-438. doi:10.1007/s00520-019-05065-9

de la Cruz-Hernandez, I., Cornejo-Juarez, P., Tellez-Miranda, O., Barrera-Perez, L., Sandoval-Hernandez, S., Vilar-Compte, D., . . . Volkow, P. (2020). Microbiology and prevalence of E2SKAPE-resistant strains in catheter-related bloodstream infections in patients with cancer. *American journal of infection control, 48*(1), 40-45. doi:<https://dx.doi.org/10.1016/j.ajic.2019.06.008>

de Mooij, C. E. M., van der Velden, W. J. F. M., van Groningen, L. F. J., Blijlevens, N. M. A., Verweij, P. E., Meijer, C., . . . de Haan, A. F. J. (2020). Surveillance of catheter-related bloodstream infections in haemato-oncology patients: comparison of two definitions. *Journal of Hospital Infection, 105*(4), 686-690. doi:<http://dx.doi.org/10.1016/j.jhin.2020.04.027>

Decousus, H., Bourmaud, A., Fournel, P., Bertoletti, L., Labruyère, C., Presles, E., . . . Chauvin, F. (2018). Cancer-associated thrombosis in patients with implanted ports: a prospective multicenter French cohort study (ONCOCIP). *Blood, 132*(7), 707-716. doi:10.1182/blood-2018-03-837153

Diaz, J. A., Rai, S. N., Wu, X., Chao, J. H., Dias, A. L., & Kloecker, G. H. (2017). Phase II Trial on Extending the Maintenance Flushing Interval of Implanted Ports. *J Oncol Pract, 13*(1), e22-e28. doi:10.1200/jop.2016.010843

Dinçer, M., Kocakuşak, A., Hut, A., Gür, Ü., Çıtlak, G., & Akıncı, M. (2019). Comparison of Two Different Central Venous Access Device Insertion Techniques: No Evil in Details. *Medical Bulletin of Haseki / Haseki Tip Bulteni, 57*(1), Sep-14. doi:10.4274/haseki.galenos.2018.4459

Egnatios, D., & Gloria, C. (2021). Implanted Port Patency: Comparing Heparin and Normal Saline. *Clin J Oncol Nurs, 25*(2), 169-173. doi:10.1188/21.Cjon.169-173

El-Balat, A., Schmeil, I., Karn, T., Holtrich, U., Mavrova-Risteska, L., Rody, A., . . . Youssef, A. (2018). Catheter-related complications of subcutaneous implantable venous access devices in breast cancer patients. *In Vivo, 32*(5), 1275-1281. doi:<http://dx.doi.org/10.21873/invivo.11377>

El Boghdadly, Z., Zhao, Q., Koutou, J., Lustberg, M. E., Ludwig, M., Liscynesky, C., & Choe, H. (2022). Evaluation of central line salvage for mucosal barrier injury laboratory-confirmed bloodstream infection (MBI-LCBI) management practices in patients with hematologic malignancies. *Leuk Lymphoma, 63*(6), 1455-1463. doi:10.1080/10428194.2021.2020778

Eldeeb, H., Al-Asadi, O., & Almusarhed, M. (2019). Predictive risk factors of venous thromboembolism (VTE) associated with peripherally inserted central catheters (PICC) in ambulant solid cancer patients: Retrospective single Centre cohort study. *Thrombosis journal, 17*(1), 191. doi:<http://dx.doi.org/10.1186/s12959-019-0191-y>

Fang, S., Jiang, Y., Yang, J., Song, L., & Liu, Y. (2017). Comparison of three types of central venous catheters in patients with malignant tumor receiving chemotherapy. *Patient preference and adherence, 11*, 1197-1204. doi:<http://dx.doi.org/10.2147/PPA.S142556>

Fares, J., Khalil, M., Chaftari, A. M., Hachem, R., Jiang, Y., Kantarjian, H. M., & Raad, II. (2019). Impact of Catheter Management on Clinical Outcome in Adult Cancer Patients With Gram-Negative Bacteremia. *Open Forum Infect Dis, 6*(10), ofz357. doi:10.1093/ofid/ofz357

Feng, Y., Zheng, R., Fu, Y., Xiang, Q., Yue, Z., Li, J., . . . Jiang, Y. (2021). Assessing the thrombosis risk of peripherally inserted central catheters in cancer patients using Caprini risk assessment model: a prospective cohort study. *Support Care Cancer, 29*(9), 5047-5055. doi:10.1007/s00520-021-06073-4

Fornaro, C., Piubeni, M., Tovazzi, V., Cosentini, D., Gelmi, M., Rota, G., . . . Conti, E. (2019). Eight-week interval in flushing and locking port-a-cath in cancer patients: A single-institution experience and systematic review. *Eur J Cancer Care (Engl), 28*(2), e12978. doi:10.1111/ecc.12978

Fu, X., Lu, P., Wang, C., & Ye, G. (2019). Analysis of the risk factors of peripherally inserted central catheter-associated venous thrombosis after chemotherapy in patients with lung cancer. *International journal of clinical and experimental medicine, 12*(5), 5852-5859.

Furuhashi, S., Morita, Y., Ida, S., Muraki, R., Kitajima, R., Suzuki, K., . . . Takeuchi, H. (2021). Risk Factors for Totally Implantable Central Venous Access Port-related Infection in Patients With Malignancy. *Anticancer research, 41*(3), 1547-1553. doi:<https://dx.doi.org/10.21873/anticanres.14914>

Gilardi, E., Piano, A., Chellini, P., Fiori, B., Dolcetti, L., Pittiruti, M., & Scoppettuolo, G. (2021). Reduction of bacterial colonization at the exit site of peripherally inserted central catheters: A comparison between chlorhexidine-releasing sponge dressings and cyano-acrylate. *The journal of vascular access, 22*(4), 597-601. doi:<https://dx.doi.org/10.1177/1129729820954743>

Gonzalez, S., Jimenez, P., Saavedra, P., Macias, D., Loza, A., Leon, C., . . . Ruiz-Santana, S. (2021). Five-year outcome of peripherally inserted central catheters in adults: a separated infectious and thrombotic complications analysis. *Infection control and hospital epidemiology, 42*(7), 833-841. doi:<https://dx.doi.org/10.1017/ice.2020.1300>

Grau, D., Clarivet, B., Lotthe, A., Bommart, S., & Parer, S. (2017). Complications with peripherally inserted central catheters (PICCs) used in hospitalized patients and outpatients: a prospective cohort study. *Antimicrobial resistance and infection control, 6*, 18. doi:<https://dx.doi.org/10.1186/s13756-016-0161-0>

Gray, K. L., Benson, H. L., Pearce, C. L., Steidley, I. G., Bachman, A. M., & Adamski, J. (2019). Implementation and 2-year outcomes of the first FDA-approved implantable apheresis vascular access device. *Transfusion, 59*(11), 3461-3467. doi:<http://dx.doi.org/10.1111/trf.15512>

Guan, X., Yan, H., Zhang, J., Li, Y., & Zhou, Y. (2022). Risk factors of infection of totally implantable venous access port: A retrospective study. *J Vasc Access*, 11297298221085230. doi:10.1177/11297298221085230

Gudiol, C., Arnan, M., Aguilar-Guisado, M., Royo-Cebrecos, C., Sanchez-Orteg, I., Montero, I., . . . et al. (2020). A randomized, double-blind, placebo-controlled trial (TAURCAT Study) of citrate lock solution for prevention of endoluminal central venous catheter infection in neutropenic hematological patients. *Antimicrobial agents and chemotherapy, 64*(2).

GÜR, Ö., DonbaloĞLu, M. O., & GÜRkan, S. (2018). Comparison of Clinical Follow-up and Complications according to Cancer Types in Patients with Permanent Port Catheter Insertion due to Malignancy. *Duzce Medical Journal, 20*(3), 59-62. doi:10.18678/dtfd.469695

Haggstrom, L., Parmar, G., & Brungs, D. (2020). Central Venous Catheter Thrombosis in Cancer: A Multi-Centre Retrospective Study Investigating Risk Factors and Contemporary Trends in Management. *Clinical Medicine Insights. Oncology, 14*, 1.17955E+17915. doi:<https://dx.doi.org/10.1177/1179554920953097>

Hallam, C., Jackson, T., Rajgopal, A., & Russell, B. (2018). Establishing catheter-related bloodstream infection surveillance to drive improvement. *Journal of infection prevention, 19*(4), 160-166. doi:10.1177/1757177418767759

Harrold, K., Martin, A., & Bhuva, N. (2019). A prospective audit evaluating use of urokinase in oncology patients with occluded central venous access devices. *British journal of nursing (Mark Allen Publishing), 28*(19), S30-S36. doi:<https://dx.doi.org/10.12968/bjon.2019.28.19.S30>

Hashimoto, Y., Fukuta, T., Maruyama, J., Omura, H., & Tanaka, T. (2017). Experience of Peripherally Inserted Central Venous Catheter in Patients with Hematologic Diseases. *Internal medicine (Tokyo, Japan), 56*(4), 389-393. doi:<https://dx.doi.org/10.2169/internalmedicine.56.7625>

Hashimoto, Y., Hosoda, R., Omura, H., & Tanaka, T. (2021). Catheter-related bloodstream infection associated with multiple insertions of the peripherally inserted central catheter in patients with hematological disorders. *Sci Rep, 11*(1), 12209. doi:10.1038/s41598-021-91749-4

He, E., Ye, K., & Zheng, H. (2021). Clinical effect and safety of venous access ports and peripherally inserted central catheters in patients receiving tumor chemotherapy: a systematic review and meta-analysis. *Ann Palliat Med, 10*(8), 9105-9113. doi:10.21037/apm-21-1926

Heidenreich, D., Hansen, E., Kreil, S., Nolte, F., Jawhar, M., Hecht, A., . . . Klein, S. A. (2022). The insertion site is the main risk factor for central venous catheter-related complications in patients with hematologic malignancies. *Am J Hematol, 97*(3), 303-310. doi:10.1002/ajh.26445

Heidenreich, D., Hansen, E., Kreil, S., Nolte, F., Jawhar, M., Hecht de Gutierrez, A., . . . Klein, S. A. (2020). Influence of the Insertion Site on Central Venous Catheter-Related Complications in Patients Undergoing Allogeneic Hematopoietic Cell Transplantation. *Biol Blood Marrow Transplant, 26*(6), 1189-1194. doi:10.1016/j.bbmt.2020.02.007

Hill, S., Hamblett, I., Brady, S., Vasileukaya, S., Zuzuarregui, I., & Martin, F. (2019). Central venous access device-related sheaths: a predictor of infective and thrombotic incidence? *British journal of nursing (Mark Allen Publishing), 28*(19), S10-S18. doi:<https://dx.doi.org/10.12968/bjon.2019.28.19.S10>

Hong, S., Seo, T. S., Song, M. G., Seol, H. Y., Suh, S. I., & Ryoo, I. S. (2019). Clinical outcomes of totally implantable venous access port placement via the axillary vein in patients with head and neck malignancy. *J Vasc Access, 20*(2), 134-139. doi:10.1177/1129729818781270

Hoppe, A., Rupa-Matysek, J., Małecki, B., Dytfeld, D., Hoppe, K., & Gil, L. (2021). Risk Factors for Catheter-Related Thrombosis in Multiple Myeloma Patients Undergoing Autologous Stem Cell Transplantation. *Medicina (Kaunas), 57*(10). doi:10.3390/medicina57101020

Htun, K. T., Ma, M. J. Y., & Lee, A. Y. Y. (2018). Incidence and outcomes of catheter related thrombosis (CRT) in patients with acute leukemia using a platelet-adjusted low molecular weight heparin regimen. *Journal of thrombosis and thrombolysis, 46*(3), 386-392. doi:<https://dx.doi.org/10.1007/s11239-018-1711-5>

Huang, C., Wu, Z., Huang, W., Zhang, X., Lin, X., Luo, J., . . . Li, J. (2021). Identifying the impact of the Zone Insertion Method(TM) (ZIM(TM)): A randomized controlled trial. *J Vasc Access*, 11297298211052528. doi:10.1177/11297298211052528

Huang, V. (2018). Effect of a patency bundle on central venous catheter complications among hospitalized adult patients: a best practice implementation project. *JBI database of systematic reviews and implementation reports, 16*(2), 565-586. doi:<https://dx.doi.org/10.11124/JBISRIR-2016-003340>

Huang, W., & Xu, J. (2020). The role of sterile chitosan-based dressing in reducing complications related to a peripherally inserted central catheter in patients with hematological tumors. *Annals of palliative medicine, 9*(4), 2037-2044. doi:<https://dx.doi.org/10.21037/apm-20-1235>

Huihan, Z., Yu, H., Qin, W., & Yanping, Y. (2018). Medical Adhesive--Related Skin Injury Prevalence at the Peripherally Inserted Central Catheter Insertion Site: A Cross-sectional, Multiple-Center Study. *Journal of Wound, Ostomy & Continence Nursing, 45*(1), 22-25. doi:10.1097/WON.0000000000000394

Hyo-Cheol, K., Saebeom, H., & Hoyong, J. (2017). Malfunction of Totally Implantable Central Venous Ports. *Iranian Journal of Radiology, 14*(1), 1-Jul. doi:10.5812/iranjradiol.22046

Iftikhar, R., Chaudhry, Q. U. N., Satti, T. M., Mahmood, S. K., Satti, H. S., Ghafoor, T., & Khan, M. A. (2018). Noble Metal Coated Central Venous Catheters Are Not Superior To Uncoated Catheters In Preventing Infectious And Non-Infectious Complications In Immunocompromised Patients. *Journal of Ayub Medical College, Abbottabad : JAMC, 30(Suppl 1)*(4), S647-S651.

Imaoka, Y., Kuranishi, F., & Ogawa, Y. (2018). Usefulness of Totally Implantable Central Venous Access Devices in Elderly Patients: A Retrospective Study. *Annals of nutrition & metabolism, 72*(2), 112-116. doi:<https://dx.doi.org/10.1159/000486534>

Ince, M. E., Ozkan, G., Ors, N., Yildirim, A. K., & Doganci, S. (2020). Complications and pitfalls of central venous port catheters: experience with 782 patients with cancer. *Irish journal of medical science, 189*(4), 1371-1377. doi:<https://dx.doi.org/10.1007/s11845-020-02207-5>

Inoue, S., Yoshida, T., Nishino, T., Goto, M., Nishioka, K., Fujimoto, K., . . . Tangoku, A. (2020). Safe central venous catheters for esophageal cancer treatment. *The journal of medical investigation : JMI, 67*(3.4), 298-303. doi:<https://dx.doi.org/10.2152/jmi.67.298>

Isom, C., Bream, P., Gallagher, K., Walia, S., Ahmed, R., & Kauffmann, R. (2019). Placement of Subcutaneous Central Venous Ports in Breast Cancer Patients: Does Side Matter? *Journal of surgical research, 244*, 296-301. doi:<http://dx.doi.org/10.1016/j.jss.2019.06.028>

Jabaley, T., Xiong, N., Conley, S., Mazeika, T., Johnson, D., Biggins, B. A., . . . Hong, F. (2022). Transitioning from heparin to saline locks for central venous access devices in oncology: An evidence-based practice approach. *Can Oncol Nurs J, 32*(2), 286-293. doi:10.5737/23688076322286293

Jiang, M., Cui, X.-W., Li, C.-L., Pan, C.-Q., & Dietrich, C. F. (2020). Risk of venous thromboembolism associated with totally implantable venous access ports in cancer patients: A systematic review and meta-analysis. *Journal of Thrombosis and Haemostasis*. doi:<http://dx.doi.org/10.1111/jth.14930>

Jiang, M., Li, C. L., Pan, C. Q., & Yu, L. (2020). The risk of bloodstream infection associated with totally implantable venous access ports in cancer patient: a systematic review and meta-analysis. *Support Care Cancer, 28*(1), 361-372. doi:10.1007/s00520-019-04809-x

Johns, J., Wahlrab, L., & Elefritz, J. L. (2021). Acutely ill hematology/oncology patients with central-line associated bloodstream infections and the impact of timing of catheter removal on outcomes. *Am J Infect Control*. doi:10.1016/j.ajic.2021.10.038

Jones, D., Wismayer, K., Bozas, G., Palmer, J., Elliott, M., & Maraveyas, A. (2017). The risk of venous thromboembolism associated with peripherally inserted central catheters in ambulant cancer patients. *Thrombosis journal, 15*, 25. doi:<https://dx.doi.org/10.1186/s12959-017-0148-y>

Jones, M., Okano, S., Looke, D., Kennedy, G., Pavilion, G., Clouston, J., . . . Mollee, P. (2021). Catheter-associated bloodstream infection in patients with cancer: comparison of left- and right-sided insertions. *J Hosp Infect, 118*, 70-76. doi:10.1016/j.jhin.2021.10.008

Kagan, E., Salgado, C. D., Banks, A. L., Marculescu, C. E., & Cantey, J. R. (2019). Peripherally inserted central catheter-associated bloodstream infection: Risk factors and the role of antibiotic-impregnated catheters for prevention. *American journal of infection control, 47*(2), 191-195. doi:<https://dx.doi.org/10.1016/j.ajic.2018.07.006>

Kakkos, A., Bresson, L., Hudry, D., Cousin, S., Lervat, C., Bogart, E., . . . Narducci, F. (2017). Complication-related removal of totally implantable venous access port systems: Does the interval between placement and first use and the neutropenia-inducing potential of chemotherapy regimens influence their incidence? A four-year prospective study of 4045 patients. *European journal of surgical oncology : the journal of the European Society of Surgical Oncology and the British Association of Surgical Oncology, 43*(4), 689-695. doi:<https://dx.doi.org/10.1016/j.ejso.2016.10.020>

Kang, J., Chen, W., Sun, W., Ge, R., Li, H., Ma, E., . . . Liu, W. (2017). Peripherally inserted central catheter-related complications in cancer patients: a prospective study of over 50,000 catheter days. *The journal of vascular access, 18*(2), 153-157. doi:<https://dx.doi.org/10.5301/jva.5000670>

Kang, J. R., Long, L. H., Yan, S. W., Wei, W. W., Jun, H. Z., & Chen, W. (2017). Peripherally Inserted Central Catheter-Related Vein Thrombosis in Patients with Lung Cancer. *Clinical and applied thrombosis/hemostasis, 23*(2), 181-186. doi:<http://dx.doi.org/10.1177/1076029615595880>

Kao, C.-Y., Cheng, Y.-C., Chen, C. C.-C., Chai, J.-W., Fu, C.-H., Chen, J.-L., & Cheng, Y.-C. (2020). Outcome analysis in 270 radiologically guided implantations of totally implantable venous access ports via basilic vein. *Journal of the Chinese Medical Association, 83*(3), 295-301. doi:<http://dx.doi.org/10.1097/JCMA.0000000000000265>

Kao, P. F., Weng, J. H., Tyan, Y. S., Yang, S. F., & Tsao, T. C. (2017). The Incidence of Totally Implantable Venous Access Devices Insertion and the Associated Abnormalities in Patients With Cancer Revealed in (18)F-FDG PET-CT Imaging. *Acad Radiol, 24*(12), 1588-1595. doi:10.1016/j.acra.2017.06.017

Kara, H., Arikan, A. E., Dulgeroglu, O., Uras, C., Icten, G. E., Tutar, B., . . . Sonmez, O. (2021). Detachment and embolization of totally implantable central venous access devices: diagnosis and management. *Acta chirurgica Belgica*, 1-Aug. doi:<https://dx.doi.org/10.1080/00015458.2021.1896829>

Karapanou, A., Sampanis, M. A., Vieru, A.-M., Daikos, G. L., Samarkos, M., Pantazatou, A., & Deliolanis, I. (2020). Failure of central venous catheter insertion and care bundles in a high central line-associated bloodstream infection rate, high bed occupancy hospital. *American journal of infection control, 48*(7), 770-776. doi:<http://dx.doi.org/10.1016/j.ajic.2019.11.018>

Kato, Y., Hagihara, M., Kurumiya, A., Takahashi, T., Sakata, M., Shibata, Y., . . . Mikamo, H. (2018). Impact of mucosal barrier injury laboratory-confirmed bloodstream infection (MBI-LCBI) on central line-associated bloodstream infections (CLABSIs) in department of hematology at single university hospital in Japan. *Journal of infection and chemotherapy : official journal of the Japan Society of Chemotherapy, 24*(1), 31-35. doi:<https://dx.doi.org/10.1016/j.jiac.2017.08.013>

Kiesow, L., Mahnken, A. H., & Keulers, A. R. (2018). Port Implantation in Patients with Severe Thrombocytopenia is Safe with Interventional Radiology. *Cardiovascular and interventional radiology, 41*(1), 80-86. doi:<http://dx.doi.org/10.1007/s00270-017-1794-y>

Kikuchi, M., Sato, T., Okada, S., Abe, N., Sato, A., & Suzuki, Y. (2020). Maintenance antisepsis in reducing the rate of late-onset central venous catheter-related bloodstream infection: A comparison of 0.05% and 1% chlorhexidine. *Journal of infection and chemotherapy : official journal of the Japan Society of Chemotherapy, 26*(2), 188-193. doi:<https://dx.doi.org/10.1016/j.jiac.2019.08.007>

Kim, I. J., Shim, D. J., Byeon, J. H., Lee, J. H., Kim, E. T., Lee, H. J., & Cho, S. G. (2019). Impact of subcutaneous tunnels on peripherally inserted catheter placement: a multicenter retrospective study. *European radiology, 29*(5), 2716-2723. doi:<http://dx.doi.org/10.1007/s00330-018-5917-x>

Kim, J. H., Hong, Y. S., Kim, S. Y., Kim, K.-P., Choi, K. E., Kim, T. W., . . . Shin, J. H. (2018). Increased incidence of chemoport-related thrombosis in patients with colorectal cancer receiving bevacizumab: A single-institutional experience. *Chinese Journal of Cancer Research, 30*(4), 460-467. doi:<http://dx.doi.org/10.21147/j.issn.1000-9604.2018.04.09>

Kim, T. H., Choi, Y. W., Ahn, M. S., Choi, Y. S., Lee, H. W., Jeong, S. H., . . . Lee, H. Y. (2021). Early removal of central venous catheter may not impact the in-hospital mortality in patients with acute leukemia. *Ann Hematol, 100*(11), 2825-2830. doi:10.1007/s00277-021-04673-y

Kinoshita, M., Takao, S., Hiraoka, J., Takechi, K., Akagawa, Y., Osaki, K., . . . Tani, H. (2022). Risk factors for unsuccessful removal of central venous access ports implanted in the forearm of adult oncologic patients. *Jpn J Radiol, 40*(4), 412-418. doi:10.1007/s11604-021-01214-5

Kitamura, H., Kubota, Y., Komukai, S., Yoshida, H., Kaneko, Y., Mihara, Y., . . . Kimura, S. (2020). Venue of catheter insertion does not significantly impact the event of central line-associated bloodstream infection in patients with haematological diseases. *Infect Prev Pract, 2*(2), 100050. doi:10.1016/j.infpip.2020.100050

Kleidon, T. M., Horowitz, J., Ratz, D., Chopra, V., Rickard, C. M., Ullman, A. J., . . . Schults, J. (2021). Peripherally Inserted Central Catheter Thrombosis After Placement via Electrocardiography vs Traditional Methods. *American Journal of Medicine, 134*(2), e79-e88. doi:<http://dx.doi.org/10.1016/j.amjmed.2020.06.010>

Koo, C. M., Vissapragada, R., Sharp, R., Nguyen, P., Ung, T., Solanki, C., & Esterman, A. (2018). ABO blood group related venous thrombosis risk in patients with peripherally inserted central catheters. *The British journal of radiology, 91*(1082), 20170560. doi:<https://dx.doi.org/10.1259/bjr.20170560>

Kramer, R. D., Mann, J., Rogers, M. A. M., Saint, S., Chopra, V., & Conte, M. (2017). Are antimicrobial peripherally inserted central catheters associated with reduction in central line-associated bloodstream infection? A systematic review and meta-analysis. *American journal of infection control, 45*(2), 108-114. doi:<http://dx.doi.org/10.1016/j.ajic.2016.07.021>

Krumpelmann, U., Boseila, A., Lohnert, M., Kaup, O., Clarenbach, J. J., & Gorner, M. (2021). An analysis of totally implantable central venous port system infections in an urban tertiary referral center. *Journal of chemotherapy (Florence, Italy), 33*(4), 228-237. doi:<https://dx.doi.org/10.1080/1120009X.2020.1829327>

Kukla, M. E., Childs, C. A., Puig-Asensio, M., Marra, A. R., Perencevich, E. N., & Schweizer, M. L. (2020). Effectiveness of chlorhexidine dressings to prevent catheter-related bloodstream infections. Does one size fit all? A systematic literature review and meta-analysis. *Infection control and hospital epidemiology*. doi:<http://dx.doi.org/10.1017/ice.2020.356>

Kumwenda, M. J., Dougherty, L., Jackson, A., & Hill, S. (2021). Prospective Audit to Study urokinaSe use to restore Patency in Occluded centRal venous caTheters in haematology and oncology patients (PASSPORT 2). *The journal of vascular access, 22*(4), 568-574. doi:<https://dx.doi.org/10.1177/1129729820950997>

Lam, P. W., Volling, C., Chan, T., Wiggers, J. B., Castellani, L., Wright, J., . . . Leis, J. A. (2018). Impact of Defaulting to Single-Lumen Peripherally Inserted Central Catheters on Patient Outcomes: An Interrupted Time Series Study. *Clinical Infectious Diseases, 67*(6), 954-957. doi:<http://dx.doi.org/10.1093/cid/ciy301>

Lee, I. J., Kim, H. B., Choi, Y. J., Lee, J. H., Kim, E. T., Shim, D. J., . . . Lee, J. H. (2019). Prevalence and predictors of peripherally inserted central catheter-associated bloodstream infections in adults: A multicenter cohort study. *PloS one, 14*(3), e0213555. doi:<http://dx.doi.org/10.1371/journal.pone.0213555>

Lee, Y.-M., Ryu, B.-H., Hong, S. I., Cho, O.-H., Hong, K.-W., Bae, I.-G., . . . Park, K.-H. (2021). Clinical impact of early reinsertion of a central venous catheter after catheter removal in patients with catheter-related bloodstream infections. *Infection control and hospital epidemiology, 42*(2), 162-168. doi:<https://dx.doi.org/10.1017/ice.2020.405>

Lee, Y. M., Lee, M. S., Park, K. H., Moon, C., Kim, Y. J., & Lee, H. J. (2018). Clinical impact of delayed catheter removal for patients with central-venous-catheter-related Gram-negative bacteraemia. *Journal of Hospital Infection, 99*(1), 106-113. doi:<http://dx.doi.org/10.1016/j.jhin.2018.01.004>

Li, G., Zhang, Y., Ma, H., & Zheng, J. (2019). Arm port vs chest port: a systematic review and meta-analysis. *Cancer management and research, 11*, 6099-6112. doi:<https://dx.doi.org/10.2147/CMAR.S205988>

Lichtenstein, T., Rau, K., Hokamp, N. G., Maintz, D., Mammadov, K., Do, T. D., & Chang, D.-H. (2021). Long-term follow-up and clinical relevance of incidental findings of fibrin sheath and thrombosis on computed tomography scans of cancer patients with port catheters. *Therapeutics and clinical risk management, 17*, 111-118. doi:<http://dx.doi.org/10.2147/TCRM.S287544>

Lin, W. Y., Lin, C. P., Hsu, C. H., Lee, Y. H., Lin, Y. T., Hsu, M. C., & Shao, Y. Y. (2017). Right or left? Side selection for a totally implantable vascular access device: a randomised observational study. *British journal of cancer, 117*(7), 932‐937. doi:10.1038/bjc.2017.264

Lin, Y., Zeng, Z., Zheng, J., Lin, R., Liu, S., & Gao, X. (2020). The Caprini thrombosis risk model predicts the risk of PICC-related upper extremity venous thrombosis in cancer patients. *Journal of vascular surgery. Venous and lymphatic disorders*. doi:<http://dx.doi.org/10.1016/j.jvsv.2020.12.075>

Lingegowda, D., Gehani, A., Sen, S., Mukhopadhyay, S., & Ghosh, P. (2020). Centrally inserted tunnelled peripherally inserted central catheter: Off-label use for venous access in oncology patients. *The journal of vascular access, 21*(5), 773-777. doi:<https://dx.doi.org/10.1177/1129729820909028>

Liscynesky, C., Johnston, J., Haydocy, K. E., & Stevenson, K. B. (2017). Prospective evaluation of peripherally inserted central catheter complications in both inpatient and outpatient settings. *American journal of infection control, 45*(9), 1046-1049. doi:<https://dx.doi.org/10.1016/j.ajic.2017.02.006>

Liu, B., Wu, Z., Lin, C., Li, L., & Kuang, X. (2021). Applicability of TIVAP versus PICC in non-hematological malignancies patients: A meta-analysis and systematic review. *PloS one, 16*(8), e0255473. doi:10.1371/journal.pone.0255473

Liu, G. D., Ma, W. J., Liu, H. X., Tang, L., & Tan, Y. H. (2022). Risk factors associated with catheter-related venous thrombosis: a meta-analysis. *Public health, 205*, 45-54. doi:<https://dx.doi.org/10.1016/j.puhe.2022.01.018>

Liu, K., Zhou, Y., Xie, W., Chen, X., Wang, H., Gu, Z., . . . Cui, Y. (2018). Handgrip exercise reduces peripherally-inserted central catheter-related venous thrombosis in patients with solid cancers: A randomized controlled trial. *International journal of nursing studies, 86*, 99-106. doi:<http://dx.doi.org/10.1016/j.ijnurstu.2018.06.004>

Liu, S., Cong, L., Xiao, Z., Song, Y., Lou, T., Ma, Y., . . . Wang, N. (2019). Risk factors associated with peripherally inserted central catheter-related venous thrombosis in hospitalized patients of advanced age. *Journal of international medical research, 48*(1). doi:<http://dx.doi.org/10.1177/0300060518820744>

Liu, X., Tao, S., Ji, H., Chen, S., Gu, Y., & Jin, X. (2021). Risk factors for peripherally inserted central catheter (PICC)-associated infections in patients receiving chemotherapy and the preventive effect of a self-efficacy intervention program: a randomized controlled trial. *Ann Palliat Med, 10*(9), 9398-9405. doi:10.21037/apm-21-1848

Liu, Y., Li, L. L., Xu, L., Feng, D. D., Cao, Y., Mao, X. Y., . . . Chen, B. (2020). Comparison between Arm Port and Chest Port for Optimal Vascular Access Port in Patients with Breast Cancer: A Systematic Review and Meta-Analysis. *Biomed Res Int, 2020*, 9082924. doi:10.1155/2020/9082924

Liu, Z., Chen, J., Zan, L., Ding, S., Yi, H., & Yan, C. (2019). Exploring the risk factors of thrombosis and bloodstream infections in peripherally inserted central catheter (PICC) patients. *Journal of Biomaterials and Tissue Engineering, 9*(7), 929-934. doi:<http://dx.doi.org/10.1166/jbt.2019.2095>

Lo Priore, E., Fliedner, M., Heverhagen, J. T., Novak, U., & Marschall, J. (2017). The role of a surveillance programme for intro-ducing peripherally inserted central catheters: a 2-year observational study in an academic hospital. *Swiss medical weekly, 147*, w14441. doi:<https://dx.doi.org/10.4414/smw.2017.14441>

Longo, R., Llorens, M., Goetz, C., Platini, C., Eid, N., Sellies, J., . . . Quetin, P. (2017). Taurolidine/Citrate Lock Therapy for Primary Prevention of Catheter-Related Infections in Cancer Patients: Results of a Prospective, Randomized, Phase IV Trial (ATAPAC). *Oncology, 93*(2), 99-105. doi:<https://dx.doi.org/10.1159/000470911>

Lopez-Briz, E., Ruiz Garcia, V., Cabello, J. B., Bort-Marti, S., Carbonell Sanchis, R., & Burls, A. (2018). Heparin versus 0.9% sodium chloride locking for prevention of occlusion in central venous catheters in adults. *Cochrane Database of Systematic Reviews, 2018*(7), CD008462. doi:<http://dx.doi.org/10.1002/14651858.CD008462.pub3>

Low, X. Z., Tay, K. H., Leong, S., Lo, R. H. G., Zhuang, K. D., Chua, J. M. E., & Too, C. W. (2020). Repurposing the power injectable peripherally inserted central catheter as a tunnelled, non-cuffed, centrally inserted central venous catheter in oncological patients for short- to mid-term vascular access: A pilot study. *The journal of vascular access*, 1.12973E+12915. doi:<https://dx.doi.org/10.1177/1129729820943449>

Luong, N. V., Kroll, M. H., & Vu, K. (2017). Recurrence of venous thromboembolism among adults acute leukemia patients treated at the University of Texas MD Anderson Cancer Center: Incidence and risk factors. *Thrombosis research, 156*, 14-19. doi:<http://dx.doi.org/10.1016/j.thromres.2017.05.019>

Lv, L., Xu, J., Bai, C., Gong, J., Ma, W., & Sun, X. (2020). Cluster nursing in the prevention of PICC-related venous thrombosis and its influence on tumor patients' coagulation functions. *International journal of clinical and experimental medicine, 13*(12), 10005-10011.

Lv, Y., Hou, Y., Yu, L., Xu, D., Song, J., Shang, H., . . . Li, P. (2018). Risk associated with central catheters for malignant tumor patients: A systematic review and meta-analysis. *Oncotarget, 9*(15), 12376-12388. doi:<http://dx.doi.org/10.18632/oncotarget.24212>

Madabhavi, I., Patel, A., Anand, A., Kataria, P., Kadakol, N., & Sarkar, M. (2018). Use of Tunneled-Cuffed Central Catheters in Patients with Cancer: A Single-Center Experience. *JAVA - journal of the association for vascular access, 23*(1), 23-29. doi:<http://dx.doi.org/10.1016/j.java.2018.01.001>

Madabhavi, I., Patel, A., Anand, A., Panchal, H., Parikh, S., & Sarkar, M. (2017). A Study of Use of PORT Catheter in Patients with Cancer: A Single-Center Experience. *Clinical Medicine Insights: Oncology, 11*. doi:<http://dx.doi.org/10.1177/1179554917691031>

Madabhavi, I., Patel, A., Anand, A., Sarkar, M., Kataria, P., & Kadakol, N. (2018). A study of the use of peripherally inserted central catheters in cancer patients: A single-center experience. *Journal of vascular nursing : official publication of the Society for Peripheral Vascular Nursing, 36*(3), 149-156. doi:<http://dx.doi.org/10.1016/j.jvn.2018.05.001>

Malek, A. E., & Raad, II. (2020). Preventing catheter-related infections in cancer patients: a review of current strategies. *Expert Rev Anti Infect Ther, 18*(6), 531-538. doi:10.1080/14787210.2020.1750367

Mansour, A., Khozouz, O., Saadeh, S. S., Abunasser, M., Abdel-Razeq, N., & Taqash, A. (2018). Clinical Course and Complications of Catheter and Non-Catheter-Related Upper Extremity Deep Vein Thrombosis in Patients with Cancer. *Clinical and applied thrombosis/hemostasis, 24*(8), 1234-1240. doi:<http://dx.doi.org/10.1177/1076029618788177>

Mariggio, E., Iori, A. P., Micozzi, A., Chistolini, A., Latagliata, R., Berneschi, P., . . . Morano, S. G. (2020). Peripherally inserted central catheters in allogeneic hematopoietic stem cell transplant recipients. *Supportive care in cancer : official journal of the Multinational Association of Supportive Care in Cancer, 28*(9), 4193-4199. doi:<https://dx.doi.org/10.1007/s00520-019-05269-z>

Martinez, J. M., & Capela, R. (2021). INFUSION PUMP FLOW RATES IN CENTRAL VENOUS CATHETERS: THROMBUS REFLUX AND ASPIRATION CLOT. *Onco.news*(42), 16-20. doi:10.31877/on.2021.42.02

McDiarmid, S., Scrivens, N., Carrier, M., Sabri, E., Toye, B., Huebsch, L., & Fergusson, D. (2017). Outcomes in a nurse-led peripherally inserted central catheter program: a retrospective cohort study. *CMAJ open, 5*(3), E535-E539. doi:<https://dx.doi.org/10.9778/cmajo.20170010>

McDonald, M. K., Culos, K. A., Gatwood, K. S., Prow, C., Chen, H., Savani, B. N., . . . Satyanarayana, G. (2018). Defining Incidence and Risk Factors for Catheter-Associated Bloodstream Infections in an Outpatient Adult Hematopoietic Cell Transplantation Program. *Biology of blood and marrow transplantation : journal of the American Society for Blood and Marrow Transplantation, 24*(10), 2081-2087. doi:<https://dx.doi.org/10.1016/j.bbmt.2018.04.031>

McKeown, C., Ricciuti, A., Agha, M., Raptis, A., Hou, J. Z., Farah, R., . . . Boyiadzis, M. (2022). A prospective study of the use of central venous catheters in patients newly diagnosed with acute myeloid leukemia treated with induction chemotherapy. *Support Care Cancer, 30*(2), 1673-1679. doi:10.1007/s00520-021-06339-x

McParlan, D., Edgar, L., Gault, M., Gillespie, S., Menelly, R., & Reid, M. (2020). Intravascular catheter migration: A cross-sectional and health-economic comparison of adhesive and subcutaneous engineered stabilisation devices for intravascular device securement. *The journal of vascular access, 21*(1), 33-38. doi:<https://dx.doi.org/10.1177/1129729819851059>

Michell, H., Nezami, N., Morris, C., & Hong, K. (2021). Dual-chambered venous access port as alternative access for extracorporeal apheresis therapy. *The journal of vascular access, 22*(2), 173-177. doi:<https://dx.doi.org/10.1177/1129729820932425>

Mielke, D., Wittig, A., & Teichgraber, U. (2020). Peripherally inserted central venous catheter (PICC) in outpatient and inpatient oncological treatment. *Supportive care in cancer : official journal of the Multinational Association of Supportive Care in Cancer, 28*(10), 4753-4760. doi:<https://dx.doi.org/10.1007/s00520-019-05276-0>

Milani, A., Mazzocco, K., Gandini, S., Pravettoni, G., Libutti, L., Zencovich, C., . . . Saiani, L. (2017). Incidence and Determinants of Port Occlusions in Cancer Outpatients: A Prospective Cohort Study. *Cancer nursing, 40*(2), 102-107. doi:<https://dx.doi.org/10.1097/NCC.0000000000000357>

Mittal, G. S., Sundriyal, D., Naik, N. B., & Sehrawat, A. (2021). Totally Implantable Venous Access Device (Chemoport) in Oncology: Study of 168 Polyurethane Chemoport Catheter System. *South Asian J Cancer, 10*(4), 261-264. doi:10.1055/s-0041-1739041

Mollee, P., Abro, E., Van Kuilenburg, R., Joubert, W., Okano, S., Looke, D., . . . Jones, M. (2020). Catheter-associated bloodstream infections in adults with cancer: a prospective randomized controlled trial. *Journal of Hospital Infection, 106*(2), 335-342. doi:<http://dx.doi.org/10.1016/j.jhin.2020.07.021>

Moralar, D. G., Turkmen, U. A., Bilen, A., Turkmen, S., Feyizi, H., & Altan, H. A. (2021). Our central venous port catheter system practice - a retrospective study. *J Pak Med Assoc, 71*(5), 1442-1445. doi:10.47391/jpma.03-240

Moseley, J. M., Sachs, O., Chugh, P., Oneal, P. B., He, K., Whang, E., & Kristo, G. (2021). Survival and Complications After Placement of Central Venous Access Ports for Palliative Chemotherapy: A Single-Institution Retrospective Analysis. *The American journal of hospice & palliative care*, 1.04991E+04916. doi:<http://dx.doi.org/10.1177/10499091211002127>

Moss, J. G., Wu, O., Bodenham, A. R., Agarwal, R., Menne, T. F., Jones, B. L., . . . McCartney, E. (2021). Central venous access devices for the delivery of systemic anticancer therapy (CAVA): a randomised controlled trial. *Lancet, 398*(10298), 403-415. doi:10.1016/s0140-6736(21)00766-2

Nakamura, T., Sasaki, J., Asari, Y., Sato, T., Torii, S., & Watanabe, M. (2017). Complications after implantation of subcutaneous central venous ports (PowerPort R). *Annals of medicine and surgery (2012), 17*, 1-Jun. doi:<https://dx.doi.org/10.1016/j.amsu.2017.03.014>

Nezami, N., Groenwald, M., Silin, D., Latich, I., Xing, M., & Kokabi, N. (2019). Risk Factors of Infection and Role of Antibiotic Prophylaxis in Totally Implantable Venous Access Port Placement: Propensity Score Matching. *Cardiovascular and interventional radiology, 42*(9), 1302-1310. doi:<http://dx.doi.org/10.1007/s00270-019-02255-0>

Nucci, M., Braga, P. R., Nouer, S. A., & Anaissie, E. (2018). Time of catheter removal in candidemia and mortality. *The Brazilian journal of infectious diseases : an official publication of the Brazilian Society of Infectious Diseases, 22*(6), 455-461. doi:<https://dx.doi.org/10.1016/j.bjid.2018.10.278>

Oh, S. B., Park, K., Kim, J. J., Oh, S. Y., Jung, K. S., Park, B. S., . . . Lee, S. S. (2021). Safety and feasibility of 3-month interval access and flushing for maintenance of totally implantable central venous port system in colorectal cancer patients after completion of curative intended treatments. *Medicine (Baltimore), 100*(2), e24156. doi:10.1097/md.0000000000024156

Ohtake, S., Nakagawa, M., Uchino, Y., Miura, K., Iriyama, N., Hatta, Y., . . . Nakayama, T. (2018). One percent chlorhexidine-alcohol for preventing central venous catheter-related infection during intensive chemotherapy for patients with haematologic malignancies. *Journal of Infection and Chemotherapy, 24*(7), 544-548. doi:<http://dx.doi.org/10.1016/j.jiac.2018.03.001>

Okazaki, M., Oyama, K., Kinoshita, J., Miyashita, T., Tajima, H., Takamura, H., . . . Ohta, T. (2019). Incidence of and risk factors for totally implantable vascular access device complications in patients with gastric cancer: A retrospective analysis. *Molecular and clinical oncology, 11*(4), 343-348. doi:<https://dx.doi.org/10.3892/mco.2019.1897>

Paquet, F., Boucher, L. M., Valenti, D., & Lindsay, R. (2017). Impact of arm selection on the incidence of PICC complications: results of a randomized controlled trial. *Journal of vascular access, 18*(5), 408‐414. doi:10.5301/jva.5000738

Park, E. J., Park, K., Kim, J.-J., Oh, S.-B., Jung, K. S., Oh, S. Y., . . . Jeon, U.-B. (2021). Safety, Efficacy, and Patient Satisfaction with Initial Peripherally Inserted Central Catheters Compared with Usual Intravenous Access in Terminally Ill Cancer Patients: A Randomized Phase II Study. *Cancer research and treatment, 53*(3), 881-888. doi:<https://dx.doi.org/10.4143/crt.2020.1008>

Park, E. J., Park, K., Kim, J. J., Oh, S. B., Jung, K. S., Oh, S. Y., . . . Jeon, U. B. (2020). Safety, Efficacy, and Patient Satisfaction with Initial Peripherally Inserted Central Catheters Compared with Usual Intravenous Access in Terminally Ill Cancer Patients: a Randomized Phase II Study. *Cancer research and treatment*. doi:10.4143/crt.2020.1008

Park, S., Moon, S., Pai, H., & Kim, B. (2020). Appropriate duration of peripherally inserted central catheter maintenance to prevent central line-associated bloodstream infection. *PloS one, 15*(6), e0234966. doi:<https://dx.doi.org/10.1371/journal.pone.0234966>

Patel, P. A., Boehm, S., Zhou, Y., Zhu, C., Peterson, K. E., Grayes, A., & Peterson, L. R. (2017). Prospective observational study on central line-associated bloodstream infections and central venous catheter occlusions using a negative displacement connector with an alcohol disinfecting cap. *American journal of infection control, 45*(2), 115-120. doi:<https://dx.doi.org/10.1016/j.ajic.2016.06.013>

Peng, S. Y., Wei, T., Li, X. Y., Yuan, Z., & Lin, Q. (2022). A model to assess the risk of peripherally inserted central venous catheter-related thrombosis in patients with breast cancer: a retrospective cohort study. *Support Care Cancer, 30*(2), 1127-1137. doi:10.1007/s00520-021-06511-3

Pénichoux, J., Rio, J., Kammoun, L., Vermeulin, T., Pepin, L. F., Camus, V., . . . Jardin, F. (2022). Retrospective analysis of the safety of peripherally inserted catheters versus implanted port catheters during first-line treatment for patients with diffuse large B-cell lymphoma. *Eur J Haematol, 109*(1), 41-49. doi:10.1111/ejh.13767

Perek, S., Khatib, A., Izhaki, N., Khalaila, A. S., Brenner, B., & Horowitz, N. A. (2022). A prediction model for central venous catheter-related thrombosis in patients with newly-diagnosed acute myeloid leukemia: A derivation cohort analysis. *Eur J Intern Med*. doi:10.1016/j.ejim.2022.04.025

Picardi, M., Della Pepa, R., Cerchione, C., Pugliese, N., Mortaruolo, C., Trastulli, F., . . . Pane, F. (2019). A Frontline Approach With Peripherally Inserted Versus Centrally Inserted Central Venous Catheters for Remission Induction Chemotherapy Phase of Acute Myeloid Leukemia: A Randomized Comparison. *Clinical lymphoma, myeloma & leukemia, 19*(4), e184-e194. doi:<https://dx.doi.org/10.1016/j.clml.2018.12.008>

Pike, S., Tan, K., & Burbridge, B. (2021). Complications Associated With Totally Implanted Venous Access Devices in the Arm Versus the Chest: A Short-Term Retrospective Study. *Can Assoc Radiol J*, 8465371211040822. doi:10.1177/08465371211040822

Pinelli, F., Balsorano, P., Mura, B., & Pittiruti, M. (2021). Reconsidering the GAVeCeLT Consensus on catheter-related thrombosis, 13 years later. *The journal of vascular access, 22*(4), 501-508. doi:<https://dx.doi.org/10.1177/1129729820947594>

Pinelli, F., Pittiruti, M., Van Boxtel, T., Barone, G., Biffi, R., Capozzoli, G., . . . Pepe, G. (2021). GAVeCeLT-WoCoVA Consensus on subcutaneously anchored securement devices for the securement of venous catheters: Current evidence and recommendations for future research. *The journal of vascular access, 22*(5), 716-725. doi:<https://dx.doi.org/10.1177/1129729820924568>

Piredda, A., Radice, D., Zencovich, C., Cerri, M., Aventino, L., Naccarato, F., . . . Biffi, R. (2021). Safe use of Peripherally Inserted Central Catheters for chemotherapy of solid malignancies in adult patients: A 1-year monocentric, prospectively-assessed, unselected cohort of 482 patients. *The journal of vascular access, 22*(6), 873-881. doi:<https://dx.doi.org/10.1177/1129729820962905>

Platanaki, C., Zareifopoulos, N., Lagadinou, M., Tsiotsios, K., & Velissaris, D. (2021). Correlation of Positive Blood Cultures with Peripherally Inserted Central Catheter Line Infection in Oncology Patients. *Cureus, 13*(1), e12858. doi:10.7759/cureus.12858

Ploton, G., Brebion, N., Guyomarch, B., Pistorius, M. A., Connault, J., Hersant, J., . . . Espitia, O. (2021). Predictive factors of venous recanalization in upper-extremity vein thrombosis. *PloS one, 16*(5), e0251269. doi:10.1371/journal.pone.0251269

Pu, Y. L., Li, Z. S., Zhi, X. X., Shi, Y. A., Meng, A. F., Cheng, F., . . . Wang, C. (2020). Complications and Costs of Peripherally Inserted Central Venous Catheters Compared With Implantable Port Catheters for Cancer Patients: A Meta-analysis. *Cancer Nurs, 43*(6), 455-467. doi:10.1097/ncc.0000000000000742

Qi, F., Cheng, H., Yuan, X., & Zhang, L. (2020). Comparison of PICC and TIVAP in chemotherapy for patients with thyroid cancer. *Oncology letters, 20*(2), 1657-1662. doi:<https://dx.doi.org/10.3892/ol.2020.11732>

Raad, S., Chaftari, A. M., Hachem, R. Y., Shah, P., Natividad, E., Cleeland, C. S., & Rosenblatt, J. (2018). Removal and insertion of central venous catheters in cancer patients is associated with high symptom burden. *Expert review of medical devices, 15*(8), 591-596. doi:<https://dx.doi.org/10.1080/17434440.2018.1500892>

Rabelo-Silva, E. R., Lourenço, S. A., Maestri, R. N., Candido da Luz, C., Carlos Pupin, V., Bauer Cechinel, R., . . . Chopra, V. (2022). Patterns, appropriateness and outcomes of peripherally inserted central catheter use in Brazil: a multicentre study of 12 725 catheters. *BMJ Qual Saf*. doi:10.1136/bmjqs-2021-013869

Ranch-Lundin, M., Schedin, A., & Bjorkhem-Bergman, L. (2021). Equal effect of vancomycin lock with or without heparin in treatment of central venous catheter related blood stream infections–an observational study in palliative home care. *Infectious Diseases, 53*(9), 719‐723. doi:10.1080/23744235.2021.1922752

Rasero, L., Golin, L., Ditta, S., Di Massimo, D. S., Dal Molin, A., & Piemonte, G. (2018). Effects of prolonged flushing interval in totally implantable venous access devices (TIVADs). *British journal of nursing (Mark Allen Publishing), 27*(8), S4-S10. doi:<https://dx.doi.org/10.12968/bjon.2018.27.8.S4>

Rickard, C. M., Flynn, J., Larsen, E., Mihala, G., Playford, E. G., Shaw, J., . . . Marsh, N. (2021). Needleless connector decontamination for prevention of central venous access device infection: A pilot randomized controlled trial. *American journal of infection control, 49*(2), 269-273. doi:10.1016/j.ajic.2020.07.026

Rickard, C. M., Marsh, N. M., Webster, J., Gavin, N. C., Chan, R. J., McCarthy, A. L., . . . et al. (2017). Peripherally InSerted CEntral catheter dressing and securement in patients with cancer: the PISCES trial. Protocol for a 2x2 factorial, superiority randomised controlled trial. *BMJ open, 7*(6), e015291. doi:10.1136/bmjopen-2016-015291

Rixecker, T., Lesan, V., Ahlgrimm, M., Thurner, L., Bewarder, M., Murawski, N., . . . Kaddu-Mulindwa, D. (2021). Insertion site of central venous catheter correlates with catheter-related infectious events in patients undergoing intensive chemotherapy. *Bone marrow transplantation, 56*(1), 195-201. doi:<https://dx.doi.org/10.1038/s41409-020-01003-0>

Rockholt, M. M., Thorarinsdottir, H. R., Lazarevic, V., Rundgren, M., & Kander, T. (2022). Central venous catheter-related complications in hematologic patients: An observational study. *Acta Anaesthesiol Scand, 66*(4), 473-482. doi:10.1111/aas.14020

Rowe, M. S., Arnold, K., & Spencer, T. R. (2020). Catheter securement impact on PICC-related CLABSI: A university hospital perspective. *American journal of infection control, 48*(12), 1497-1500. doi:10.1016/j.ajic.2020.06.178

Ruiz-Giardin, J. M., Ochoa Chamorro, I., Velazquez Rios, L., Jaqueti Aroca, J., Garcia Arata, M. I., SanMartin Lopez, J. V., & Guerrero Santillan, M. (2019). Blood stream infections associated with central and peripheral venous catheters. *BMC infectious diseases, 19*(1), 841. doi:<https://dx.doi.org/10.1186/s12879-019-4505-2>

Russo, R., Oliveira, M. S., Shikanai-Yasuda, M. A., Mendes, E. T., Levin, A. S., Costa, S. F., & Dulley, F. (2019). Bloodstream infection in hematopoietic stem cell transplantation outpatients: Risk factors for hospitalization and death. *Revista do Instituto de Medicina Tropical de Sao Paulo, 61*, e3. doi:<http://dx.doi.org/10.1590/s1678-9946201961003>

Sacks, O. A., Chugh, P., He, K., Moseley, J. M., Oneal, P. B., Whang, E., & Kristo, G. (2022). Survival and Complications After Placement of Central Venous Access Ports for Palliative Chemotherapy: A Single-Institution Retrospective Analysis. *Am J Hosp Palliat Care, 39*(1), 34-38. doi:10.1177/10499091211002127

Samuelson, C., Kaur, H., Kritsotakis, E. I., Goode, S. D., Nield, A., & Partridge, D. (2018). A daily topical decontamination regimen reduces catheter-related bloodstream infections in haematology patients. *The Journal of infection, 76*(2), 132-139. doi:<https://dx.doi.org/10.1016/j.jinf.2017.10.014>

Santacatalina-Roig, E., Espinar-de Las Heras, E., Ballesteros-Lizondo, J. M., Ibanez-Puchades, I., & Pescador-Marco, J. L. (2020). Peripherally inserted central catheter in haematopoietic stem cell transplantation. Infusion of haematopoietic cells and complications. *Cateter central de insercion periferica en trasplante de progenitores hematopoyeticos. Infusion de celulas hematopoyeticas y complicaciones., 30*(5), 295-301. doi:<https://dx.doi.org/10.1016/j.enfcli.2019.09.016>

Sapkota, S., Sannur, R., & Naik, R. (2020). Analysis of Peripherally Inserted Central Catheter Line in Cancer Patients: A Single-Center Experience. *South Asian J Cancer, 9*(4), 253-256. doi:10.1055/s-0040-1721175

Schears, G. J., Ferko, N., Syed, I., Arpino, J.-M., & Alsbrooks, K. (2021). Peripherally inserted central catheters inserted with current best practices have low deep vein thrombosis and central line-associated bloodstream infection risk compared with centrally inserted central catheters: A contemporary meta-analysis. *Journal of vascular access, 22*(1), Sep-25. doi:<http://dx.doi.org/10.1177/1129729820916113>

Scrivens, N., Sabri, E., Bredeson, C., & McDiarmid, S. (2020). Comparison of complication rates and incidences associated with different peripherally inserted central catheters (PICC) in patients with hematological malignancies: a retrospective cohort study. *Leukemia & lymphoma, 61*(1), 156-164. doi:<https://dx.doi.org/10.1080/10428194.2019.1646908>

Seckold, T., Walker, S., Dwyer, T., & Signal, T. (2019). Peripherally Inserted Central Catheter Postinsertion Complications: A Retrospective Study. *Journal of the Association for Vascular Access, 24*(1), Oct-20. doi:10.1016/j.java.2018.25.003

Sengul, T., Ocakci, A. F., Guven, B., & Kaya, N. (2019). Connectors as a risk factor for blood-associated infections (3-way stopcock and needleless connector): A randomized-experimental study. *American journal of infection control*. doi:<http://dx.doi.org/10.1016/j.ajic.2019.08.020>

Seo, T. S., Song, M. G., Kim, J. S., Choi, C. W., Seo, J. H., Oh, S. C., . . . Lee, S. Y. (2017). Long-term clinical outcomes of the single-incision technique for implantation of implantable venous access ports via the axillary vein. *J Vasc Access, 18*(4), 345-351. doi:10.5301/jva.5000751

Sharp, R., Carr, P., Childs, J., Scullion, A., Young, M., Flynn, T., . . . Esterman, A. (2021). Catheter to vein ratio and risk of peripherally inserted central catheter (PICC)-associated thrombosis according to diagnostic group: a retrospective cohort study. *BMJ open, 11*(7), e045895. doi:10.1136/bmjopen-2020-045895

Shibata, J., Kawamura, H., Hiramatsu, K., Honda, M., Shibata, Y., Aoba, T., . . . Kato, T. (2021). Impact of chest subcutaneous fat on the occurrence of central venous port-related infectious complications in cancer patients. *Support Care Cancer, 29*(9), 5391-5398. doi:10.1007/s00520-021-06109-9

Shih, Y. H., Teng, C. J., Chen, T. C., Chang, K. H., & Chen, M. H. (2022). Dual-lumen power injectable peripherally inserted central catheters in allogeneic hematopoietic stem cell transplantation: A prospective observational study. *J Clin Nurs, 31*(11-12), 1654-1661. doi:10.1111/jocn.16020

Silva, S. R. D., Reichembach, M. T., Pontes, L., Souza, G., & Kusma, S. (2021). Heparin solution in the prevention of occlusions in Hickman® catheters a randomized clinical trial. *Revista latino-americana de enfermagem, 29*, e3385. doi:10.1590/1518-8345.3310.3385

Simonetti, G., Bersani, A., Tramacere, I., Lusignani, M., Gaviani, P., & Silvani, A. (2022). The role of body mass index in the development of thromboembolic events among cancer patients with PICCs: a systematic review. *J Vasc Nurs, 40*(1), 11-16. doi:10.1016/j.jvn.2021.10.001

Simonetti, G., Sommariva, A., Lusignani, M., Anghileri, E., Ricci, C. B., Eoli, M., . . . Silvani, A. (2020). Prospective observational study on the complications and tolerability of a peripherally inserted central catheter (PICC) in neuro-oncological patients. *Supportive care in cancer : official journal of the Multinational Association of Supportive Care in Cancer, 28*(6), 2789-2795. doi:<https://dx.doi.org/10.1007/s00520-019-05128-x>

Skelton Iv, W. P., Franke, A. J., Welniak, S., Bosse, R. C., Ayoub, F., Murphy, M., & Starr, J. S. (2019). Investigation of Complications Following Port Insertion in a Cancer Patient Population: A Retrospective Analysis. *Clinical Medicine Insights: Oncology, 13*, N.PAG-N.PAG. doi:10.1177/1179554919844770

Skummer, P., Kobayashi, K., DeRaddo, J. S., Blackburn, T., Schoeneck, M., Patel, J., & Jawed, M. (2020). Risk Factors for Early Port Infections in Adult Oncologic Patients. *Journal of vascular and interventional radiology : JVIR, 31*(9), 1427-1436. doi:<https://dx.doi.org/10.1016/j.jvir.2020.05.018>

Slaughter, E., Keogh, S. J., Kynoch, K., & Brodribb, M. (2020). Evaluating the Impact of Central Venous Catheter Materials and Design on Thrombosis: A Systematic Review and Meta-Analysis. *Worldviews on evidence-based nursing, 17*(5), 376-384. doi:<http://dx.doi.org/10.1111/wvn.12472>

Snarski, E., Stringer, J., Mikulska, M., Gil, L., Tridello, G., Bosman, P., . . . Styczynski, J. (2021). Risk of infectious complications in adult patients after allogeneic hematopoietic stem cell transplantation depending on the site of central venous catheter insertion-multicenter prospective observational study, from the IDWP EBMT and Nurses Group of EBMT. *Bone Marrow Transplant, 56*(12), 2929-2933. doi:10.1038/s41409-021-01430-7

Solinas, G., Platini, F., Trivellato, M., Rigo, C., Alabiso, O., & Galetto, A. S. (2017). Port in oncology practice: 3-monthly locking with normal saline for catheter maintenance, a preliminary report. *The journal of vascular access, 18*(4), 325-327. doi:<https://dx.doi.org/10.5301/jva.5000740>

Song, X., Lu, H., Chen, F., Bao, Z., Li, S., Li, S., . . . Zhang, W. (2020). A longitudinal observational retrospective study on risk factors and predictive model of PICC associated thrombosis in cancer patients. *Scientific reports, 10*(1), 10090. doi:<https://dx.doi.org/10.1038/s41598-020-67038-x>

Song, Y., Liu, S., Lou, T., Ma, Y., Wang, N., Yong, Q., . . . Liu, G. (2020). Risk factors associated with peripherally inserted central catheter-related venous thrombosis in hospitalized patients of advanced age. *The Journal of international medical research, 48*(1), 3.00061E+00014. doi:<https://dx.doi.org/10.1177/0300060518820744>

Spires, S. S., Rebeiro, P. F., Miller, M., Koss, K., Wright, P. W., & Talbot, T. R. (2018). Medically Attended Catheter Complications Are Common in Patients With Outpatient Central Venous Catheters. *Infection control and hospital epidemiology, 39*(4), 439-444. doi:<https://dx.doi.org/10.1017/ice.2018.8>

Suleman, A., Jarvis, V., Hadziomerovic, A., Carrier, M., & McDiarmid, S. (2019). Implanted vascular access device related deep vein thrombosis in oncology patients: A prospective cohort study. *Thrombosis research, 177*, 117-121. doi:<https://dx.doi.org/10.1016/j.thromres.2019.02.033>

Suleman, A., & McDiarmid, S. (2017). A Retrospective Analysis of Catheter-Related Upper Extremity Deep Vein Thrombosis in Peripherally Inserted Catheters With and Without a Dermatotomy. *JAVA - journal of the association for vascular access, 22*(4), 178-181. doi:<http://dx.doi.org/10.1016/j.java.2017.07.005>

Suttle, R. D., Buffington, H. M., Madden, W. T., & Dawson, M. A. (2019). Central Line Care: Empowering Patients to Prevent Infection and Injury Via EPIC2. *Clinical journal of oncology nursing, 23*(1), E10-E16. doi:<https://dx.doi.org/10.1188/19.CJON.E10-E16>

Sze Yong, T., Vijayanathan, A. A., Chung, E., Ng, W. L., Yaakup, N. A., & Sulaiman, N. (2022). Comparing catheter related bloodstream infection rate between cuffed tunnelled and non-cuffed tunnelled peripherally inserted central catheter. *J Vasc Access, 23*(2), 225-231. doi:10.1177/1129729820987373

Tabatabaie, O., Kasumova, G. G., Eskander, M. F., Critchlow, J. F., Tawa, N. E., & Tseng, J. F. (2017). Totally Implantable Venous Access Devices: A Review of Complications and Management Strategies. *American journal of clinical oncology, 40*(1), 94-105. doi:<https://dx.doi.org/10.1097/COC.0000000000000361>

Tabatabaie, O., Kasumova, G. G., Kent, T. S., Eskander, M. F., Fadayomi, A. B., Ng, S. C., . . . Tseng, J. F. (2017). Upper extremity deep venous thrombosis after port insertion: What are the risk factors? *Surgery, 162*(2), 437-444. doi:<https://dx.doi.org/10.1016/j.surg.2017.02.020>

Takashima, M., Ray-Barruel, G., Ullman, A., Keogh, S., & Rickard, C. M. (2017). Randomized controlled trials in central vascular access devices: A scoping review. *PloS one, 12*(3), e0174164. doi:<https://dx.doi.org/10.1371/journal.pone.0174164>

Tan, L., Sun, Y., Zhu, L., Lei, X., Liang, D., Rao, N., . . . Li, S. (2019). Risk factors of catheter-related thrombosis in early-stage breast cancer patients: a single-center retrospective study. *Cancer management and research, 11*, 8379-8389. doi:<https://dx.doi.org/10.2147/CMAR.S212375>

Tang, L., Kim, C. Y., Martin, J. G., Pabon-Ramos, W. M., Sag, A. A., Suhocki, P. V., . . . Ronald, J. (2020). Length of Stay Predicts Risk of Early Infection for Hospitalized Patients Undergoing Central Venous Port Placement. *Journal of vascular and interventional radiology : JVIR, 31*(3), 454-461. doi:<https://dx.doi.org/10.1016/j.jvir.2019.10.017>

Tang, T., Li, H., Wang, J., Li, C., & Geng, C. (2019). The causes and managements of catheter misplacement in implantable vascular access devices: A retrospective analysis of 8534 patients in a single center. *International journal of clinical and experimental medicine, 12*(9), 11864-11868.

Taxbro, K., Hammarskjold, F., Thelin, B., Lewin, F., Hagman, H., Hanberger, H., & Berg, S. (2019). Clinical impact of peripherally inserted central catheters vs implanted port catheters in patients with cancer: an open-label, randomised, two-centre trial. *British journal of anaesthesia, 122*(6), 734-741. doi:<https://dx.doi.org/10.1016/j.bja.2019.01.038>

Tian, L., Yin, X., Zhu, Y., Zhang, X., & Zhang, C. (2021). Analysis of Factors Causing Skin Damage in the Application of Peripherally Inserted Central Catheter in Cancer Patients. *J Oncol, 2021*, 6628473. doi:10.1155/2021/6628473

Tippit, D., Ananthula, A., Siegel, E., Ochoa, D., Hill, E., Merrill, A., . . . Makhoul, I. (2018). Upper-Extremity Deep Vein Thrombosis in Patients With Breast Cancer With Chest Versus Arm Central Venous Port Catheters. *Breast Cancer: Basic and Clinical Research, 12*. doi:<http://dx.doi.org/10.1177/1178223418771909>

Trezza, C., Califano, C., Iovino, V., D'Ambrosio, C., Grimaldi, G., & Pittiruti, M. (2021). Incidence of fibroblastic sleeve and of catheter-related venous thrombosis in peripherally inserted central catheters: A prospective study on oncological and hematological patients. *The journal of vascular access, 22*(3), 444-449. doi:<https://dx.doi.org/10.1177/1129729820949411>

Tsuruta, S., Goto, Y., Miyake, H., Nagai, H., Yoshioka, Y., Yuasa, N., & Takamizawa, J. (2020). Late complications associated with totally implantable venous access port implantation via the internal jugular vein. *Supportive care in cancer : official journal of the Multinational Association of Supportive Care in Cancer, 28*(6), 2761-2768. doi:<https://dx.doi.org/10.1007/s00520-019-05122-3>

Tumay, L. V., & Guner, O. S. (2021). Availability of totally implantable venous access devices in cancer patients is high in the long term: a seven-year follow-up study. *Supportive care in cancer : official journal of the Multinational Association of Supportive Care in Cancer, 29*(7), 3531-3538. doi:<https://dx.doi.org/10.1007/s00520-020-05871-6>

Ullman, A. J., Mihala, G., O'Leary, K., Marsh, N., Woods, C., Bugden, S., . . . Rickard, C. M. (2019). Skin complications associated with vascular access devices: A secondary analysis of 13 studies involving 10,859 devices. *International journal of nursing studies, 91*, Jun-13. doi:10.1016/j.ijnurstu.2018.10.006

Ullman, A. J., Paterson, R. S., Schults, J. A., Kleidon, T. M., August, D., O'Malley, M., . . . Chopra, V. (2022). Do antimicrobial and antithrombogenic peripherally inserted central catheter (PICC) materials prevent catheter complications? An analysis of 42,562 hospitalized medical patients. *Infect Control Hosp Epidemiol, 43*(4), 427-434. doi:10.1017/ice.2021.141

Velioglu, Y., Yuksel, A., & Sinmaz, E. (2019). Complications and management strategies of totally implantable venous access port insertion through percutaneous subclavian vein. *Turk gogus kalp damar cerrahisi dergisi, 27*(4), 499-507. doi:<https://dx.doi.org/10.5606/tgkdc.dergisi.2019.17972>

Verboom, M. C., Ouwerkerk, J., Gelderblom, H., Steeghs, N., Kerst, J. M., Lutjeboer, J., . . . Sleijfer, S. (2017). Central venous access related adverse events after trabectedin infusions in soft tissue sarcoma patients// experience and management in a nationwide multi-center study. *Clinical Sarcoma Research, 7*(1), 2. doi:<http://dx.doi.org/10.1186/s13569-017-0066-6>

Vermeulin, T., Lucas, M., Marini, H., Di Fiore, F., Loeb, A., Lottin, M., . . . Merle, V. (2018). Totally implanted venous access-associated adverse events in oncology: Results from a prospective 1-year surveillance programme. *Bulletin du cancer, 105*(11), 1003-1011. doi:<https://dx.doi.org/10.1016/j.bulcan.2018.09.005>

Voog, E., Bourgeois, H., Domont, J., Denis, F., Emmanuel, E., Dupuis, O., . . . Campion, L. (2018). Totally implantable venous access ports: a prospective long-term study of early and late complications in adult patients with cancer. *Supportive Care in Cancer, 26*(1), 81-89. doi:<http://dx.doi.org/10.1007/s00520-017-3816-3>

Voor in ’t holt, A. F., Helder, O. K., Vos, M. C., Schafthuizen, L., Sülz, S., van den Hoogen, A., & Ista, E. (2017). Antiseptic barrier cap effective in reducing central line-associated bloodstream infections: A systematic review and meta-analysis. *International journal of nursing studies, 69*, 34-40. doi:10.1016/j.ijnurstu.2017.01.007

Wan, R., Gu, L., Yin, B., Cai, S., Zhou, R., & Yang, W. (2022). A six-year study of complications related to peripherally inserted central catheters: A multi-center retrospective cohort study in China. *Perfusion*, 2676591221076287. doi:10.1177/02676591221076287

Wang, G.-D., Wang, H.-Z., Shen, Y.-F., Dong, J., Wang, X.-P., Wang, X.-Z., . . . Guo, S.-S. (2020). The influence of venous characteristics on peripherally inserted central catheter-related symptomatic venous thrombosis in cancer patients. *Cancer management and research, 12*, 11909-11920. doi:<http://dx.doi.org/10.2147/CMAR.S282370>

Wang, G., Li, Y., Wu, C., Guo, L., Hao, L., Liao, H., . . . Luo, L. (2020). The clinical features and related factors of PICC-related upper extremity asymptomatic venous thrombosis in cancer patients: A prospective study. *Medicine, 99*(12), e19409. doi:<https://dx.doi.org/10.1097/MD.0000000000019409>

Wang, G., Wang, H., Shen, Y., Dong, J., Wang, X., Wang, X., . . . Guo, S. (2021). Association between ABO blood group and venous thrombosis related to the peripherally inserted central catheters in cancer patients. *The journal of vascular access, 22*(4), 590-596. doi:<https://dx.doi.org/10.1177/1129729820954721>

Wang, X.-J. (2017). Preventive effect of dexamethasone solution pre-treated catheter on PICC-induced phlebitis. *Biomedical research (india), 28*(12), 5310-5314.

Wang, Y. C., Lin, P. L., Chou, W. H., Lin, C. P., & Huang, C. H. (2017). Long-term outcomes of totally implantable venous access devices. *Support Care Cancer, 25*(7), 2049-2054. doi:10.1007/s00520-017-3592-0

Webber, J. L. R., & Maningo-Salinas, M. J. (2020). Sticking It to Them--Reducing Migration of Peripherally Inserted Central Catheters. *Journal of the Association for Vascular Access, 25*(1), Oct-15. doi:10.2309/j.java.2020.001.004

Webster, J., Larsen, E., Marsh, N., Choudhury, A., Harris, P., & Rickard, C. M. (2017). Chlorhexidine gluconate or polyhexamethylene biguanide disc dressing to reduce the incidence of central-line-associated bloodstream infection: a feasibility randomized controlled trial (the CLABSI trial). *The Journal of hospital infection, 96*(3), 223-228. doi:<https://dx.doi.org/10.1016/j.jhin.2017.04.009>

Winkler, M. A., Spencer, T. R., Siddiqi, N., Wallace, J. E., Gallien, J. Z., Elbalasi, H., . . . Raissi, D. (2021). Clinical experience with a chlorhexidine-coated PICC: A prospective, multicenter, observational study. *J Vasc Access*, 11297298211049648. doi:10.1177/11297298211049648

Wu, S., Li, W., Zhang, Q., Li, S., & Wang, L. (2018). Comparison of complications between peripheral arm ports and central chest ports: A meta-analysis. *Journal of advanced nursing, 74*(11), 2484-2496. doi:<https://dx.doi.org/10.1111/jan.13766>

Wu, X., Zhang, T., Chen, L., & Chen, X. (2021). Prolonging the flush-lock interval of totally implantable venous access ports in patients with cancer: A systematic review and meta-analysis. *The journal of vascular access, 22*(5), 814-821. doi:<https://dx.doi.org/10.1177/1129729820950998>

Xie, J., Xu, L., Xu, X., & Huang, Y. (2017). Complications of peripherally inserted central catheters in advanced cancer patients undergoing combined radiotherapy and chemotherapy. *Journal of clinical nursing, 26*(23-24), 4726-4733. doi:<https://dx.doi.org/10.1111/jocn.13825>

Xiong, Z. Y., Luo, Z., & Chen, H. Y. (2019). Prevalence of idle peripherally inserted central catheters in adult patients: A multicenter cross-sectional study. *J Vasc Access, 20*(6), 677-682. doi:10.1177/1129729819840917

Xiong, Z. Y., Zhou, H. M., & Li, S. Y. (2021). Prolonged flushing and locking interval for totally implantable vascular access device: A systematic review and meta-analysis. *J Vasc Access*, 1.12973E+12916. doi:10.1177/11297298211003003

Yan, W., Zhang, C., Luo, C., & Li, Z. (2021). Management of outpatient with totally implantable venous access Ports during the COVID-19 epidemic. *Medicine, 100*(7), e24720. doi:<https://dx.doi.org/10.1097/MD.0000000000024720>

Yang, S.-S., & Ahn, M. S. (2018). A Comparison Between Upper Arm and Chest for Optimal Site of Totally Implanted Venous Access Ports in Patients with Female Breast Cancer. *Annals of vascular surgery, 50*, 128-134. doi:<http://dx.doi.org/10.1016/j.avsg.2017.11.059>

Yang, W. J., Song, M. G., Seo, T. S., & Park, S. J. (2021). Effectiveness of mechanical recanalization for intraluminal occlusion of totally implantable venous access ports. *J Vasc Access*, 11297298211034628. doi:10.1177/11297298211034628

Yanik, F., Karamustafaoglu, Y. A., Karatas, A., & Yoruk, Y. (2018). Experience in totally implantable venous port catheter: Analysis of 3,000 patients in 12 years. *Turk gogus kalp damar cerrahisi dergisi, 26*(3), 422-428. doi:<https://dx.doi.org/10.5606/tgkdc.dergisi.2018.15299>

Yildiz, A., Albayrak, M., Sahin, O., Pala, C., Ozturk, H. B. A., Gunes, G., . . . Okutan, H. (2019). Incidence and risk factors of port related infections in patients with hematological malignancy. *International journal of clinical and experimental medicine, 12*(1), 989-996.

Yin, L., & Li, J. (2020). Central Venous Catheter Insertion in Colorectal Cancer Patients, PICC or PC? *Cancer management and research, 12*, 5813-5818. doi:<https://dx.doi.org/10.2147/CMAR.S250410>

Yin, Y. X., Gao, W., Li, X. Y., Lu, W., Deng, Q. H., Zhao, C. Y., . . . Zhang, H. J. (2020). Randomized multicenter study on long-term complications of peripherally inserted central catheters positioned by electrocardiographic technique. *Phlebology / Venous Forum of the Royal Society of Medicine*. doi:10.1177/0268355520921357

Ying, S., Liping, Z., Yanhong, D., Zhulin, G., & Liang, G. (2020). Impact of arm choice for peripherally inserted central catheter (PICC) insertion on patients: a cross-sectional study. *Contemporary nurse, 56*(1), 80-89. doi:<http://dx.doi.org/10.1080/10376178.2020.1741417>

Yu, L., Zhang, R., Li, J., Yan, X., Jin, K., Li, W., & Jiang, G. (2017). Incidence and risk factors for peripherally inserted central catheter-related vein thrombosis in lung cancer patients. *International journal of clinical and experimental medicine, 10*(8), 12440-12446.

Yu, X. Y., Xu, J. L., Li, D., & Jiang, Z. F. (2018). Late complications of totally implantable venous access ports in patients with cancer: Risk factors and related nursing strategies. *Medicine (Baltimore), 97*(38), e12427. doi:10.1097/md.0000000000012427

Yun, W. S., & Yang, S. S. (2021). Comparison of peripherally inserted central catheters and totally implanted venous access devices as chemotherapy delivery routes in oncology patients: A retrospective cohort study. *Sci Prog, 104*(2), 368504211011871. doi:10.1177/00368504211011871

Zabicki, B., Limphaibool, N., Veilemand Holstad, M. J., & Perkowska, K. (2019). Central venous access ports in the interventional radiology suite - One-centre experience. *Polish journal of radiology, 84*, e328-e334. doi:<http://dx.doi.org/10.5114/pjr.2019.88066>

Zanwar, S., Gokarn, A., Devadas, S. K., Punatar, S., Khurana, S., Bonda, A., . . . Bhat, V. (2019). Antibiotic lock therapy for salvage of tunneled central venous catheters with catheter colonization and catheter-related bloodstream infection. *Transplant Infectious Disease, 21*(1), e13017. doi:<http://dx.doi.org/10.1111/tid.13017>

Zerla, P. A., Canelli, A., Cerne, L., Caravella, G., Gilardini, A., De Luca, G., . . . Venezia, R. (2017). Evaluating safety, efficacy, and cost-effectiveness of PICC securement by subcutaneously anchored stabilization device. *The journal of vascular access, 18*(3), 238-242. doi:<https://dx.doi.org/10.5301/jva.5000655>

Zhang, M., Kang, L., & Li, Q. (2017). A comparative study on the use of different connectors in tube sealing in elderly tumor patients with PICC. *International journal of clinical and experimental medicine, 10*(6), 9488-9494.

Zhang, S., Kobayashi, K., Faridnia, M., Skummer, P., Zhang, D., & Karmel, M. I. (2018). Clinical Predictors of Port Infections in Adult Patients with Hematologic Malignancies. *Journal of vascular and interventional radiology : JVIR, 29*(8), 1148-1155. doi:<https://dx.doi.org/10.1016/j.jvir.2018.04.014>

Zhang, Y., Zhang, S., Chen, J., & Zhao, R. (2021). Blood sampling from peripherally inserted central catheter is effective and safe for patients with head and neck cancers. *The journal of vascular access, 22*(3), 424-431. doi:<https://dx.doi.org/10.1177/1129729820943458>

Zhang, Y., Zhao, R., Jiang, N., Shi, Y., Wang, Q., & Sheng, Y. (2021). A retrospective observational study on maintenance and complications of totally implantable venous access ports in 563 patients: Prolonged versus short flushing intervals. *Int J Nurs Sci, 8*(3), 252-256. doi:10.1016/j.ijnss.2021.05.005

Zhao, H., He, Y., Huang, H., Ling, Y., Zhou, X., Wei, Q., . . . Ying, Y. (2018). Prevalence of medical adhesive-related skin injury at peripherally inserted central catheter insertion site in oncology patients. *The journal of vascular access, 19*(1), 23-27. doi:<https://dx.doi.org/10.5301/jva.5000805>

Zhao, Y., Bian, L., & Yang, J. (2022). Intervention efficacy of MARSI nursing management on skin injury at peripherally inserted central catheter insertion site on oncological patients. *Int Wound J*. doi:10.1111/iwj.13805

Zhong, J., Wang, B., & Huang, Q. (2021). Study on treating tumor patients with a peripherally inserted central catheter. *International journal of clinical and experimental medicine, 14*(1), 683‐689.

Zhou, H., Yang, B., Wang, C., & Qin, Y. (2017). Analysis and clinical significance of venography findings in complications associated with peripherally inserted central catheters. *Biomedical research (india), 28*(15), 6619-6625.

Ziegler, M., Landsburg, D., Kucharczuk, C., Gorman, T., Bink, K., Stadtmauer, E. A., . . . Han, J. H. (2019). Fluoroquinolone Prophylaxis Is Highly Effective for the Prevention of Central Line-Associated Bloodstream Infections in Autologous Stem Cell Transplant Patients. *Biology of blood and marrow transplantation, 25*(5), 1004-1010. doi:<http://dx.doi.org/10.1016/j.bbmt.2018.11.023>
