# Supplementary material for: Central venous access device terminologies, complications, and reason for removal in oncology: a scoping review
Source: BMC Cancer. 2024 Apr 19;24:498. doi: 10.1186/s12885-024-12099-8 (PMC11027380; doi:10.1186/s12885-024-12099-8)
Supplement: Supplementary file 4 — Additional file 4. Summary of CVAD terminology. [file 12885_2024_12099_MOESM4_ESM.docx]

**Additional file 4: Unique terminology**

| **Unique names for CVAD** (n=14) | | **Number of studies** |
| --- | --- | --- |
| 1 | central venous catheters | 96 |
| 2 | central venous access devices | 26 |
| 3 | vascular access devices | 6 |
| 4 | central lines | 3 |
| 5 | venous access devices | 3 |
| 6 | central venous access | 2 |
| 7 | central venous devices | 1 |
| 8 | central catheters | 1 |
| 9 | intravascular catheters | 1 |
| 10 | venous catheters | 1 |
| 11 | conventional percutaneously inserted devices | 1 |
| 12 | intravascular devices | 1 |
| 13 | lines | 1 |
| 14 | long-term venous access devices | 1 |
|  |  |  |
| **Unique names for portacaths** (n=104) | | **Number of studies** |
| 1 | totally implantable venous access ports | 20 |
| 2 | ports | 14 |
| 3 | totally implantable venous access devices | 13 |
| 4 | implantable ports | 9 |
| 5 | implanted ports | 9 |
| 6 | central venous ports | 6 |
| 7 | port catheters | 6 |
| 8 | totally implanted venous access devices | 6 |
| 9 | arm ports | 5 |
| 10 | chest ports | 5 |
| 11 | central venous port catheters | 4 |
| 12 | totally implantable vascular access devices | 4 |
| 13 | central venous access port | 3 |
| 14 | Centrally inserted totally implanted vascular access ports | 3 |
| 15 | implantable venous access ports | 3 |
| 16 | portacaths | 3 |
| 17 | totally implantable central venous access ports | 3 |
| 18 | totally implanted ports | 3 |
| 19 | totally implanted vascular access devices | 3 |
| 20 | brachial implanted central venous ports | 2 |
| 21 | central venous port systems | 2 |
| 22 | chemoport | 2 |
| 23 | implanted port catheters | 2 |
| 24 | Implanted vascular access devices | 2 |
| 25 | intravenous ports | 2 |
| 26 | Subcutaneous implanted port catheters | 2 |
| 27 | totally implantable central venous access devices | 2 |
| 28 | totally implantable central venous catheter | 2 |
| 29 | totally implantable central venous port systems | 2 |
| 30 | totally implantable ports | 2 |
| 31 | totally implantable venous access port systems | 2 |
| 32 | totally implanted venous access ports | 2 |
| 33 | venous access ports | 2 |
| 34 | catheters | 1 |
| 35 | central intravenous infusion devices | 1 |
| 36 | central venous access port devices | 1 |
| 37 | central venous access ports | 1 |
| 38 | chemotherapy ports | 1 |
| 39 | Chest-to-arm ports | 1 |
| 40 | devices | 1 |
| 41 | Dual-chambered venous access port | 1 |
| 42 | fully implantable catheters | 1 |
| 43 | fully implantable device | 1 |
| 44 | groin-ports | 1 |
| 45 | Implantable central venous ports | 1 |
| 46 | Implantable Port Catheters | 1 |
| 47 | implantable venous access devices | 1 |
| 48 | implanted PICC-ports | 1 |
| 49 | implanted port systems | 1 |
| 50 | implanted port-a-caths | 1 |
| 51 | implanted venous access devices | 1 |
| 52 | implanted venous port | 1 |
| 53 | infusion port | 1 |
| 54 | non-power-injectable ports | 1 |
| 55 | peripheral "PORTs" | 1 |
| 56 | peripheral central venous access ports | 1 |
| 57 | peripherally inserted central catheter-PORT | 1 |
| 58 | Permanent Port Catheters | 1 |
| 59 | PICC-ports | 1 |
| 60 | PORT catheter devices | 1 |
| 61 | port lines | 1 |
| 62 | Port-a-Cath | 1 |
| 63 | port-acath lines | 1 |
| 64 | portcath system | 1 |
| 65 | port-catheters | 1 |
| 66 | power-injectable port | 1 |
| 67 | PowerPorts | 1 |
| 68 | subclavian venous port catheter | 1 |
| 69 | Subcutaneous Central Venous Ports | 1 |
| 70 | subcutaneous chest ports | 1 |
| 71 | Subcutaneous Implantable Venous Access Devices | 1 |
| 72 | subcutaneous implanted catheters | 1 |
| 73 | Subcutaneous implanted port-a-cath catheters | 1 |
| 74 | subcutaneous implanted ports | 1 |
| 75 | subcutaneous port catheters | 1 |
| 76 | subcutaneous ports with tunneled catheters | 1 |
| 77 | Subcutaneous venous access devices | 1 |
| 78 | subcutaneous venous chest port catheter | 1 |
| 79 | subcutaneous venous port | 1 |
| 80 | subcutaneous venous ports tunneled | 1 |
| 81 | surgically implantable catheters | 1 |
| 82 | totally implantable access ports | 1 |
| 83 | Totally Implantable Catheters | 1 |
| 84 | totally implantable central venous port catheters | 1 |
| 85 | totally implantable central venous ports | 1 |
| 86 | totally implantable CVCs | 1 |
| 87 | totally implantable venous access | 1 |
| 88 | Totally implantable venous access devices catheter | 1 |
| 89 | totally implantable venous access systems | 1 |
| 90 | totally implantable venous port catheters | 1 |
| 91 | totally implantable venous portal systems | 1 |
| 92 | totally implanted catheters | 1 |
| 93 | totally implanted central venous access devices | 1 |
| 94 | totally implanted central venous catheters | 1 |
| 95 | totally implanted centrally inserted ports | 1 |
| 96 | totally implanted CVADs | 1 |
| 97 | totally implanted venous access | 1 |
| 98 | totally implanted venous access devices systems | 1 |
| 99 | upper-extremity port | 1 |
| 100 | upper-limb-ports | 1 |
| 101 | vascular port catheters | 1 |
| 102 | venous ports | 1 |
| 103 | venous port systems | 1 |
| 104 | Vital-Port Titanium Mini vascular access systems | 1 |
|  |  |  |
| **Unique names for PICCs** (n=25) | | **Number of studies** |
| 1 | peripherally inserted central catheters | 142 |
| 2 | peripherally inserted central venous catheters | 9 |
| 3 | peripherally inserted catheters | 3 |
| 4 | peripherally inserted central catheter line | 2 |
| 5 | peripherally inserted CVCs | 2 |
| 6 | Groshong catheters | 2 |
| 7 | central peripheral insertion catheter | 1 |
| 8 | conventional peripherally inserted central catheter | 1 |
| 9 | non-cuffed tunnelled peripherally inserted central catheter | 1 |
| 10 | cuffed tunnelled peripherally inserted central catheter | 1 |
| 11 | dual-lumen power injectable peripherally inserted central catheters | 1 |
| 12 | multiple-lumen catheters | 1 |
| 13 | non tunneled, non cuffed peripherally inserted central catheters | 1 |
| 14 | nonantimicrobial-impregnated catheters | 1 |
| 15 | non-tunnelled single-lumen catheters | 1 |
| 16 | peripheral inserted central catheters | 1 |
| 17 | peripheral insertion central catheters | 1 |
| 18 | peripheral venous catheters | 1 |
| 19 | peripherally inserted central lines | 1 |
| 20 | peripherally inserted CVC (PICC) lines | 1 |
| 21 | PICC lines | 1 |
| 22 | single lumen catheters | 1 |
| 23 | tunneled and nontunneled peripherally inserted central catheter | 1 |
| 24 | tunneled peripherally inserted central catheter | 1 |
| 25 | antimicrobial-impregnated PICC | 1 |
|  |  |  |
| **Unique names for a tunnelled cuffed-CICC** (n=41) | | **Number of studies** |
| 1 | Hickman lines | 13 |
| 2 | Hickman catheters | 10 |
| 3 | tunnelled catheters | 8 |
| 4 | Hickmans | 4 |
| 5 | tunneled CVAD | 3 |
| 6 | tunneled CVCs | 3 |
| 7 | Broviac catheters | 2 |
| 8 | long-term CVCs | 2 |
| 9 | tunneled catheters with central insertion | 2 |
| 10 | Tunneled central venous catheter | 2 |
| 11 | tunnelled-cuffed centrally inserted central catheters | 2 |
| 12 | cuffed totally implantable catheters | 1 |
| 13 | cuffed tunnelled catheter | 1 |
| 14 | cuffed tunnelled central catheters | 1 |
| 15 | Hickman (cuffed) catheter | 1 |
| 16 | Hickmann tunneled central catheter | 1 |
| 17 | Hickman-type | 1 |
| 18 | Hickman-type central venous catheters | 1 |
| 19 | Hickman-type tunnelled catheters | 1 |
| 20 | indwelling long-term central venous catheter | 1 |
| 21 | long-term catheters | 1 |
| 22 | longterm central venous catheter | 1 |
| 23 | long-term centrally inserted catheters | 1 |
| 24 | long-term indwelling CVCs | 1 |
| 25 | long-term intravascular catheters | 1 |
| 26 | long-term nontunneled CVC | 1 |
| 27 | long-term tunneled catheter | 1 |
| 28 | long-term tunneled CVC | 1 |
| 29 | percutaneous tunneled CVCs | 1 |
| 30 | permanent CVC | 1 |
| 31 | semi-implanted central venous catheter | 1 |
| 32 | skin-tunnelled catheter | 1 |
| 33 | tunneled central catheters | 1 |
| 34 | tunneled cuffed catheters | 1 |
| 35 | tunneled cuffed CVC | 1 |
| 36 | tunneled cuffed CVAD | 1 |
| 37 | Tunneled-Cuffed Central Catheters | 1 |
| 38 | Tunnelled (Hickman®) | 1 |
| 39 | tunnelled central lines | 1 |
| 40 | tunnelled central venous access devices | 1 |
| 41 | tunnelled central venous catheter | 1 |
|  |  |  |
| **Unique names for CICCs** (n=27) | | **Number of studies** |
| 1 | centrally inserted central catheters | 10 |
| 2 | central venous catheters | 6 |
| 3 | non tunneled CVC | 4 |
| 4 | non-tunneled central venous catheters | 4 |
| 5 | nontunneled catheters | 3 |
| 6 | central lines | 2 |
| 7 | non-tunneled CVAD | 2 |
| 8 | non-tunnelled catheters | 2 |
| 9 | short-term central venous catheters | 2 |
| 10 | central catheters | 1 |
| 11 | central inserted central catheter | 1 |
| 12 | Central venous catheter (non-tunnelled) | 1 |
| 13 | Central venous catheters devices | 1 |
| 14 | clavicularly inserted venous catheter | 1 |
| 15 | external central venous catheters | 1 |
| 16 | external CVCs | 1 |
| 17 | external non-tunneled central venous catheters | 1 |
| 18 | non tunneled, non cuffed CVCs | 1 |
| 19 | non-tunneled centrally inserted central catheters | 1 |
| 20 | non-tunnelled central venous access devices | 1 |
| 21 | non-tunnelled centrally inserted central venous catheter | 1 |
| 22 | percutaneous central venous catheters | 1 |
| 23 | percutaneous non-cuffed CVCs | 1 |
| 24 | short-term CVCs | 1 |
| 25 | short-term non-tunneled CVCs | 1 |
| 26 | temporary CVC | 1 |
| 27 | thin-lumen, non-tunnelled access devices | 1 |
|  |  |  |
| **Unique names for FICCs** (n=2) | | **Number of studies** |
| 1 | femorally inserted central catheters | 3 |
| 2 | femoral inserted central catheters | 1 |
